# Harnessing Negative Photochromism in Styryl Cyanines for Light‐Modulated Proton Transport

**DOI:** 10.1002/anie.202506532

**Published:** 2025-05-16

**Authors:** Gianni Pacella, Maria Nabatova, Yuxuan Zhang, David Picconi, Roza R. Weber, Shirin Faraji, Giuseppe Portale

**Affiliations:** ^1^ Institute of Theoretical and Computational Chemistry Heinrich‐Heine University Düsseldorf Düsseldorf Germany; ^2^ Zernike Institute for Advanced Materials University of Groningen Nijemborg 3 Groningen 9757AG Netherlands; ^3^ Center for Systems Chemistry Stratigh Institute for Chemistry University of Groningen Nijemborg 3 Groningen 9757AG Netherlands

**Keywords:** Acidochromism, Negative photochromism, Photoswitches, Proton conductivity, Styryl Cyanine

## Abstract

Efficient photoswitches capable of complete conversion to their metastable isomer are not so common, yet highly desirable for applications in smart materials and devices. Here, we report the photochromic behavior of a series of styryl cyanine photoswitches, all demonstrating high switching efficiency, with some achieving full conversion to the metastable form. Despite structural similarities to spiropyran photoswitches, we demonstrate that the photochemistry of styryl cyanines is fundamentally different. Unlike classical photoswitches that rely on double‐bond rotation, these molecules undergo substantial geometric changes via the formation of a spiro carbon. This transformation disrupts conjugation, causing a desirable blue shift in absorbance ideal for creating responsive materials and devices. We further show that the switching kinetics can be finely tuned through electronic effects of various substituents or the choice of surrounding medium. These photoswitches exhibit excellent fatigue resistance and can be easily shifted into the visible region via their acidochromic properties. Taking advantage of their high switching efficiency and affinity for acidic polymers, we finally propose their use as smart dopants to develop light‐responsive materials with tunable proton transport properties under visible light irradiation.

## Graphical abstract

We show how photo switchable styryl cyanines undergo an extremely efficient photochemical ring‐closing reaction upon irradiation. Furthermore, substituents on the ring and the nature of their surroundings can be used to tune their chemistry, kinetics, and photochemical properties. Finally, we demonstrate how these molecules can be used as smart dopants to control proton transport using light in a polymeric matrix.

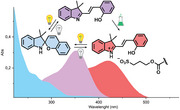

## Introduction

Molecular photoswitches are a class of molecules that make use of light to induce a molecular transformation that changes their absorbance spectra, and therefore their color.^[^ ^1^ ^]^ The most common light‐induced transformation is the E/Z isomerization of a double bond such as C═C in stilbenes^[^ ^2^ ^]^ and stiff stilbenes,^[^ ^3^ ^]^ N═N in azobenzenes^[^ ^4^ ^]^ and diazocines,^[^ ^5^ ^]^ or C═N in hydrazones,^[^ ^6^ ^]^ and in imines.^[^ ^7^ ^]^ In all these cases, the absorbance is affected by the geometrical changes induced by the double bond isomerization. Alternatively, light‐triggered pericyclic reactions (which cause large geometrical changes) can also induce changes in the absorbance spectrum of molecules and directly affect the molecule's conjugation, such as in the case of spiropyrans^[^ ^8^, ^9^, ^10^ ^]^ and its derivatives,^[^ ^11^, ^12^ ^]^ diarylethenes,^[^ ^13^ ^]^ or fulgides.^[^ ^14^ ^]^

Within the family of photoswitches, the ones whose absorbance is blue‐shifted upon irradiation with light of a suitable wavelength are defined as negatively photochromic.^[^ ^15^ ^]^ Photochromes with this characteristic are considered to be particularly useful since the absorbance of the two isomers does not overlap, and therefore an efficient and quantitative switching can be achieved. In addition, molecules exhibiting negative photochromism^[^ ^16^ ^]^ are particularly appealing for applications in light‐responsive materials and devices, because irradiation increases the transparency of the material, facilitating deeper light penetration and therefore more efficient switching through the material. Unfortunately, only a limited amount of photoswitches that are capable of blue shifting their absorbance upon light irradiation are available, and this category is surely dominated by spiropyran derivatives,^[^ ^12^, ^15^, ^17^, ^18^ ^]^ even if not limited to them.^[^ ^19^, ^20^, ^21^ ^]^

**Figure 1:**
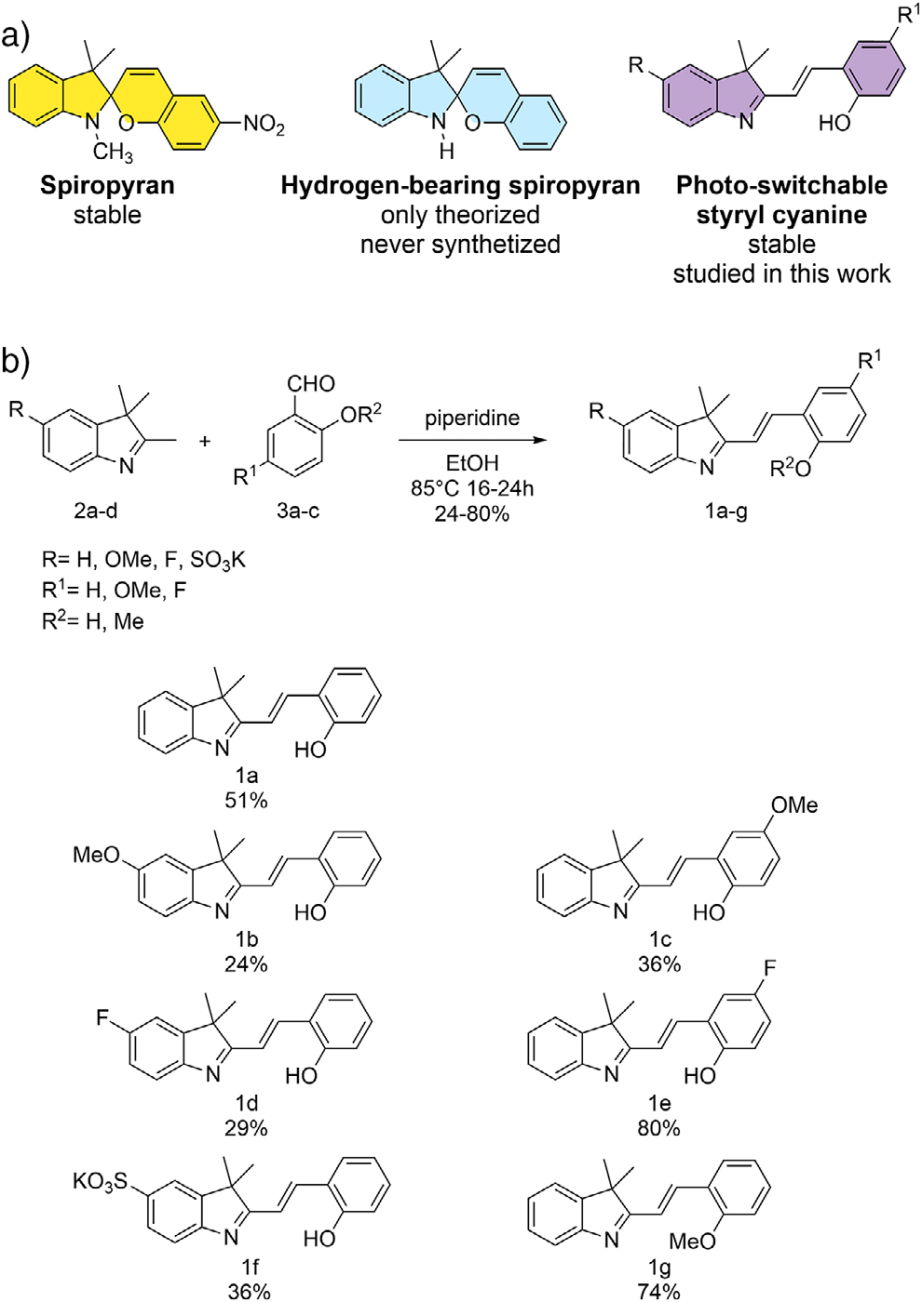
a) Comparison between classic spiropyrans (in yellow), hydrogen‐bearing spiropyran which were only theorized and never actually synthesized (in cyan), and styryl cyanine, the light‐responsive molecule family explored in this work (in purple). b) Synthetic procedure utilized to prepare the photo switchable molecules **1a–g**, and structures of the studied styryl cyanines.

Spiropyran photoswitches (SP) are characterized by the presence of a spiro carbon which links their indoline and chromene parts together. The sp^3^ spiro carbon is then converted to sp^2^ upon irradiation with ultraviolet light to generate the conjugated, zwitterionic merocyanine open form.^[^ ^8^, ^10^ ^]^ Another distinguishing feature of SP is that the indoline nitrogen is tertiary in the closed form (Figure 1a). To the best of our knowledge, spiropyran molecules in which the nitrogen is substituted with hydrogen instead of the classic carbon (Figure 1a) were only theorized,^[^ ^22^ ^]^ and never actually synthesized. In fact, when attempting the synthesis of hydrogen‐bearing spiropyran, the formation of styryl cyanine 1a molecule occurs instead (Figure 1a,b). Even though styryl cyanines resemble in their chemical structure a spiropyran photoswitch, they do not present a spiro centre in their low energy state. Although ortho‐hydroxy substituted styryl cyanines have been reported to be mechano‐chromic molecules^[^ ^23^, ^24^ ^]^ or used as fluorescent probes,^[^ ^25^ ^]^ they have never been explored as photoswitches, similarly to what previously happened with diazocines.^[^ ^5^ ^]^ Because of their peculiar chemical structure, styryl cyanines could thus present photochromism, which has not been explored so far.

For these reasons, in this work, we synthesized molecules 1a–g (Figure 1b), and we have investigated their response to light and pH. We show how molecules 1a–f present inverted photochromism compared to the parent spiropyran and undergo a light‐promoted cyclization reaction to form their closed corresponding “spiro” isomers. This behavior strongly differs from any previously reported photochromic dye with a similar chemical structure^[^ ^26^, ^27^ ^]^ which simply undergoes E/Z isomerization of their double bond upon irradiation. Our results show that these molecules have interesting negative photochromic behavior which allows them to achieve up to 100% switching. Moreover, we also show how the back isomerization kinetics of the studied molecules can be tuned using electronic effects or the surrounding medium. Owing to their unique properties, we finally demonstrate their potential as smart dopants in acidic polymers, enabling the preparation of polymeric films with tuneable proton translocation behavior when placed in the dark or irradiated with light.

## Results and Discussion

Photoswitchable styryl cyanine derivatives **1a–g** have been synthesized by Knoevenagel condensation of a series of indolenines (**2a–d**) and salicylaldehyde derivatives (**3a–d**) (Figure 1b and Section 2.2 Supporting Information). Their preparation is straightforward and **1a–f** can be easily purified by precipitation in an appropriate antisolvent. These characteristics make this new class of photoswitches particularly appealing for applications in which the preparation of large quantities of materials is required, i.e., material chemistry, optoelectronics, etc.

SP have been widely studied in the past decades because of their outstanding UV light‐triggered properties, such as a change in color and a remarkable change in dipole moment. However, they also present limitations such as low fatigue resistance,^[^ ^28^, ^29^, ^30^, ^31^ ^]^ and most importantly the fact that the absorbance spectra of the closed spiro form and the open merocyanine form partially overlap at the wavelength of irradiation (typically 365 nm), making it impossible to quantitatively convert the closed spiropyran to its open merocyanine form. This is usually not a problem for protonated merocyanines^[^ ^32^ ^]^ (MCH) since they present a characteristic peak between 400 and 480 nm that disappears upon irradiation with blue light, making a quantitative conversion to their closed spiropyran form possible. Moreover, the fatigue resistance of protonated MCH is remarkable^[^ ^33^ ^]^ but their most concerning limitation is their moderate solubility in aqueous media and, most importantly, their insolubility in aprotic organic solvents,^[^ ^34^ ^]^ which restricts their use as photoswitches in water or polar protic solvent‐based systems.

**Figure 2:**
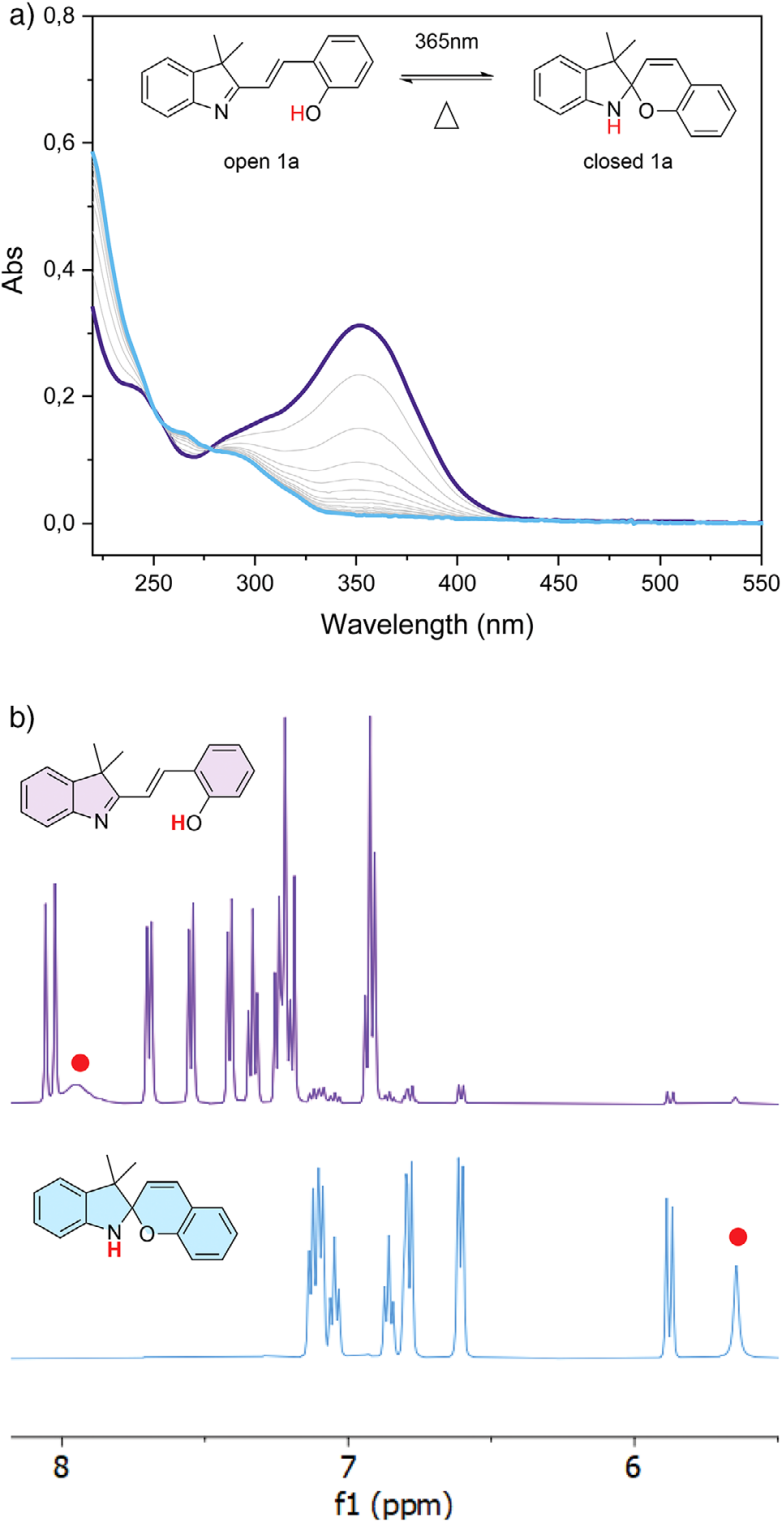
a) UV–vis absorbance kinetic spectra for the photoisomerization of open 1a (0.02 mM, 20 °C, and CH_3_CN) to closed 1a in acetonitrile. b) Zoom of the aromatic region in the 1H‐NMR spectra (500 MHz, 298 K) of 1a open and 1a closed in CD_3_CN. Purple = acquired in the dark, Cyan = PSS_365_. The red dots mark the phenolic proton in the top spectra, and the appearance the signal associated with the new proton from the amino group in the bottom spectra.

We found that similarly to the family of protonated merocyanines (MCH),^[^ ^12^, ^35^ ^]^ the light‐induced isomerization of styryl cyanines **1a–f** bearing a hydroxy group in the ortho position of the styryl part takes place through the E‐to‐Z isomerization of the double bond followed by a nucleophilic attack of the OH group with the consequence formation of a new C─O bond. We followed this isomerization process through UV–vis spectroscopy (Figure 2a). Upon irradiation with 365 nm light, we observe the disappearance of the peak centred at 362 nm (associated with the open form of derivative **1a**), and an increased absorbance in the region between 220 and 250 nm, corresponding to the closed isomer of **1a**. Once the photo stationary state (PSS) is reached, all the examined styryl cyanine derivatives **1a–f** spontaneously relax back to the more stable open form (Figures S34–S39), which is a characteristic of T‐type (metastable) photochromes.

We also followed the photoisomerization process of **1a–f** using solution NMR techniques. The ^1^H‐NMR of **1a** clearly shows a change in the chemical shift of all aromatic signals upon irradiation with UV light (Figure 2b). Moreover, upon irradiation with 365 nm light, it is also possible to discern two distinct phenomena: the appearance of a peak at ∼5.7 ppm associated with the proton of the newly formed secondary amino group (Figure 2b), and the disappearance of the peak belonging to the methylene protons at ∼1.49 ppm to form two singlets at ∼1.20 and ∼1.33 ppm (Figure S12), caused by the formation of a spiro carbon. The same phenomenon was also observed for **1b–f** (Figures S11–S22). ^19^F‐NMR experiments performed on fluorinated compounds **1d** and **1e** led to the same conclusions (Figures S25 and S26). These analyses further confirm the fact that molecules **1a–f** are able to ring close and that the changes in the UV–vis spectra (Figure 2a and Supplementary Figures S27–S32) are not caused by the E‐to‐Z isomerization but rather by the subsequent cyclization reaction. To consolidate this hypothesis, UV–vis absorbance kinetic experiments were performed on the nonclosable ortho‐methoxy (o‐OMe) analogue **1** **g**. Results show that irradiation of **1** **g** with UV light does trigger the E to Z double bond isomerization associated with a decrease in intensity of the main absorbance band (Supplementary Figure S33), but prolonged irradiation at 365 nm causes rising of two peaks: one at ∼250 nm and one at ∼400 nm that we associate with the photodegradation of the sample. ^1^H‐NMR experiments on **1** **g** show a clear, but incomplete, E to Z photoinduced isomerization of the double bond (Supplementary Figure S23) caused by the overlapping in the absorbance of the E and Z forms of **1** **g**.

**Figure 3:**
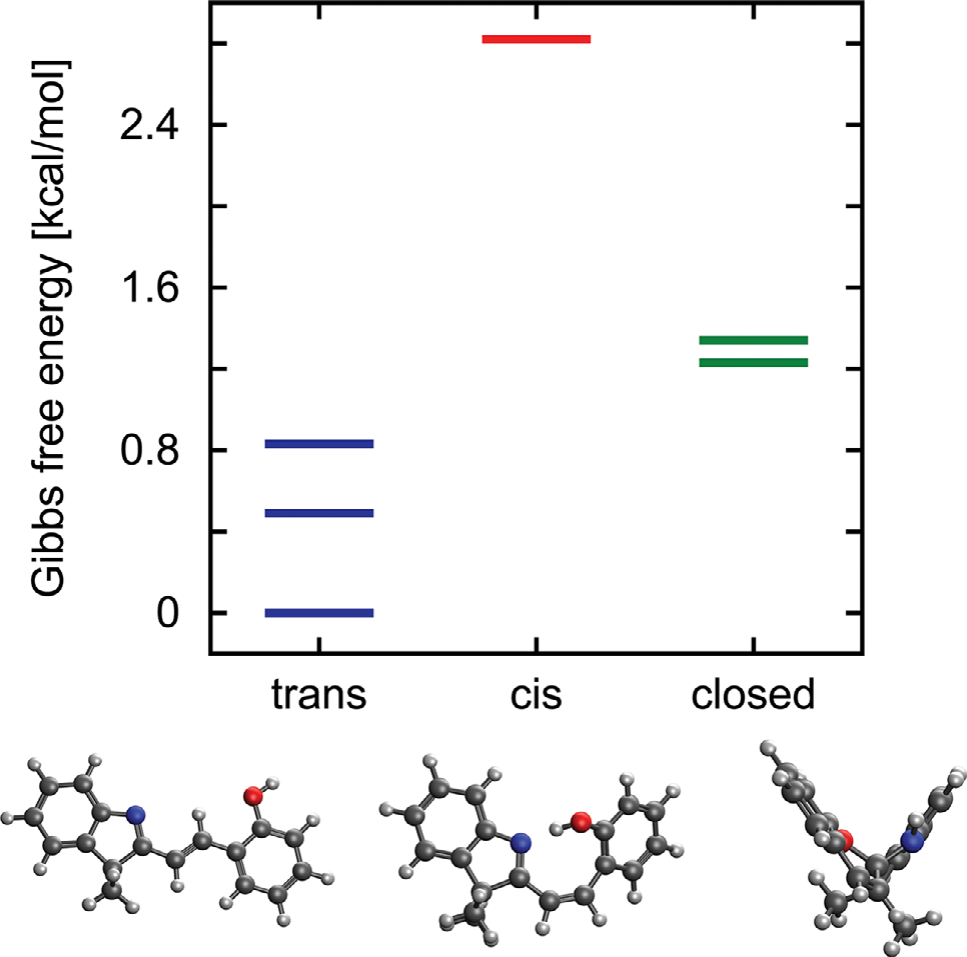
Gibbs free energies of the most stable trans, cis, and open forms of styryl cyanine 1a computed at the PW6B95/D3/cc‐pVTZ/C‐PCM level in acetonitrile. The sketches show the most stable structures in the trans, cis, and close conformations. Two trans isomers are found to be nearly degenerate at the energy of 0 kcal mol^−1^ in the scale of the figure.

To gain deeper insights into the structure of the styryl cyanines under investigation, we conducted density functional theory (DFT) calculations on molecule **1a**. Specifically, we optimized the structures of various trans and cis isomers of **1a**, along with its closed form, at the PW6B95/cc‐pVTZ level of theory, which is a similar level of theory previously validated for a merocyanine‐spiropyran switch.^[^ ^36^ ^]^ The geometry optimizations included D3 dispersion corrections and accounted for solvent effects (acetonitrile) via the conductor‐like polarizable continuum model (C‐PCM). The Gibbs free energy for each optimized structure was calculated, incorporating vibrational and rotational enthalpy and entropy contributions under the standard harmonic/rigid rotor approximation. The optimized structures are reported in the Supporting Information (Section 10 Supporting Information). The open‐form isomers primarily differ in the orientations of the 2‐hydroxybenzyl group and the hydroxyl group. For the trans form, four local minima were identified on the potential energy surface, including the global minimum depicted in Figure 3. The free energies of these trans structures are within 0.85 kcal mol^−1^, indicating that all conformers are likely to be significantly populated at room temperature.

For the cis conformers, five local minima were found, with free energies ranging from 2.8 to 10.2 kcal mol^−1^ above the global minimum. Although all these conformations could be formed through light irradiation, only the most stable cis isomer is expected to be present in a fraction >1% at room temperature, based on our calculations (the free energy of the other isomers is more than 6.5 kcal mol^−1^ higher). This isomer, shown in Figure 3, is stabilized by an intramolecular hydrogen bond between the hydroxy group and the lone pair of the nitrogen atom.

The PW6B95 calculations further indicate three possible closed isomers. Two of them, with energies approx. 1.2 kcal mol^−1^ above the most stable trans form, differing in the orientation of the chromene group (see Figure S92). The third isomer has an energy of 3.3 kcal mol^−1^ in the scale of Figure 3 and is obtained by an umbrella inversion of the amino group.

The fact that the closed forms are relatively stable aligns with experimental observations, discussed further below, which show that the closed form is present even in the dark when acetonitrile is used as solvent. A Boltzmann population analysis, based on the free energies of the optimized structures, predicts a 92:8 ratio of open to closed forms, with almost all the open molecules adopting a trans configuration. Notably, the highest conformational entropy contributes significantly to the higher abundance of open‐form structures.

One should point out that the precise energy differences across the different isomers (hence the Boltzmann populations) are hard to predict at the DFT level, given that the many different structures are found within few kcal mol^−1^. Nevertheless, as shown in section 10 of the Supporting Information, a qualitatively similar distribution of isomers is obtained by using the B3LYP functional instead of PW6B95.

To understand how the photophysical properties of styryl cyanines **1a–e** can be influenced, we proceeded to study how electron‐donating and electron‐withdrawing groups placed in position 4 of the indole half and position 5 of the styryl ring can influence the behavior of these molecules. When dissolved in acetonitrile and stored in the dark, the synthesized compounds present themselves as a mixture of open and closed forms. Remarkably, styryl cyanines **1a–e** all convert to their closed form quantitatively or almost quantitatively when irradiated with 365 nm light, making them extremely efficient photoswitches (Table 1). The photoisomerization quantum yield (QY) has also been determined using the total absorbance method reported elsewhere.^[^ ^37^ ^]^ The highest value of QY was found to be 46% for **1a**, and it was consistently above 20% for all the studied compounds (Table 1); no trend related to the effect of substituents on the ring was observed in this case. On the other hand, the half‐life time in the dark of the closed isomer was dictated by the substituents on the ring. In particular, as shown in Table 1, fluorine groups significantly slow down the ring‐opening reaction in the dark, extending the half‐life to more than double that of photoswitches bearing methoxy groups. This behavior aligns with recent findings for merocyanine photoacids, which share a similar conjugated structure with **1a–f**.^[^ ^38^ ^]^ We speculate that the nature of the substituent influences the energy barrier of the back relaxation kinetic in polar aprotic solvents such as acetonitrile. Further mechanistic studies are needed to fully understand the energy levels of closed and open styryl cyanines **1a–f**, as well as the energy barrier between them.

**Table 1:** Summary of the properties of photoswitches **1a–f**. Molar extinction coefficient (*ε*) calculated at 20 °C in CH_3_CN at maxima; ratio between open and closed form in the dark (dark state); ratio at PSS obtained upon irradiation at 365 nm LED (PSS_365_); photoisomerization quantum yields for photoswitches (QY); thermal half‐life of the closed forms (t_1/2_) in the dark in acetonitrile and methanol; and p*K*_a_ *^dark/hν^* of **1a–f** in water:DMSO 99:1. No change in the NMR spectra of **1f** was recorded in D_2_O upon irradiation with 365 nm light.

Compound	ɛ (M^−1^*cm^−1^) in ACN	Dark state (open:closed) (^1^H‐NMR) in ACN_d3_	PSS_365_ (open:closed) (^1^H‐NMR) in ACN_d3_	QY [open →closed] in ACN	t_1/2_ (s) in ACN, 20 °C	t_1/2_ (s) in MeOH, 20 °C	p*K*_a_ *^dark^*	p*K*_a_ *^hν^*
1a	18 677 ± 40^††^	95:5^*^	0:100^*^	46%	3293	106	4.3	3.2
1b	22 537 ± 87	99:1^†^	22:78^†^	28%	2407	358	4.6	4.0
1c	20 586 ± 50^††^	94:6^†^	4:96^†^	30%	2646	7	4.3	4.1
1d	25 744 ± 13^††^	97:3^*^	1:99^*^	35%	4012	345	4.3	3.1
1e	25 454 ± 81^††^	94:6^*^	0:100^*^	23%	5488	341	4.3	3.2
1f_DMSO_	–	92:8^[Link]^, ^[Link]^	10:90^[Link]^, ^[Link]^	–	–	–	–	–
1f_water_	21 678 ± 58^‡^	100:0^[Link]^, ^[Link]^	–	33%^‡^	–	154^‡^	3.9	2.2

^*^Ratio calculated using the signal of the two geminal methyl groups.

^†^Ratio calculated using the signal of the methoxy group.

^‡^Milliq Water used as solvent.

^§^DMSO_d6_ used as solvent.

^#^D_2_O used as solvent.

^††^Corrected based on the ratio between open and closed form derived from NMR experiments.

Having explored the effect of substituents, we next focused on the impact of the medium. As shown in Table 2, we found that the solvent has a clear impact on the ring‐opening kinetics of compound **1a**. Since **1a** is soluble in both polar and apolar solvents, we also had the opportunity to investigate the effect of the latter on the ring‐closing and ring‐opening kinetics. We found that, even though the photoinduced isomerization is relatively comparable for all the solvents (Supporting Information Section 5.2.6), the ring‐opening process is extremely dependent on the solvent polarity, being slower in toluene, and fast in polar protic solvents such as water or methanol. These findings can be explained using the reported hydrogen‐bond donor (HBD) acidity (Taft–Kamlet α‐scale) of the utilized solvents.^[^ ^39^ ^]^ Such a big difference in thermal half‐life has to be associated with the proton transfer from the secondary amine (in the closed form) to the phenol group since the back relaxation from the closed form to the open conjugated form is a thermodynamic process. We hypothesize that solvents like water and methanol trigger a Grotthus‐like proton transfer mechanism which speeds up the back‐relaxation process, similar to what was previously reported for other phenol‐bearing photoswitches.^[^ ^40^ ^]^

**Table 2:** Half‐life time of closed **1a** in different solvents.

Solvent	t_1/2_ (s)
Toluene	9503
DMSO	6542
Methanol	106
Water + 10% DMSO	117

**Figure 4:**
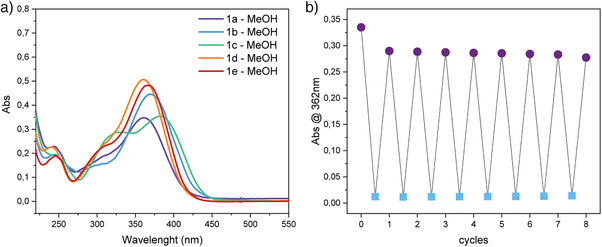
a) Overlap of UV–vis absorbance spectra of 1a–e in methanol (0.02 mM, 20 °C). b) Plot showing the fatigue resistance of 1a in methanol (0.02 mM, 20 °C) (irradiation with 365 nm LED).

Given the faster back relaxation kinetic of **1a** in methanol, we decided to also measure the absorbance spectrum of **1b–e** in the same solvent (Figure 4a) and measure its effect on the closed form's half‐life time (Table 1). Overall, the half‐life time of the closed forms of **1b–e** follows the same trend already observed for the closed form of **1a**, and it is generally affected by the presence of methanol when it is used as a solvent in such a way that it is one order of magnitude smaller for all the examined styryl cyanine, exception made for molecule **1c**, for which the half‐life is two orders of magnitude smaller compared to acetonitrile. Being the thermal half‐life time greatly influenced by the nature of the solvent, we also opted to use methanol as a medium to carry out fatigue resistance experiments (Figures 4b and S52). All the photoswitches **1a–f** exhibit superb fatigue resistance over several cycles, without the need to degas the solvent.

Since light‐induced cyclization is also associated with a change in the nature of the amino group of **1a–e**, we investigated the change in their p*K*_a_ upon light irradiation. This phenomenon has been already reported in the literature for other photoresponsive molecules,^[^ ^41^, ^42^ ^]^ and it is recognized that, apart from being key in many biological processes, is a powerful tool for potential applications in catalysis^[^ ^43^ ^]^ or self‐assembly.^[^ ^44^ ^]^ As shown in Table 1, the p*K*_a_ of the open forms (p*K*_a_ *^dark^*) is consistently higher than that of the closed forms (p*K*_a_ *^hν^*) for all the studied compounds. Moreover, for compounds **1b** and **1c**, bearing methoxy groups, the change in p*K*_a_ generated upon light irradiation is considerably smaller than for compounds **1d** and **1e**, bearing fluorine groups. This difference can be easily associated with the nature of the substituents, where for **1d–e** the electron‐withdrawing effect of the fluorine favors a higher acidity of the closed isomers, contrarily to the effect of the electron‐donating groups for **1b–c**.

Among the synthesized molecules, compound **1f** shows peculiar characteristics. Compared to **1a–e**, which are soluble in classic organic solvents ranging from acetonitrile to toluene but not in pure water, compound **1f** is fully soluble and fully switchable in water, due to the presence of a sulfonic acid potassium salt group in position 4 of the indolenine part. Similar to what is observed for the other compounds, the half‐life of closed **1f** in water is extremely small (Table 1). This opens the opportunity of using **1f** in out‐of‐equilibrium^[^ ^45^ ^]^ and dissipative water‐based systems. Moreover, irradiation of **1f** triggers the photo reactions that generate its respective closed indole‐like form, which compared to the closed forms of **1a–e** also has a lower value of pK^hν^ (Table 1) and generates a higher Δp*K*_a_ value between the open and closed forms, being 1.7 p*K*_a_ units, thanks to the electron‐withdrawing effect coming from the presence of a sulfonate group.

**Figure 5:**
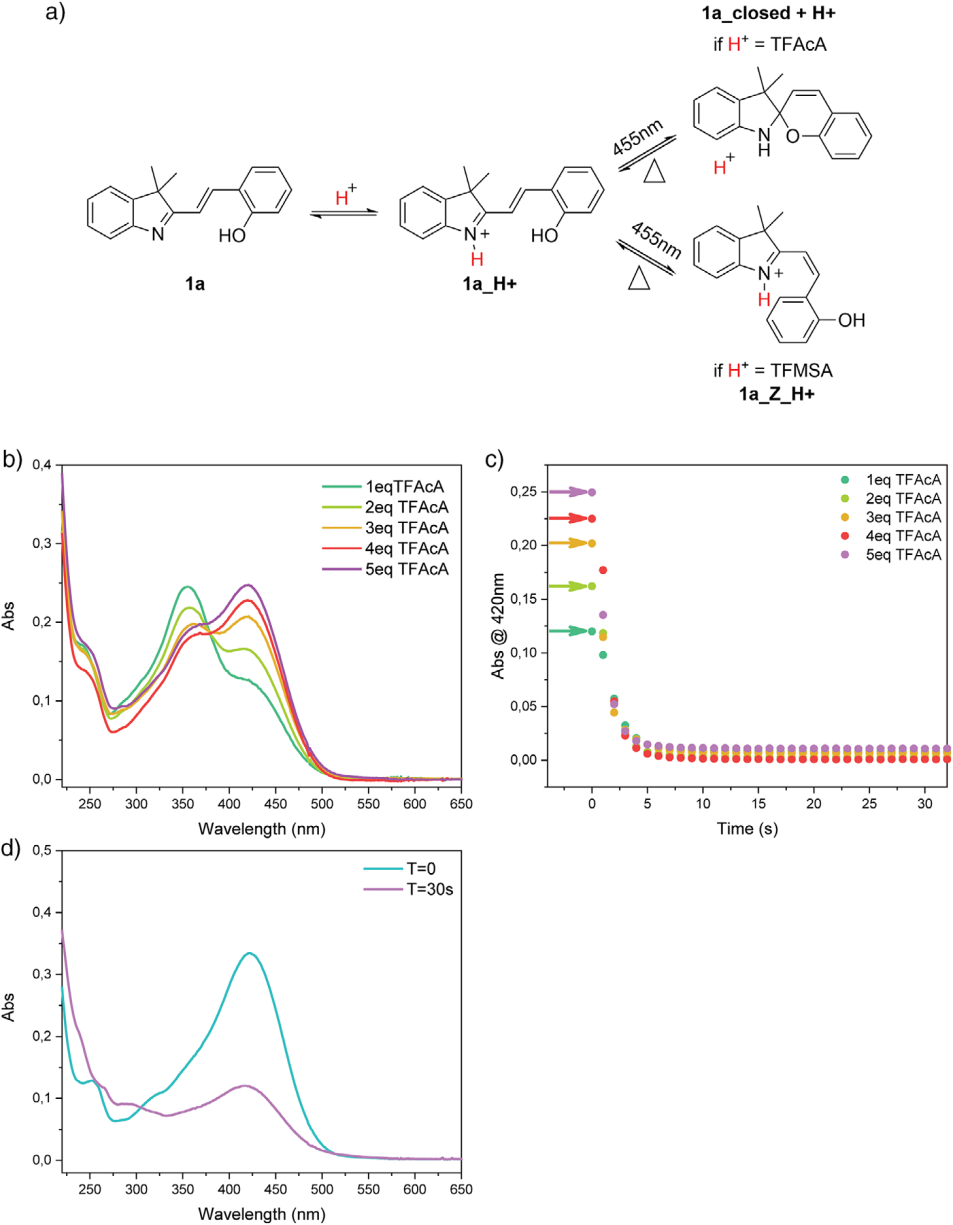
a) Protonation of 1a and different photoisomerization pathways depending on the type of acid utilized. b) Overlap of UV–vis absorbance spectra of 1a (0.02 mM, 20 °C) with increasing amounts of trifluoroacetic acid (TFAcA) in CH3CN. c) The change in absorbance of 1a (0.02 mM, 20 °C, and CH_3_CN) plus increasing amount of TFAcA at 420 nm over time during irradiation at 455 nm d) UV–vis absorbance spectra of 1a (0.02 mM, 20 °C, and CH_3_CN) with 1 equiv of trifluoromethane sulfonic acid in the dark (*T* = 0 s) and after 30 s of irradiation with a 455 nm LED.

Compared to SP, styryl cyanine molecules **1a–f** absorb light in a similar region, i.e., the absorption maxima is around 365 nm (Figure 1a and Section 5 in the Supporting Information). Although this is a commonly utilized wavelength for a large variety of photoresponsive molecules, efforts have been made in the past years to shift the absorbance of at least one of the two forms of photoswitches in the visible light range using push‐pull systems or extended conjugation, triplet sensitization, multiphoton absorption, and more.^[^ ^46^ ^]^ To shift the absorbance of styryl cyanine **1a** to longer wavelengths, we decided to use the effect of acids. Contrary to what happens to spiropyran‐like photoswitches,^[^ ^47^ ^]^ protonation of the nitrogen in **1a** does not alter the molecule's behavior under light irradiation. As shown in Figure 5a–c, styryl cyanine **1a** can still undergo a photoinduced ring‐closing reaction when protonated with trifluoroacetic acid (TFAcA). We also show in Figure 5b that an increasing amount of TFAcA translates into an increase in the intensity of the absorption band of **1a_H +** centred at ∼440 nm. Irradiation of **1a_H +** still results in the disappearance of the bands associated with the open form (Figures 5c and S83), i.e., the acidochromic properties of styryl cyanine **1a** can be used to red‐shift its absorbance in the visible light range, without inhibiting the light‐induced ring‐closing reaction.

On the contrary, when trifluoromethanesulfonic acid (TMFSA) is used, the behavior of **1a** is altered, and the ring‐closing reaction is forbidden. This is in agreement with what was previously reported by Kortekaas et al. on spiropyrans,^[^ ^47^ ^]^ for which the protonation by acids with a too low p*K*_a_ results in the loss of the ability of the molecule to ring close upon irradiation with light and leads instead to the formation of the open Z isomer (**1a_Z_H + **in Figure 5a). Formation of the Z isomer of **1a** upon irradiation of a solution containing 1 equiv TMFSA can be followed using UV–vis absorbance spectroscopy. The main peak does not completely disappear upon irradiation with visible light, but its intensity is only decreased (Figures 5d and S84).

In recent years, photoswitches capable of interacting with protons, especially spiropyran‐like molecules, have gained significant attention in the emerging field of light‐controlled proton and ion transport. Specifically, different strategies have been recently developed, mainly utilizing metal‐organic frameworks (MOFs) functionalized^[^ ^48^ ^]^ or blended^[^ ^49^ ^]^ with photoswitches. Alternatively, the use of excited‐state photoacids as an additive^[^ ^50^ ^]^ or as side pending groups^[^ ^51^ ^]^ in already proton‐conductive polymers or melting coordination polymer crystals^[^ ^52^ ^]^ has also been explored. The increased interest in light‐controlled proton and ion transport is connected to the fact that it not only mimics natural processes,^[^ ^53^, ^54^ ^]^ but can also be used in light‐responsive devices.^[^ ^49^, ^55^, ^56^, ^57^ ^]^

**Figure 6:**
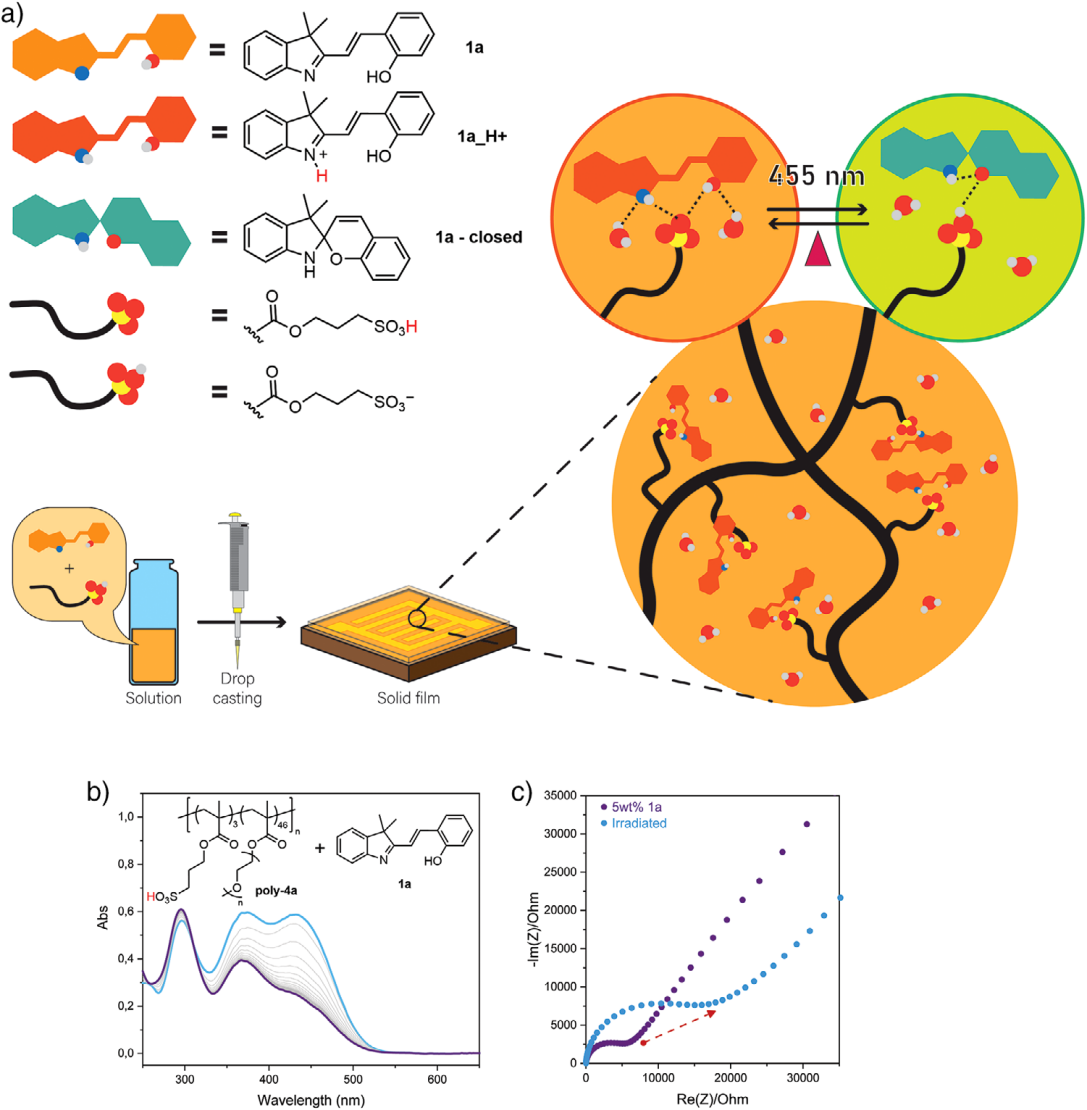
a) Schematic representation of the preparation of styryl cyanine‐based light‐responsive polymeric films. b) Solid state UV–vis absorbance kinetic spectra for the photoisomerization of poly‐4a + 5wt% 1a. c) Change in the impedance spectra of poly‐4a + 5wt% 1a upon irradiation with 455 nm light (purple = dark, cyan = irradiated).

Because of its affinity with acids, we used **1a** as a smart dopant inside **poly‐4a** (Figure 6b), an acid‐functionalized polymer. As illustrated in Figure 6a, the addition of 5 wt% of **1a** to a solution of **poly‐4a** results in its protonation, to form **1a_H + **. Indeed, the films cast from this solution present the characteristic absorbance peak at ∼450 nm (Figure 5b) which is absent in the absorbance spectrum of pure **poly‐4a** (Figure S5.6.1.1), and it is associated with the formation of **1a_H + **. The polymer‐photoswitch blends show excellent film‐formation ability and can be cast on top of interdigitated gold electrodes supported on Si/SiO_2_. The films exhibit remarkable reversible responsiveness to blue light (Figure 6a,b and Section 9.2 Supporting Information) thanks to the negative photochromic nature of **1a_H+**, and its elevated switching efficiency.

When placed in a controlled humidity atmosphere, the presence of **1a** enables the photocontrolled modulation of the proton transport inside the polymeric material, as proved by the change in the impedance spectra in the dark and upon irradiation (Figure 6c). When **1a_H +** is irradiated and the closed **1a** isomer is produced (Figure 5a), thanks to the large structural changes of the photoswitch, the hydrogen‐bonding network inside the material is perturbed.^[^ ^48^, ^56^ ^]^ This generates a distortion of the network of water molecules, which is the key feature to achieving efficient movement of protons in ion‐conducting polymers,^[^ ^58^, ^59^ ^]^ increasing resistance toward proton transport. As illustrated in Figure 5a, the light‐induced ring closing of **1a_H +** destroys the charge‐assisted N^+^‐H─^−^O‐S hydrogen bond network necessary to achieve efficient proton movement inside the material. Moreover, after irradiation with visible light, the system changes from **1a_H+**, which is formally charged (+1), to the closed neutral form of **1a**, becoming in this way more hydrophobic, and less prone to proton conduction. This demonstrates that the proton transport in polymeric materials can be easily controlled by light (Figures 6c, and S91) through simple blending of **1a** with an acidic polymer, thanks to the difference in affinity of its open and closed form with acidic groups and its light‐induced change in hydrogen bonding and hydrophilicity. Moreover, an increase of the resistance in the dark from 4035 Ohm to more than triple the value has been achieved with this simple strategy (Figure 6c).

## Conclusion

In this study, we synthesized a series of styryl cyanine photoswitches through a straightforward process and demonstrated their unique and efficient switching properties. Notably, introducing an ortho‐phenol group on the styryl cyanine backbone enabled unprecedented light‐induced formation of a closed spiro form, which is blue‐shifted relative to the open form. These photoswitches exhibit excellent fatigue resistance, rapid light‐triggered ring‐closing kinetics, and most importantly, high switching efficiency, with some of them reaching quantitative conversion to their metastable form. Additionally, we demonstrated that the kinetics of back‐relaxation can be finely tuned from hours to seconds by varying the substituents, solvents, or both. We also established that the p*K*_a_ of these molecules can be modulated by light and influenced by the nature of the attached substituents. Furthermore, we developed a strategy to redshift their absorbance into the visible range by leveraging the affinity of these molecules for acidic systems. Using this approach, we demonstrated that they can function as a smart dopant for sulfonic acid‐decorated polymers, yielding a visible light‐responsive material capable of modulating proton transport upon blue light irradiation. These light‐responsive ion‐conducting materials offer promising applications in organic electronics and hold significant potential for future use in sensors, flexible iontronic devices, and neurotrophic interfaces.

## Conflict of Interests

The authors declare no conflict of interest.

## Supporting information



Supporting Information

## Data Availability

The data that support the findings of this study are available from the corresponding author upon reasonable request.
