# Supplementary material for: Harnessing Negative Photochromism in Styryl Cyanines for Light‐Modulated Proton Transport
Source: Angew Chem Int Ed Engl. 2025 May 16;64(28):e202506532. doi: 10.1002/anie.202506532 (PMC12232897; doi:10.1002/anie.202506532)
Supplement: Supplementary file 1 — Supporting Information [file ANIE-64-e202506532-s001.docx]

**Supplementary information**

**Harnessing Negative Photochromism in Styryl Cyanines for Light-Modulated Proton Transport**

Gianni Pacella^1^, Maria Nabatova^1^, Yuxuan Zhang^1^, David Picconi^2^, Roza Weber^3^, Shirin Faraji^1,2^, and Giuseppe Portale*^1^

^1^Macromolecular Chemistry and New Polymeric Materials, Zernike Institute for Advanced Materials, Faculty of Mathematics and Natural Sciences, University of Groningen, Groningen, the Netherlands

^2^Institute of Theoretical and Computational Chemistry, Heinrich Heine, University Düsseldorf, Düsseldorf, Germany

^3^Stratingh Institute for Chemistry, Center for Systems Chemistry and Zernike Institute for Advanced Materials, Faculty of Mathematics and Natural Sciences, University of Groningen, Groningen, the Netherlands

*Corresponding author: g.portale@rug.nl

**Table of Contents**

-1. General Information

-2. Experimental procedures and analytical data

-2.1. Synthesis of precursors

-2.2. Synthesis of Styryl Cyanines

-2.3. Synthesis of polymers

-3. NMR spectra of Styryl Cyanines

-4. In situ irradiation NMR spectra

-4.1. General procedure

-4.2. ^1^H-NMR with in situ irradiation

-4.3. ^19^F-NMR with in situ irradiation

-5. UV-Vis studies

-5.1. General procedure

-5.2. Irradiation experiments

-5.2.1. Photoisomerization kinetics

-5.2.2. Back relaxation kinetics

-5.2.3. Photoisomerization kinetics in Methanol

-5.2.4. Back relaxation kinetics in Methanol

-5.2.5. Comparison of dark spectra in acetonitrile and methanol

-5.2.6. Photoisomerization kinetics and Back relaxation kinetics in solvents different from Acetonitrile

-5.2.7. Fatigue resistance experiments

-5.3. pKa determination experiments

-5.3.1. pKa titration of the dark state

-5.3.2. pKa titration of the photostationary state

-5.4. Photoisomerization quantum yield determination

-5.4.1. Photon Flux

-5.4.2. Photoisomerizagtion Quantum Yield of 1a-f

-5.5. Molar extinction coefficients determination

-5.6. UV-Vis studies on polymeric systems

-6. Acidochromism

-6.1. Acidochromism in the presence of trifluoroacetic acid

-6.2. Acidochromism in the presence of trifluoromethanesulfonic acid

-7. Thermal analysis

-7.1. Thermogravimetric analysis (TGA)

-7.2. Differential Scanning Calorimetry (DSC)

-8. Gel Permeation Chromatography (GPC)

-9. Electrochemical impedance spectroscopy

-9.1. General procedures

-9.2. EIS of polymeric films with 1a

-10. DFT calculations

-11. Bibliography

**1. General Information**

All reagents and solvents have been purchased from Sigma Aldrich, TCI Europe, Acros Organics, BOOM Chemicals, and were used without further purifications unless specified. Dry solvents have been obtained from an MBraun SPS-800 solvent purification system. All the reactions have been carried out under argon atmosphere. Standard NMR spectra (^1^H, ^13^C, and ^19^F) have been recorded on a Varian Mercury-Plus (400 MHz) spectrometer at 298K. ^1^H-NMR with in situ irradiation has been performed on a  Varian Unity Plus (500 MHz) using a custom-made optic fibre for the irradiation, modified to fit into the NMR tube, as previously described in the literature.^[1]^ Chemical shifts are in parts per million (ppm) relative to TMS. For the calibration of the chemical shift, the residual solvent resonance has been used as the internal standard.

Flash column chromatography was carried out using silica gel Davisil LC60A (Merck type 9385, 230400 mesh) using the specified eluents.

High-resolution spectroscopy (HRMS) was performed on an LTQ Orbitrap XL spectrometer with electrospray ionization (ESI) as the ionization technique.

UV-Vis absorption spectra were recorded on an Agilent 8453UV-Vis Diode Array System, equipped with a Quantum Northwest Peltier controller in 10 mm quartz cuvettes. Irradiation experiments were performed using LEDs from Thorlabs Incorporated (365 nm, and 455 nm; 1 A) (models M365FP1 - 365 nm and M455F3 - 455 nm).

EIS measurements have been performed similarly to what has already been previously reported from our group.^[2]^ EIS measurements were performed using an SP-300 Biologic impedance spectrometer. The oscillating amplitude was fixed at 100 mV, and impedance spectra were collected by scanning the frequency from 7 MHz to 100 mHz in the logarithmic scale, with 10 points per decade. The samples were casted on gold interdigitated electrodes (IDEs) deposited on silicon substrates with 1000 nm SiO2 insulating top layer and placed in a humidified chamber with relative humidity of 100%. The impedance data were fitted using the EC-Lab Zfit software.

**2. Experimental procedures and analytical data**

**2.1. Synthesis of precursors**

5-methoxy-2,3,3-trimethyl-3H-indole (**2a**): indolenine 2a has been prepared from a modified literature procedure.^[3]^ A solution of 4-methoxyphenyl hydrazine (3.00g, 17.18 mmol), methyl isopropyl ketone (2 ml, 18.90 mmol), and p-toluene sulfonic acid dihydrate (0.33g, 1.72 mmol) in absolute ethanol (60 ml) was heated to reflux under inert atmosphere for 16-24h (reaction checked by TLC 1:1 EtOAc : Hex). After cooling down to room temperature, the solvent was removed under reduced pressure. The crude has been dissolved in 100 ml of dichloromethane and extracted using water (3 x 100 ml) and NaHCO_3_ sat. (1 x 100 ml). The organics were dried over Na_2_SO_4_ and the solvent was removed under reduced pressure to give 2a (3.24 g, 99%) as a dark red solid.

^1^H NMR (400 MHz, DMSO-d6) δ 7.29 (d, *J* = 8.4 Hz, 1H), 7.01 (d, *J* = 2.5 Hz, 1H), 6.78 (dd, *J* = 8.4, 2.6 Hz, 1H), 3.74 (s, 3H), 2.13 (s, 3H), 1.19 (s, 6H).

^13^C NMR (101 MHz, DMSO-d6) δ 185.68, 157.82, 147.98, 147.57, 119.91, 112.62, 108.61, 55.87, 53.75, 23.09, 15.34.

MS-ESI (m/z) : [M+H]^+^ calc. for C_12_H_15_NO 190.1226; found [M+H]^+^ 190.1222

5-fluoro-2,3,3-trimethyl-3H-indole (**2b**): A solution of 4-fluorophenyl hydrazine hydrochloride (1.00g, 6.15 mmol) and p-toluene sulfonic acid (0.11g, 0.61 mmol) are placed under inert atmosphere. Subsequently, 20 ml of absolute ethanol and methyl isopropyl ketone (1 ml, 9.22 mmol) are added. The solution was heated to reflux for 16-24h, then the solvent was removed under reduced pressure, and the crude was dissolved in 100 ml of dichloromethane, extracted with 100 ml of NaHCO_3_ sat., and dried over Na_2_SO_4_. The organics were removed under reduced pressure to yield 2b as a yellow solid (0.94g, 86%).

^1^H NMR (400 MHz, DMSO-d6) δ 7.38 (dd, *J* = 8.4, 4.8 Hz, 1H), 7.30 (dd, *J* = 8.4, 2.7 Hz, 1H), 7.04 (m, *J* = 9.6, 8.4, 2.6 Hz, 1H), 2.16 (s, 3H), 1.22 (s, 6H).

^13^C NMR (101 MHz, DMSO-d6) δ 188.26, 162.01, 159.62, 150.17, 148.71, 148.62, 120.52, 120.43, 114.28, 114.04, 110.06, 109.82, 54.27, 22.73, 15.44.

^19^F NMR (376 MHz, DMSO-d6) δ -118.15 (m, *J* = 9.1, 4.7 Hz).

MS-ESI (m/z) : [M+H]^+^ calc. for C_11_H_12_FN 178.1027; found [M+H]^+^ 178.1024

potassium 2,3,3-trimethyl-3H-indole-5-sulfonate (2c): indolenine 2c has been prepared from literature procedure.^[4]^

To a 3-necked round bottomed flask (50 mL) equipped with a condenser the 4- hydrazinylbenzenesulfonic acid hemihydrate (6.0187 g, 31.98 mmol, 1 eq) was added. The flask was then put under an inert Argon atmosphere. After the addition of 3-methyl-2-butanone (6.8 g, 8.5 mL, 78.95 mmol, 3 eq) and 16.5 mL glacial acetic acid, the reaction mixture was refluxed for 23h. Afterwards, the mixture was cooled down in an ice bath and filtered over a por. 3 glass filter. The solids were washed with an excess of acetone and twice with 50 mL ethanol. The pink solid was then put into the vacuum oven to dry. These solids were then added to a flask to which 80 mL of MeOH and 20 mL of KOH iPrOH was also added under stirring. A glittery yellow precipitate formed which was filtered over a glass filter por. 3, and washed twice with 50 mL EtOH. Finally, the yellow solid was dried in the vacuum oven (2.42 g, 54 % yield).

^1^H-NMR (400 MHz, DMSO-d6) δ 7.63 (d, J = 1.1 Hz, 1H), 7.55 (dd, J = 7.9 Hz, 1.6 Hz, 1H), 7.34 (d, J = 7.9 Hz, 1H), 2.21 (s, 3H), 1.24 (s, 6H).

^13^C NMR (101 MHz, DMSO-d6) δ 188.84, 153.61, 145.19, 145.13, 125.11, 119.17, 118.13, 53.24, 22.51, 15.16.

MS-ESI (m/z) : [M+H]^+^ calc. for C_11_H_12_KNO_3_S 277.02; found [M+H]^+^ 278.02.

**2.2. Synthesis of Styryl Cyanines**

(E)-2-(2-(3,3-dimethyl-3H-indol-2-yl)vinyl)phenol (**1a**): A solution of 2,3,3-trimethyl indolenine (2ml, 12.43 mmol), salicylaldehyde (2.66 ml, 24.86 mmol), and piperidine (0.24 ml, 2.49 mmol) in absolute ethanol (10 ml) is heated to reflux for 16-24h. The solvent is then removed at reduced pressure, and the resulting crude oil is dissolved in 10 ml of diethyl ether and precipitated, drop by drop, in 300 ml of pentane under vigorous stirring. The resulting yellow solid is filtered on a glass filter por.3, washed with pentane (3 x 25 ml), and dried in a vacuum oven at 75°C to yield 1a as a yellow solid (1.68g, 51% yield).

In some cases flash chromatography might be required, and has to be performed using a 70:30 EtOAc:Hex eluent (R_f_= 0.33).

^1^H NMR (400 MHz, DMSO-d6) δ 7.99 (d, *J* = 16.5 Hz, 1H), 7.73 (dd, *J* = 7.9, 1.7 Hz, 1H), 7.51 – 7.47 (m, 1H), 7.44 (dd, *J* = 7.3, 1.2 Hz, 1H), 7.29 (td, *J* = 7.5, 1.3 Hz, 1H), 7.23 – 7.15 (m, 3H), 6.90 (dd, *J* = 8.2, 1.1 Hz, 1H), 6.84 (td, *J* = 7.5, 1.2 Hz, 1H), 1.37 (s, 6H).

^13^C NMR (101 MHz, DMSO-d6) δ 184.01, 156.69, 154.32, 147.03, 133.32, 130.98, 128.04, 127.96, 125.60, 123.04, 121.93, 120.32, 119.82, 119.15, 116.54, 52.60, 23.57.

MS-ESI (m/z) : [M+H]^+^ calc. for C_18_H_17_NO 263,1383; found [M+H]^+^ 263,1379

(E)-2-(2-(5-methoxy-3,3-dimethyl-3H-indol-2-yl)vinyl)phenol (**1b**): A solution of 2a (0.55 g, 2.90 mmol), salicylaldehyde (0.63 ml, 5.80 mmol), and piperidine (0.06 ml, 0.58 mmol) in absolute ethanol (8 ml) is heated to reflux for 16-24h. The solvent is then removed at reduced pressure, and 2 ml of ethyl acetate are added to the crude which is then precipitated, drop by drop, in 300 ml of pentane under vigorous stirring. The resulting yellow solid is filtered on a glass filter por.3, washed with pentane (3 x 25 ml), and dried in a vacuum oven at 75°C to yield 1b as a yellow solid (0.20 g, 24%).

^1^H NMR (400 MHz, DMSO-d6) δ 7.88 (d, *J* = 16.6 Hz, 1H), 7.69 (dd, *J* = 7.8, 1.7 Hz, 1H), 7.39 (d, *J* = 8.4 Hz, 1H), 7.20 – 7.11 (m, 2H), 7.06 (d, *J* = 2.6 Hz, 1H), 6.89 (dd, *J* = 8.2, 1.2 Hz, 1H), 6.86 – 6.80 (m, 2H), 3.77 (s, 3H), 1.36 (s, 6H).

^13^C NMR (101 MHz, DMSO-d6) δ 181.94, 158.25, 156.43, 148.88, 147.88, 132.01, 130.67, 127.73, 123.22, 120.75, 119.83, 119.53, 116.48, 113.19, 108.32, 55.94, 52.79, 23.76.

MS-ESI (m/z) : [M+H]^+^ calc. for C_19_H_19_NO_2_ 293,1484; found [M+H]^+^ 293,1489

(E)-2-(2-(3,3-dimethyl-3H-indol-2-yl)vinyl)-4-methoxyphenol (**1c**): A solution of 2,3,3-trimethyl indolenine (0.8 ml, 4.97 mmol), 5-methoxy salicylaldehyde (1.24 ml, 9.95 mmol), and piperidine (0.1 ml, 0.99 mmol) in absolute ethanol (13 ml) is heated to reflux for 16-24h. The solvent is then removed at reduced pressure, and 2 ml of diethyl ether are added to the crude which is then precipitated, drop by drop, in 300 ml of pentane under vigorous stirring. The resulting yellow solid is filtered on a glass filter por.3, washed with pentane (3 x 25 ml), and dried in a vacuum oven at 75°C to yield 1b as a yellow solid (0.53 g, 36%).

^1^H NMR (400 MHz, DMSO-d6) δ 9.62 (s, 1H), 7.98 (d, *J* = 16.6 Hz, 1H), 7.49 (d, *J* = 7.6 Hz, 1H), 7.42 (d, *J* = 7.3 Hz, 1H), 7.35 – 7.12 (m, 4H), 6.93 – 6.75 (m, 2H), 3.74 (s, 3H), 1.37 (s, 6H).

^13^C NMR (101 MHz, DMSO-d6) δ 184.04, 154.32, 152.84, 150.80, 147.08, 133.19, 128.04, 125.61, 123.31, 121.92, 120.32, 119.38, 117.71, 117.39, 111.50, 56.00, 52.63, 23.54.

MS-ESI (m/z) : [M+H]^+^ calc. for C_19_H_19_NO_2_ 293,1484; found [M+H]^+^ 293,1489

(E)-2-(2-(5-fluoro-3,3-dimethyl-3H-indol-2-yl)vinyl)phenol (**1d**): A solution of 2b (0.80 g, 4.51 mmol), salicylaldehyde (0.96 ml, 9.03 mmol), and piperidine (0.09 ml, 0.90 mmol) in absolute ethanol (12.5 ml) is heated to reflux for 16-24h. The solvent is then removed at reduced pressure, and 2 ml of diethyl ether are added to the crude which is then precipitated, drop by drop, in 300 ml of pentane under vigorous stirring. The resulting yellow solid is filtered on a glass filter por.3, washed with pentane (3 x 25 ml), and dried in a vacuum oven at 75°C to yield 1b as a beige solid (0.37 g, 29%).

^1^H NMR (400 MHz, DMSO-d6) δ 10.06 (s, 1H), 7.97 (d, *J* = 16.5 Hz, 1H), 7.72 (dd, *J* = 7.8, 1.7 Hz, 1H), 7.48 (dd, *J* = 8.4, 4.8 Hz, 1H), 7.36 (dd, *J* = 8.4, 2.6 Hz, 1H), 7.21 – 7.14 (m, 2H), 7.10 (ddd, *J* = 9.6, 8.4, 2.7 Hz, 1H), 6.90 (dd, *J* = 8.2, 1.2 Hz, 1H), 6.84 (td, *J* = 7.5, 1.2 Hz, 1H), 1.37 (s, 6H).

^13^C NMR (101 MHz, DMSO-d6) δ 184.24, 184.20, 162.20, 159.80, 156.62, 150.51, 150.49, 149.38, 149.29, 133.21, 131.01, 127.98, 122.97, 121.20, 121.11, 119.85, 118.95, 116.52, 114.71, 114.47, 109.97, 109.73, 53.33, 53.31, 23.33.

^19^F NMR (376 MHz, DMSO-d6) δ -117.15 (td, *J* = 9.0, 4.7 Hz), -126.28 (td, *J* = 9.1, 4.5 Hz).

MS-ESI (m/z) : [M+H]^+^ calc. for C_18_H_16_FNO 281,1288; found [M+H]^+^ 281,1289

(E)-2-(2-(3,3-dimethyl-3H-indol-2-yl)vinyl)-4-fluorophenol (**1e**): A solution of 2,3,3-trimethyl indolenine (0.8 ml, 4.97 mmol), 5-fluoro salicylaldehyde (1.39 g, 9.95 mmol), and piperidine (0.1 ml, 0.99 mmol) in absolute ethanol (13 ml) is heated to reflux for 16-24h. The solvent is then removed at reduced pressure, and 2 ml of diethyl ether are added to the crude which is then precipitated, drop by drop, in 300 ml of pentane under vigorous stirring. The resulting yellow solid is filtered on a glass filter por.3, washed with pentane (3 x 25 ml), and dried in a vacuum oven at 75°C to yield 1b as a yellow solid (1.12 g, 80%).

^1^H NMR (400 MHz, DMSO-d6) δ 10.06 (s, 1H), 7.96 (dd, *J* = 16.4, 1.7 Hz, 1H), 7.65 (dd, *J* = 10.0, 3.1 Hz, 1H), 7.50 (d, *J* = 7.5 Hz, 1H), 7.43 (dd, *J* = 7.5, 1.3 Hz, 1H), 7.34 – 7.23 (m, 2H), 7.19 (td, *J* = 7.4, 1.1 Hz, 1H), 7.02 (td, *J* = 8.5, 3.1 Hz, 1H), 6.88 (dd, *J* = 8.9, 4.9 Hz, 1H), 1.36 (s, 6H).

^13^C NMR (101 MHz, DMSO-d6) δ 183.83, 157.34, 155.02, 154.25, 152.91, 152.90, 147.03, 131.95, 131.93, 128.06, 125.76, 124.13, 124.05, 121.97, 120.45, 120.29, 117.55, 117.51, 117.47, 117.28, 113.30, 113.08, 52.72, 23.32.

^19^F NMR (376 MHz, DMSO-d6) δ -125.08 (q, *J* = 4.2 Hz).

MS-ESI (m/z) : [M+H]^+^ calc. for C_18_H_16_FNO 281,1286; found [M+H]^+^ 281,1289

potassium (E)-2-(2-hydroxystyryl)-3,3-dimethyl-3H-indole-5-sulfonate (**1f**): A solution of 2c (0.50 g, 3.61 mmol), salicylaldehyde (4.0 ml, 36.68 mmol), and piperidine (0.07 ml, 0.71 mmol) in absolute ethanol (5 ml) is heated to reflux for 16-24h. After cooling the solution at room temperature, 10 ml of THF are added and the solution is stirred for 5 minutes, then the solution is filtered through a 0.45 microns size syringe filter directly into 300 ml of diethyl ether under vigorous stirring. The resulting yellow solid is filtered on a glass filter por.3, washed with diethyl ether (3 x 25 ml), and dried in a vacuum oven at 75°C to yield 1b as a beige solid (0.49 g, 36%).

^1^H NMR (400 MHz, DMSO-d6) δ 8.01 (d, *J* = 16.3 Hz, 1H), 7.73 (d, *J* = 7.8 Hz, 1H), 7.66 (s, 1H), 7.57 (d, *J* = 8.0 Hz, 1H), 7.42 (d, *J* = 8.0 Hz, 1H), 7.24 – 7.15 (m, 2H), 6.91 (d, *J* = 8.2 Hz, 1H), 6.83 (t, *J* = 7.5 Hz, 1H), 1.38 (s, 6H).

^13^C NMR (101 MHz, DMSO-d6) δ 185.00, 156.83, 154.38, 146.24, 145.82, 133.59, 131.05, 128.01, 125.85, 123.00, 119.76, 119.46, 119.22, 118.99, 116.58, 52.68, 23.51.

MS-ESI (m/z) : [M+H]^+^ calc. for C_18_H_16_KNO_4_S 382,0505; found [M+H]^+^ 382.0510

(E)-2-(2-methoxystyryl)-3,3-dimethyl-3H-indole (**1g**): A solution of 2,3,3-trimethyl indolenine (1 ml, 6.22 mmol), 2-methoxy benzaldehyde (1.04 g, 7.46 mmol), and piperidine (0.12 ml, 1.24 mmol) in absolute ethanol (8 ml) is heated to reflux for 16-24h. The solvent is then removed at reduced pressure, and the crude is purified by flash chromatography (1:1 EtOAc : Hex) over SiO_2_ (rf: 0.72). The resulting orange wax is dissolved in 100 ml of 1M HCl and extracted with 1x 100ml of diethyl ether. Add 100 mf of DCM to the water phase and basify the pH to neutral using NaHCO_3_ , then extract (3x100 ml DCM). Dry the orghanicv phase over Na_2_SO_4_ and remove the volatiles under reduced pressure to yield 1g as thick orange oil (1.27 g, 74%).

^1^H NMR (400 MHz, CDCl_3_) δ 8.02 (d, *J* = 16.7 Hz, 1H), 7.68 – 7.61 (m, 2H), 7.37 – 7.29 (m, 3H), 7.26 – 7.20 (m, 1H), 7.17 (d, *J* = 16.7 Hz, 1H), 7.02 – 6.96 (m, 1H), 6.93 (dd, *J* = 8.3, 1.1 Hz, 1H), 3.92 (s, 3H), 1.49 (s, 6H).

^13^C NMR (101 MHz, CDCl_3_) δ 183.94, 157.84, 153.92, 146.82, 133.34, 130.49, 127.75, 127.54, 125.48, 125.08, 121.03, 120.81, 120.80, 120.57, 111.06, 55.54, 52.64, 24.20.

MS-ESI (m/z) : [M+H]^+^ calc. for C_19_H_19_NO 278,1539; found [M+H]^+^ 278,1537

**2.3. Synthesis of Polymers**

**3-(isobutoxysulfonyl)propyl methacrylate monomer**: 3-(isobutoxysulfonyl)propyl methacrylate has been prepared following a reported literature procedure.^[5]^

^1^H-NMR (400 MHz, CDCl_3_): δ (ppm) = 0.98 (d, 2 CH3), 1.94 (s, CH3), 2.03 (m, CH), 2.25 (m, CH2), 3.21 (t, CH2), 4.00 (d, CH2), 4.28 (t, CH2), 5.60 (s, CH), 6.11 (s, CH).

**PEG methacrylate co sodium 3-(methacryloyloxy)propane-1-sulfonate copolymer (PSPMANa-co-POEGMA)**:

OEGMA (Molecular weight: 500 g/mol) was passed through a basic alumina column to remove inhibitor. AIBN was recrystallized before the polymerization.

PBSPMA-co-POEGMA was synthesized by RAFT polymerization. 1.425g OEGMA (2.85 mmol, 57 equiv.), 42.4 mg BSPMA (42.4 mg, 3.2 equiv.) and 20.2 mg 4-Cyano-4-[[(dodecylthio)carbonothioyl]thio]pentanoic Acid (CDPA) (0.05 mmol, 1 equiv.)were added in a round bottom flask which equipped with a stirring egg. Recrystallized AIBN was dissolved in DMF to prepare a stock solution (4mg ml-1). Then, 0.205 ml AIBN (0.82 mg, 5*10-3 mmol, 0.1 equiv.) and 2 ml DMF were added in the flask. The solution was stirred until uniform. Then the flask was put into 70 ℃ oil bath after the yellowish solution was sparged with N_2_ for 15 minutes. The reaction was stirred and heated overnight. After 20 h, the polymerization was stopped by putting the flask into ice bath. Then, the solution was precipitated into 6:1 n-Hexane/Ethanol for three times. Finally, the product was redissolved with little amount acetone, transferred to a glass vials, and dried with a vacuum oven at 70 ℃ overnight to get the PBSPMA-co-POEGMA. Yield: 1.1176g, 76.18 %.

Figure S1: ^1^H-NMR spectra of PBSPMA-co-POEGMA in CDCl_3_

The PBSPMA-co-POEGMA was deprotected following a reported literature peocedure.^1^ 1 g PBSPMA-co-POEGMA was dissolved in 10 ml DMSO. According to the molar of BSPMA unit, 3 equivalents of NaI (92.5 mg, 0.37 mmol) was added to the solution. Then, the solution was heated to 70 ℃ overnight. After 24h, the solution was precipitated into 2:1 n-Hexane/Ethanol and then was washed with 4:1 n-Hexane/Ethanol for three times. Finally, the product was transferred to another glass vials and dried with a vacuum oven at 70 ℃ overnight to get PSPMANa-co-POEGMA. Yield: 873.6 mg. 1H-NMR: deprotection ≈ 100 %.

Figure S2: ^1^H-NMR spectra of PSPMANa-co-POEGMA in D_2_O


**PSPMAH-co-POEGMA (poly-4a**): 43.3 mg of PEG methacrylate co sodium 3-(methacryloyloxy)propane-1-sulfonate are dissolved in 10 ml of methanol and treated with DOWEX proton exchange resin under vigorous stirring for one minute. The obtained solution is filtered through a 0.2 µm syringe filter and used immediately.

Figure S3: ^1^H-NMR spectra of PSPMAH-co-POEGMA (poly-4a) in CD_3_OD

**3. NMR spectra of styryl cyanines**


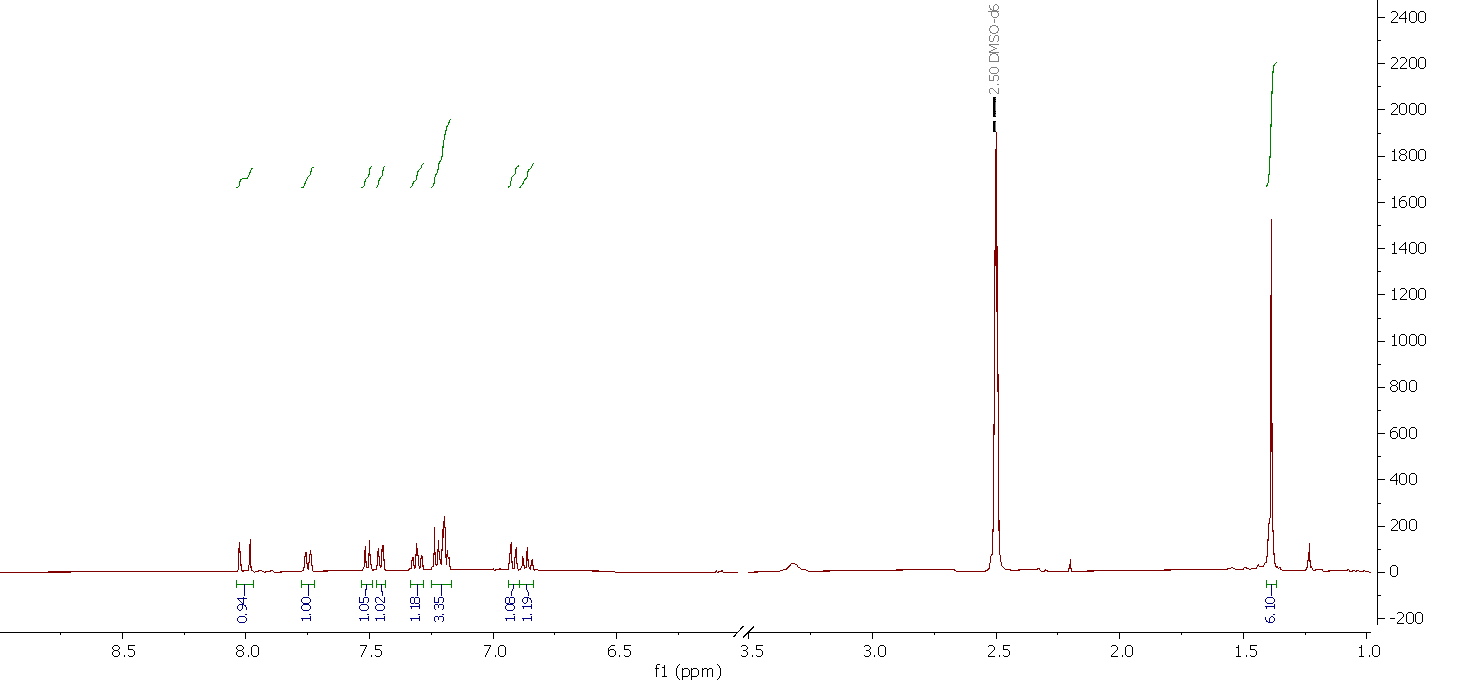

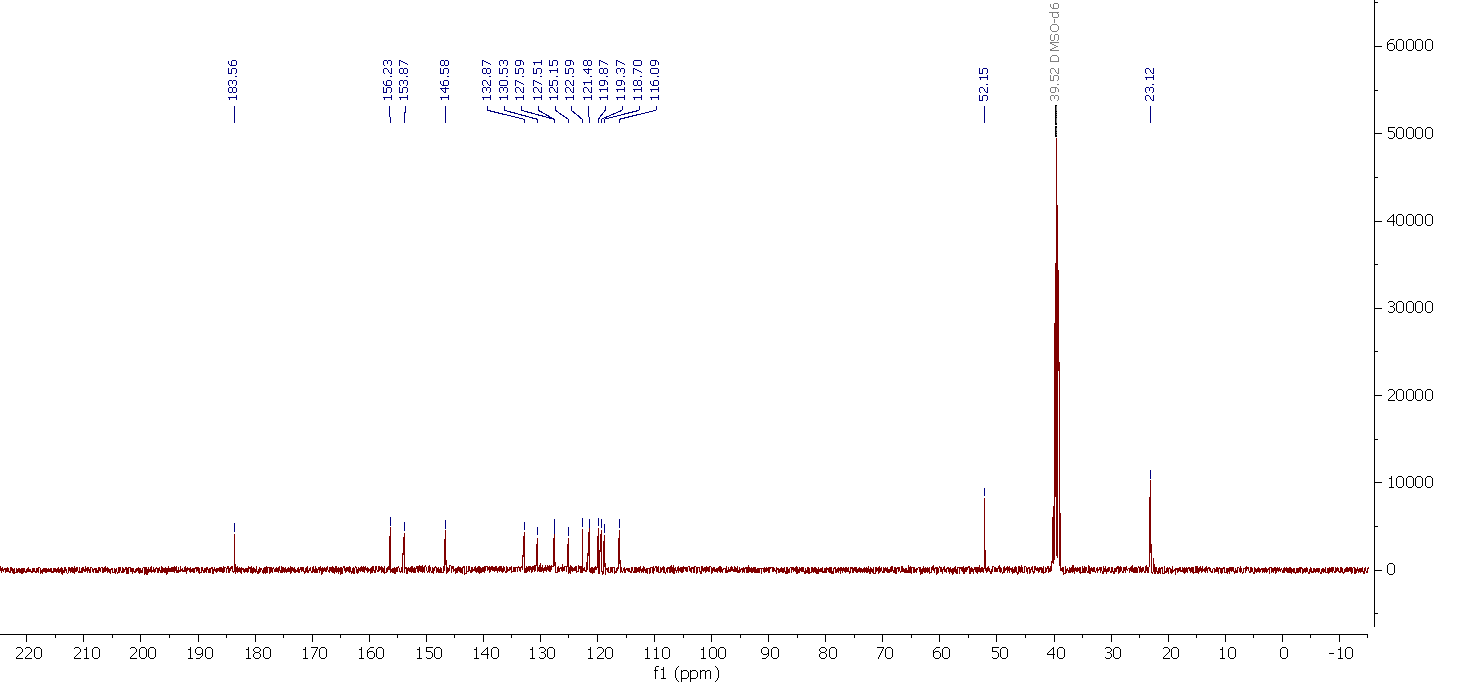


(A)

(B)

Figure S4: (A) ^1^H-NMR spectra of 1a in DMSO-d6 and (B) ^13^C-NMR spectra of 1a in DMSO-d6


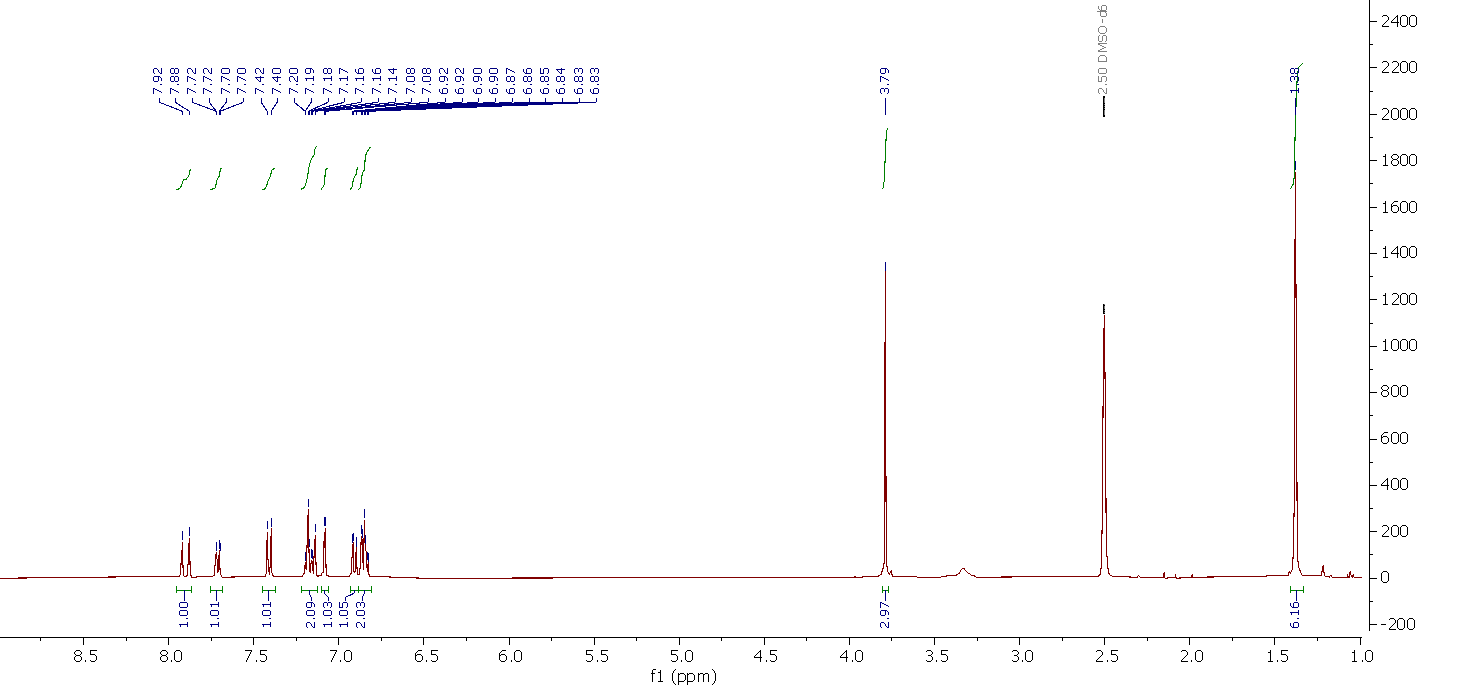

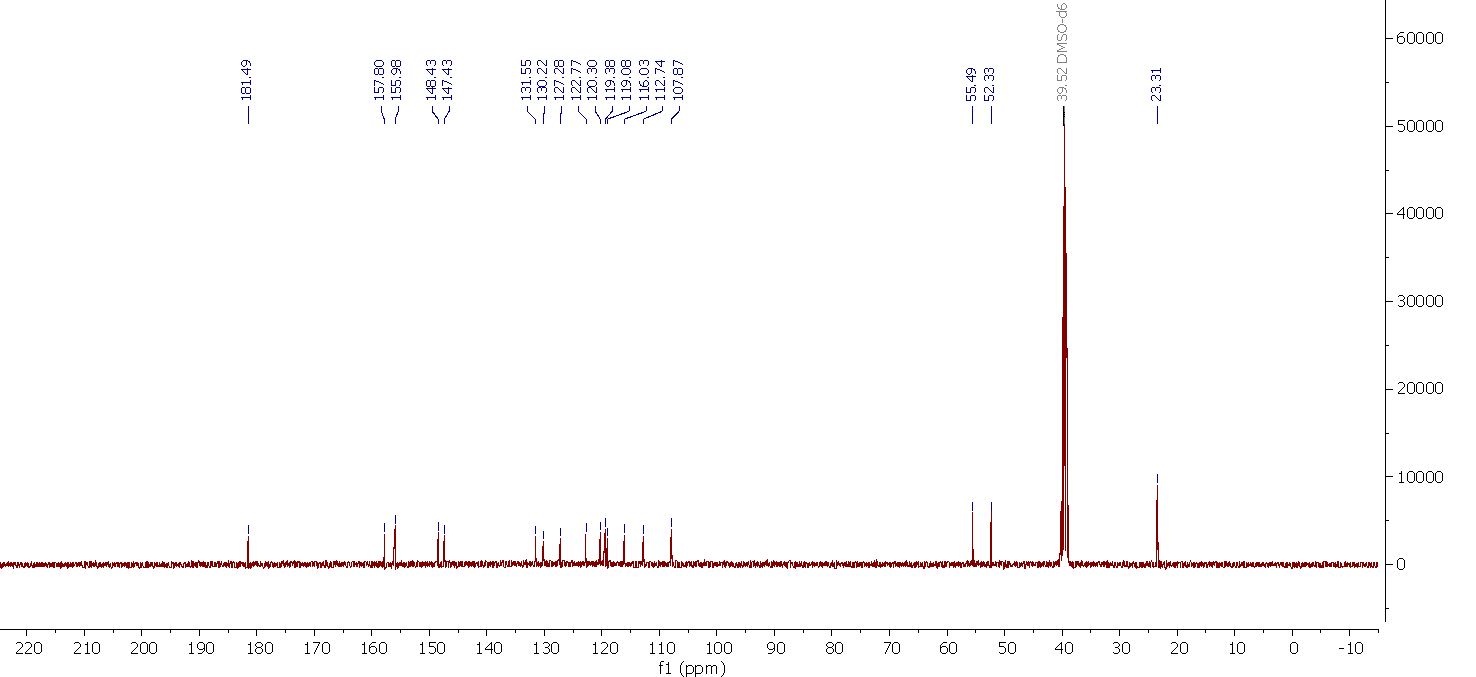


(A)

(B)

Figure S5: (A) ^1^H-NMR spectra of 1b in DMSO-d6 and (B) ^13^C-NMR spectra of 1b in DMSO-d6


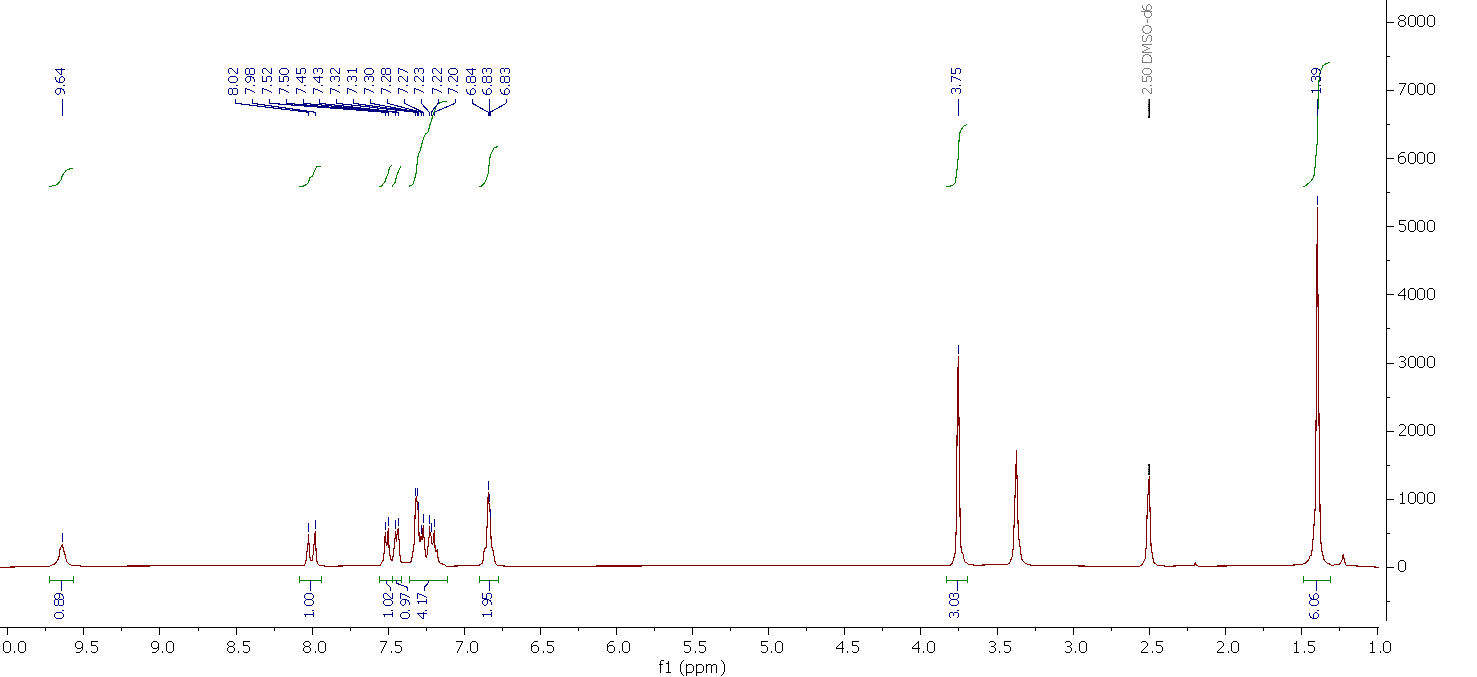

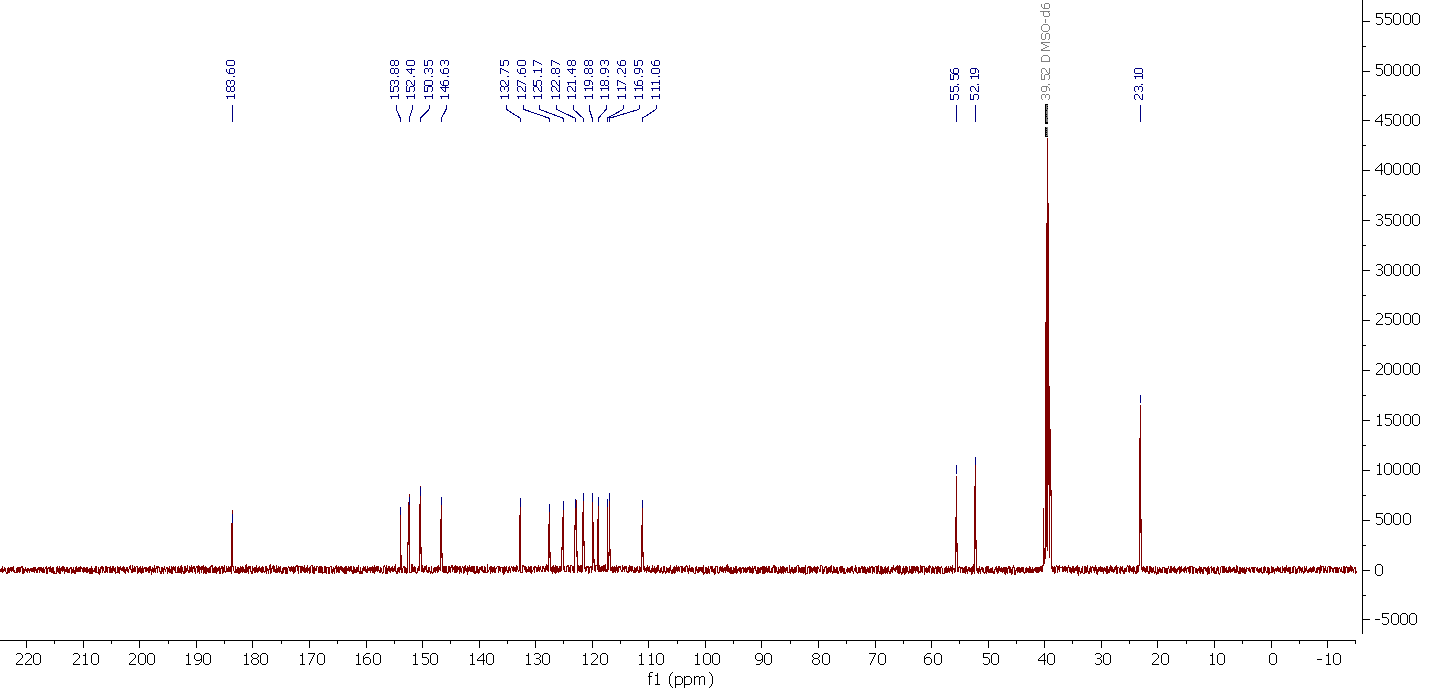


(A)

(B)

Figure S6: (A) ^1^H-NMR spectra of 1c in DMSO-d6 and (B) ^13^C-NMR spectra of 1c in DMSO-d6


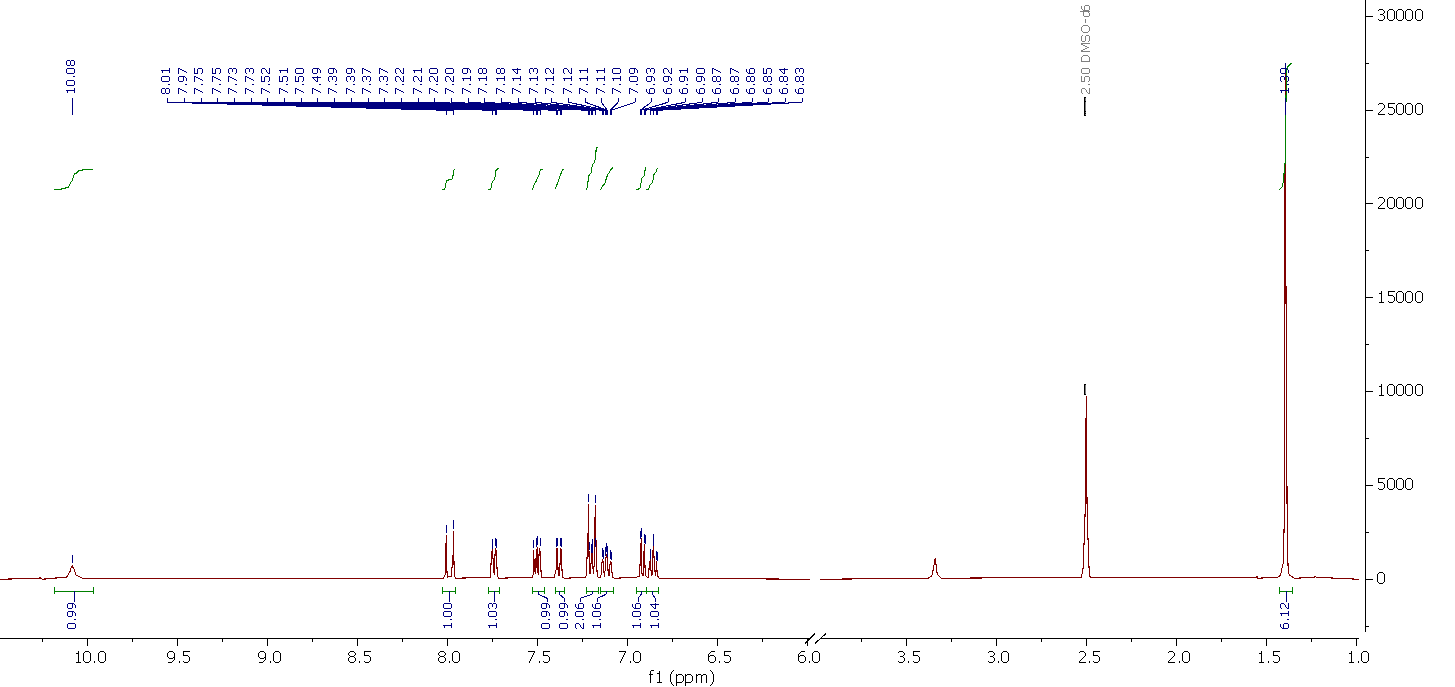

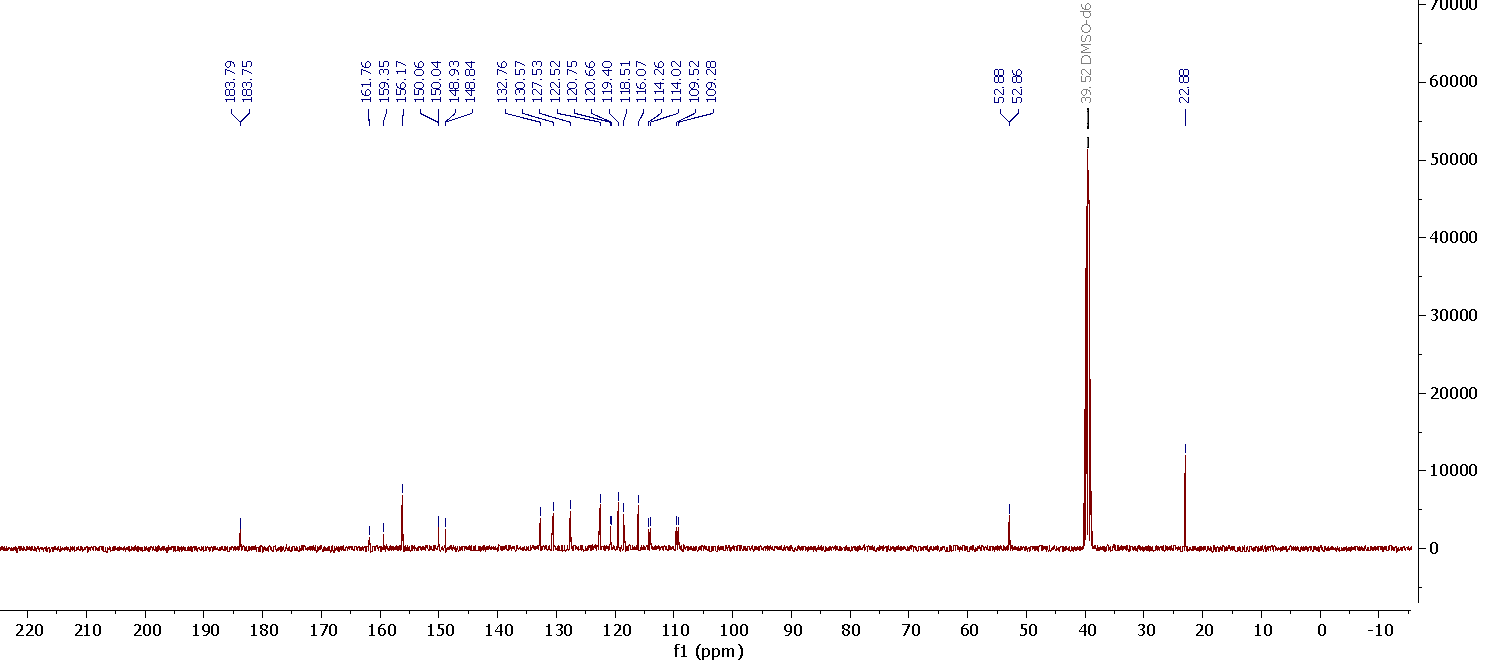


(A)

(B)


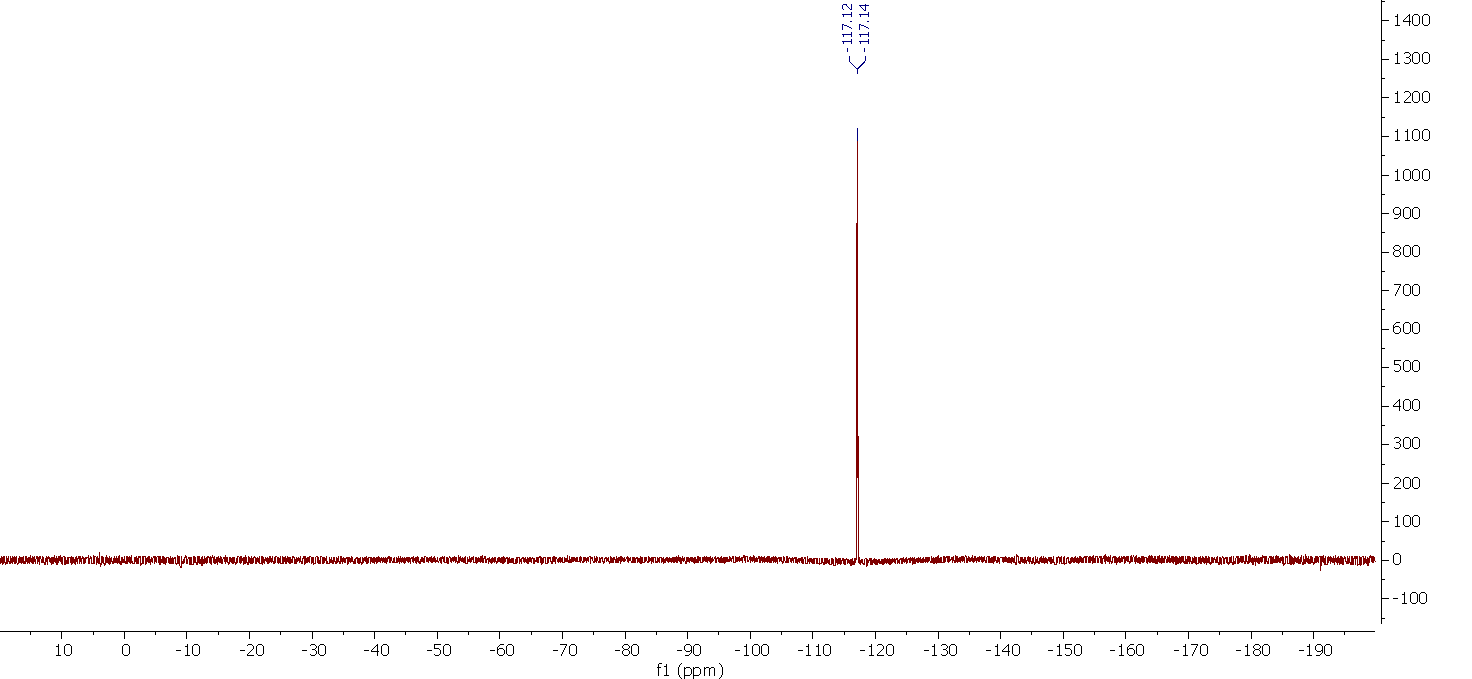


(C)

Figure S7: (A) ^1^H-NMR spectra of 1d in DMSO-d6 (B) ^13^C-NMR spectra of 1d in DMSO-d6 (C) ^19^F-NMR spectra of 1d in DMSO-d6


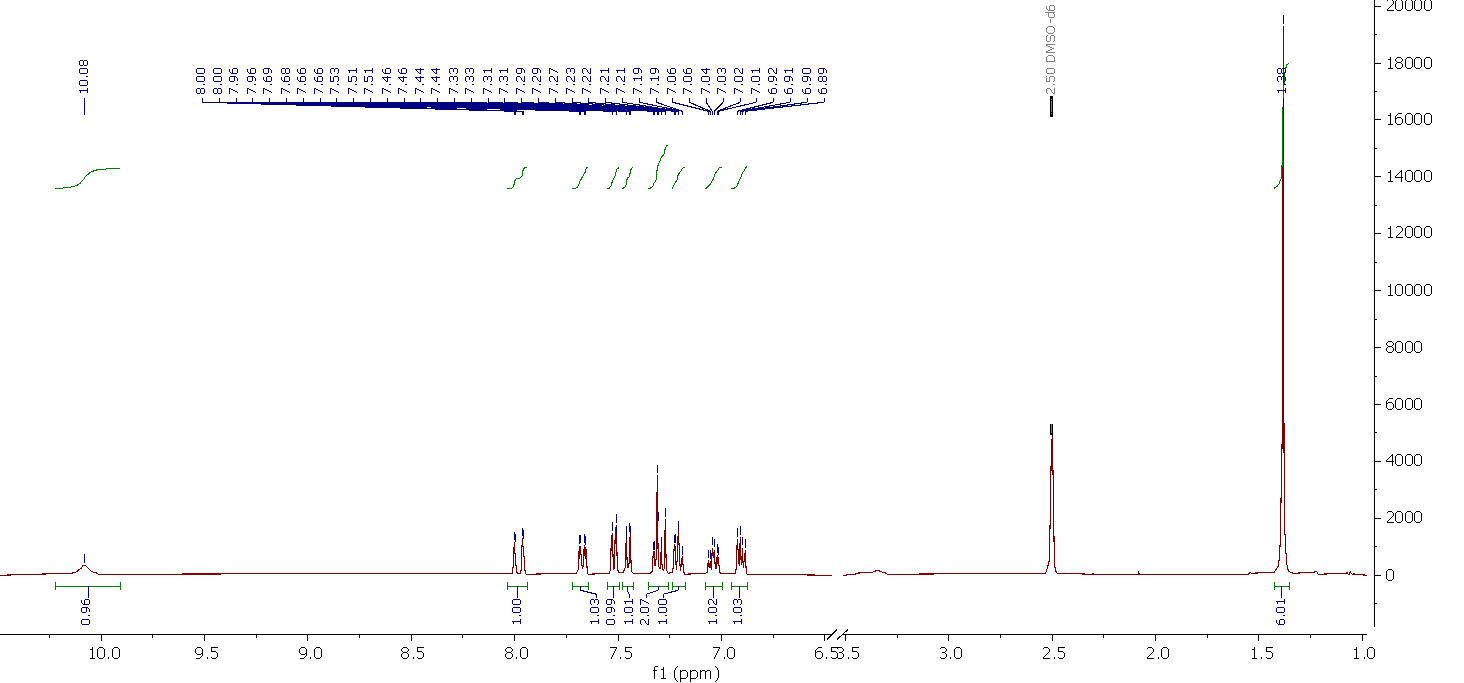

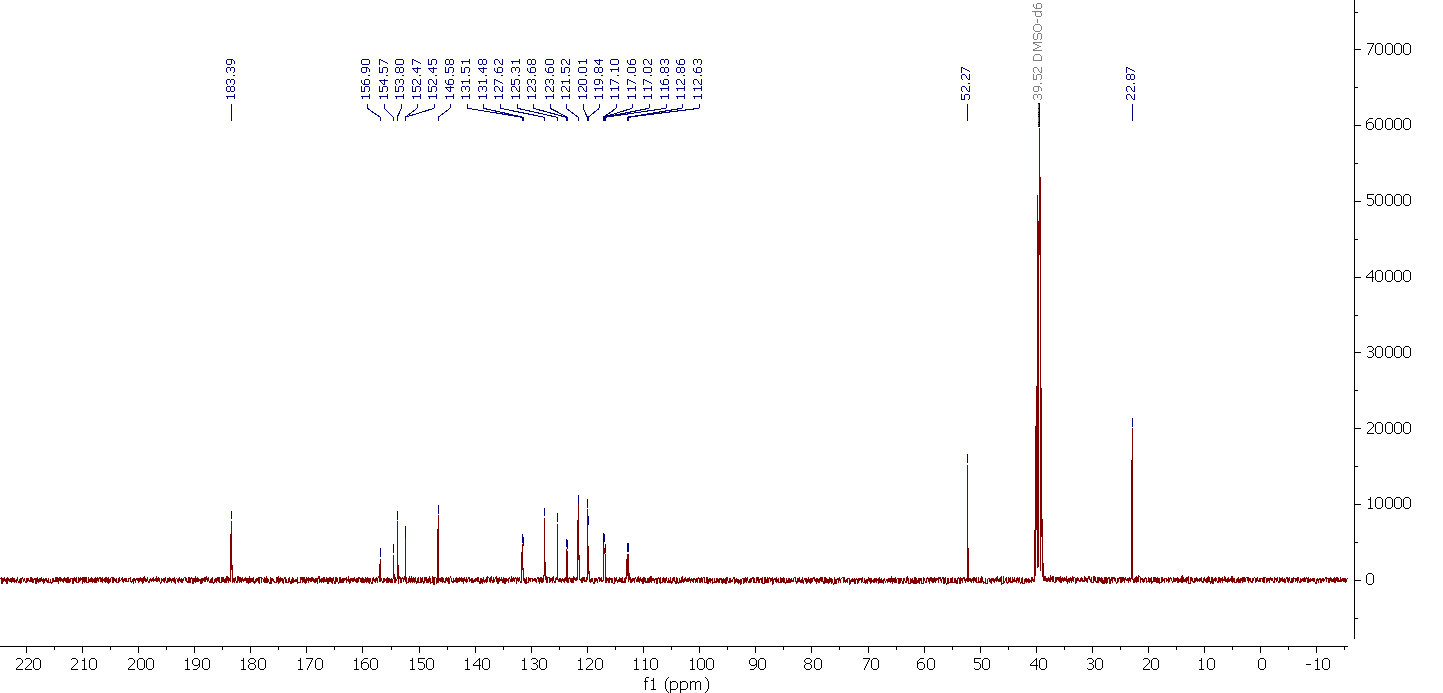

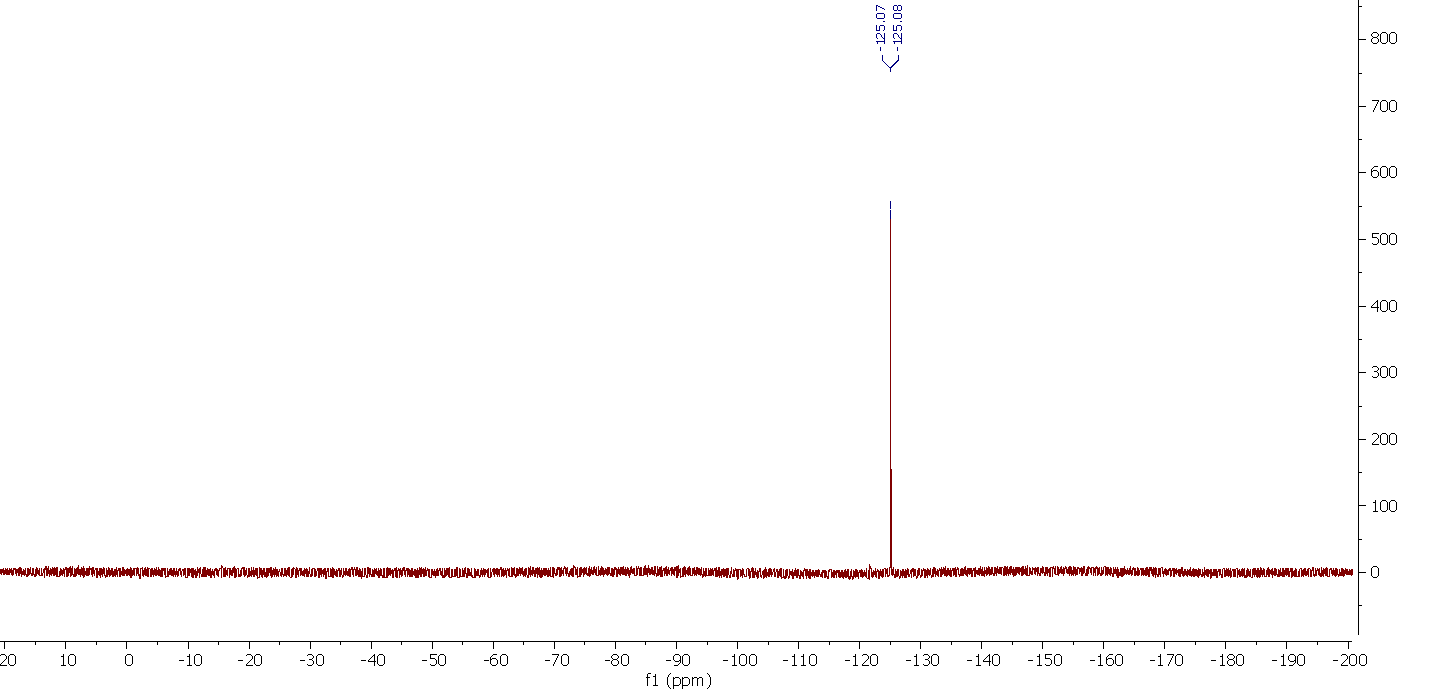


(A)

(B)

(C)

Figure S8: (A) ^1^H-NMR spectra of 1e in DMSO-d6 (B) ^13^C-NMR spectra of 1e in DMSO-d6 (C) ^19^F-NMR spectra of 1e in DMSO-d6


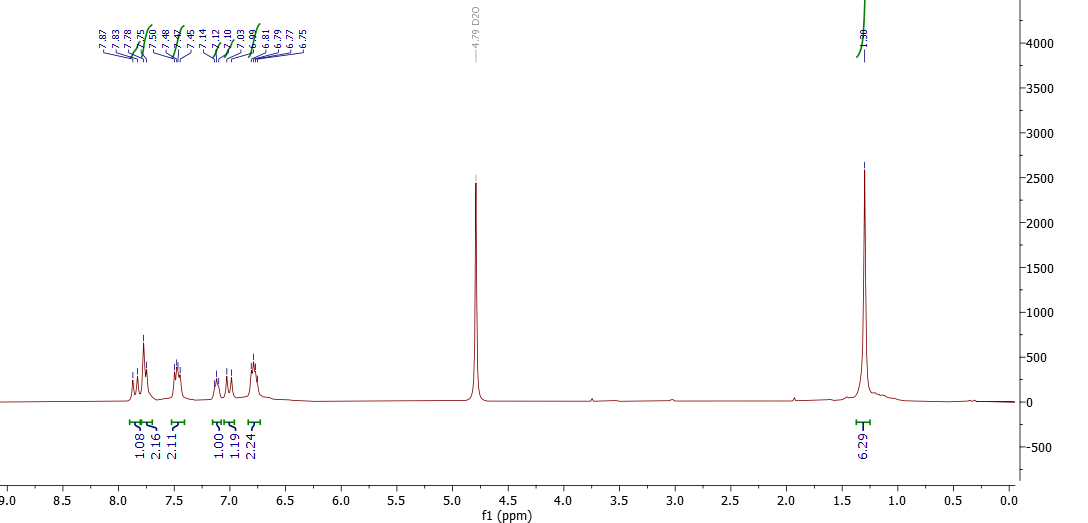


(A)

(B)

(C)

Figure S9: (A) ^1^H-NMR spectra of 1f in DMSO-d6 (B) ^13^C-NMR spectra of 1f in DMSO-d6 (C) ^1^H-NMR spectra of 1f in D_2_O


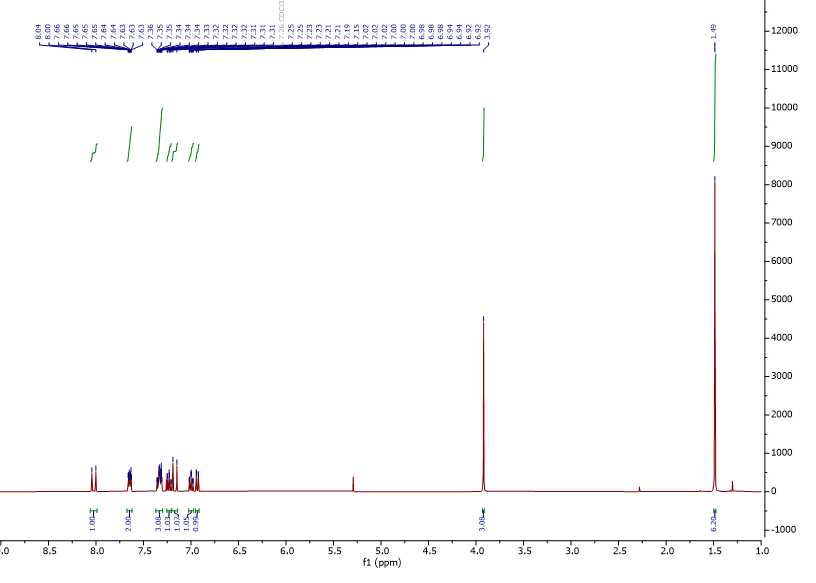

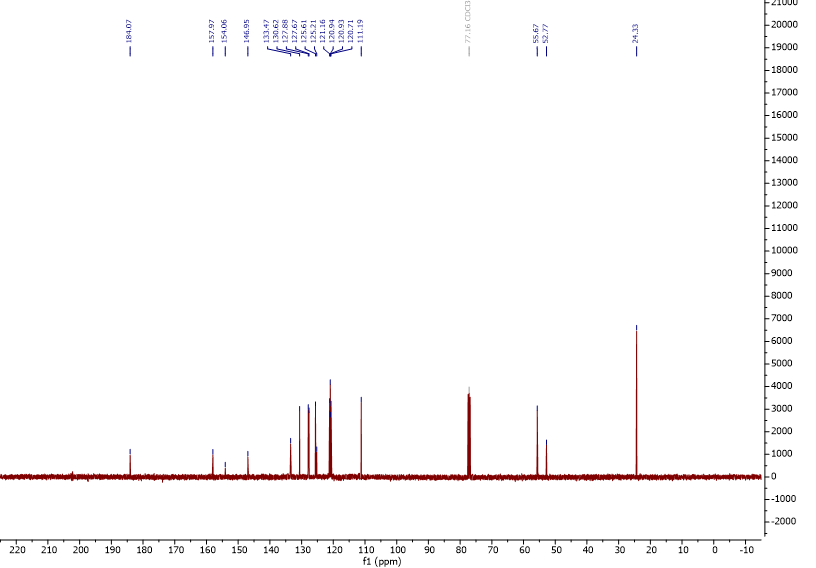


(A)

(B)

Figure S10: (A) ^1^H-NMR spectra of 1g in CDCl_3_ and (B) ^13^C-NMR spectra of 1g in CDCl_3_

**4. In-situ NMR under light irradiation**

**4.1. General procedure**

Samples were prepared by dissolving photoswitches 1a-g in the corresponding deuterated solvent at a concentration of 25 mM. The in situ irradiation at 365 nm (M365FP1, Thorlabs, 1A) has been performed as follow: the fiber optic equipped NMR tube has been placed inside the NMR machine and a spectra is acquired in the dark, then, the LED is turned on and a series of 17 spectra are acquired within a time of 1.5 hours. For each spectra 64 acquisitions have been performed, and two minutes of waiting time have been set between spectra. For each sample the PSS has been reached within the first five acquired spectra and did not change during the remaining time. The ratio between the integrals of the spectra acquired in the dark and the one at PSS has been used to determine the PSS ratio between open and closed isomer of the photoswitches

**4.2. ^1^H-NMR with in situ irradiation**


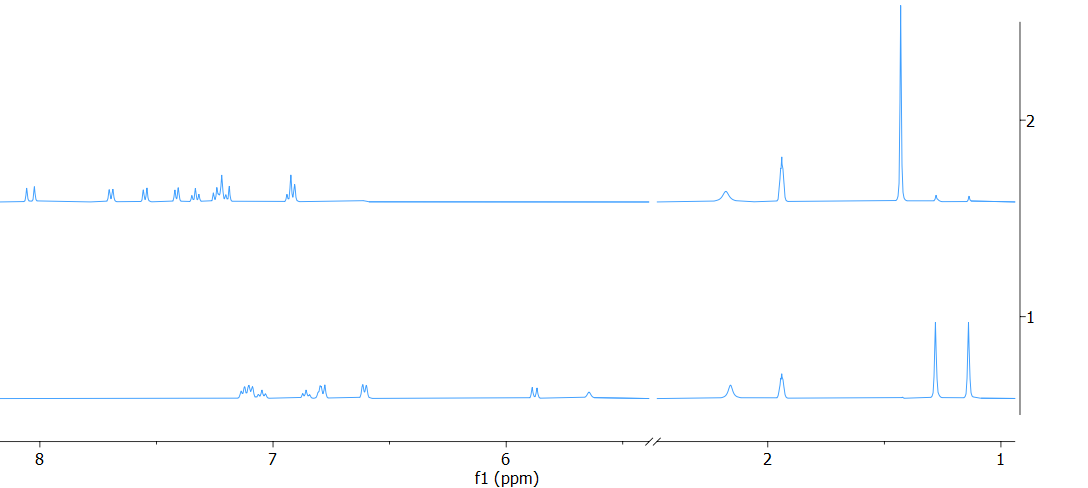

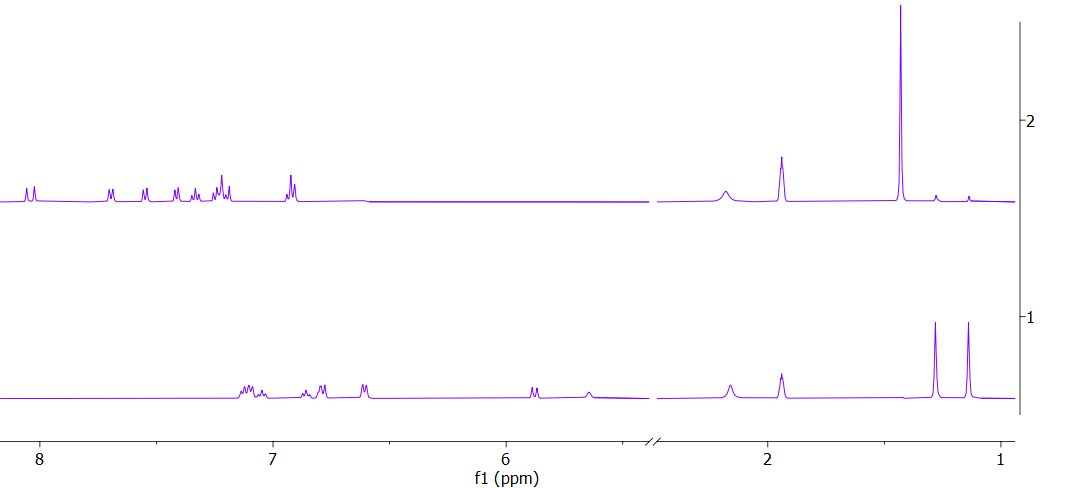


Figure S11: ^1^H-NMR spectra of 1a in CD_3_CN in the dark (purple) and at the PSS (cyan)


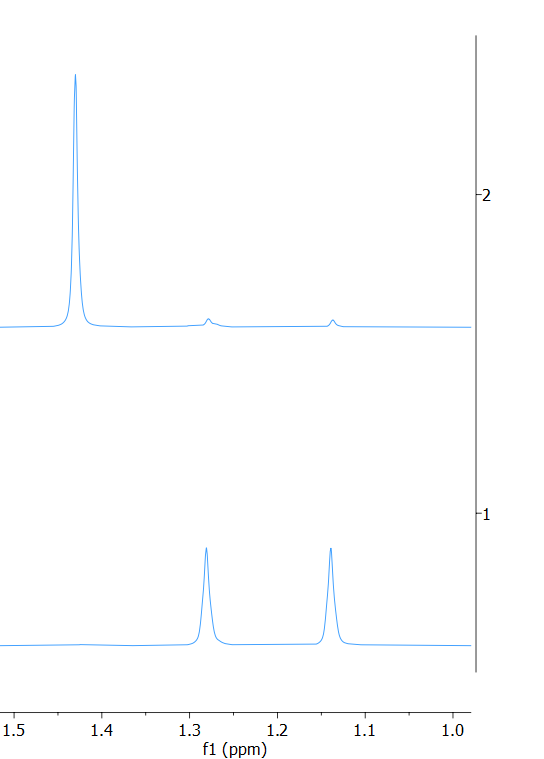

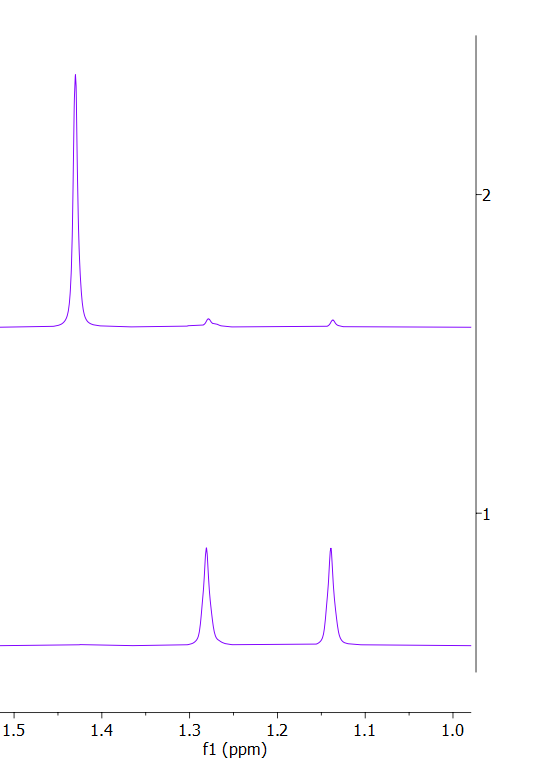


Figure S12: Aliphatic part of the ^1^H-NMR spectra of 1a in CD_3_CN in the dark (purple) and at the PSS (cyan)

Figure S13: ^1^H-NMR spectra of 1b in CD_3_CN in the dark (purple) and at the PSS (cyan)


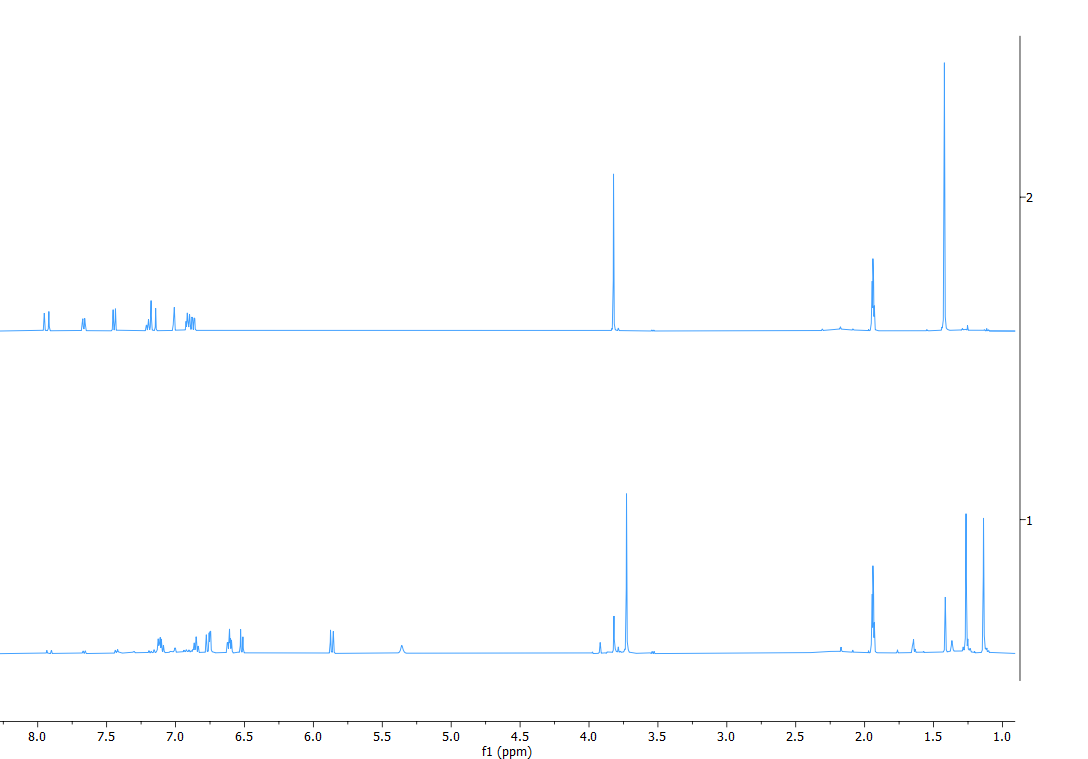

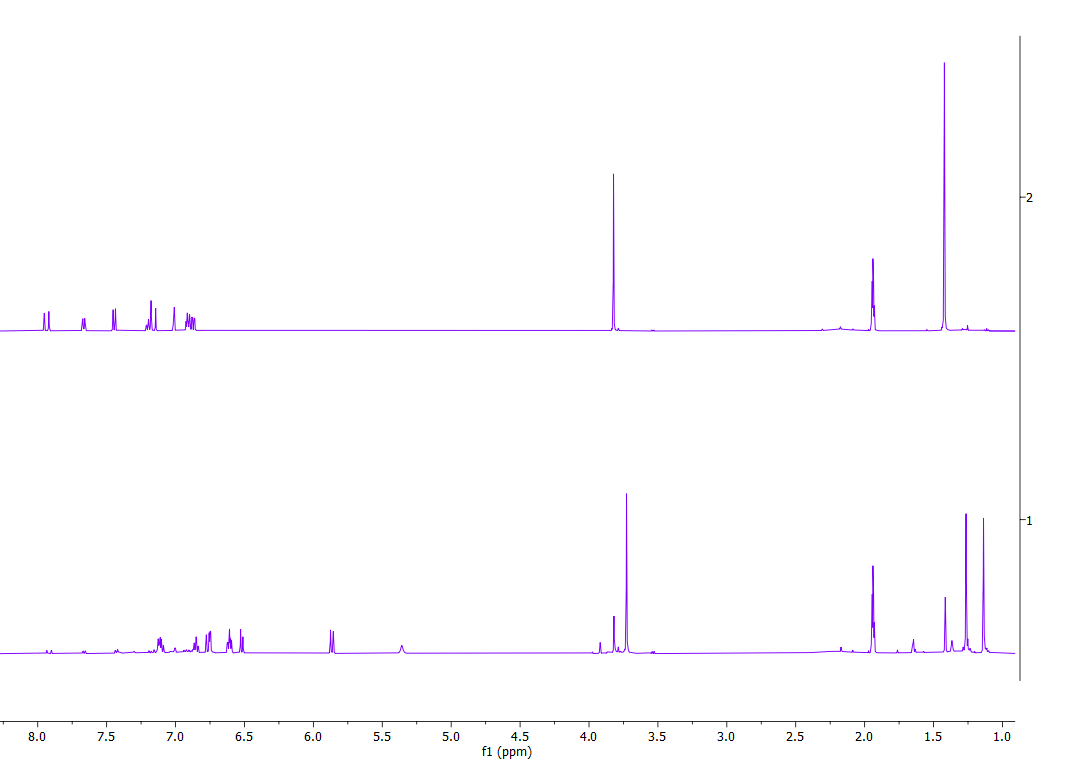

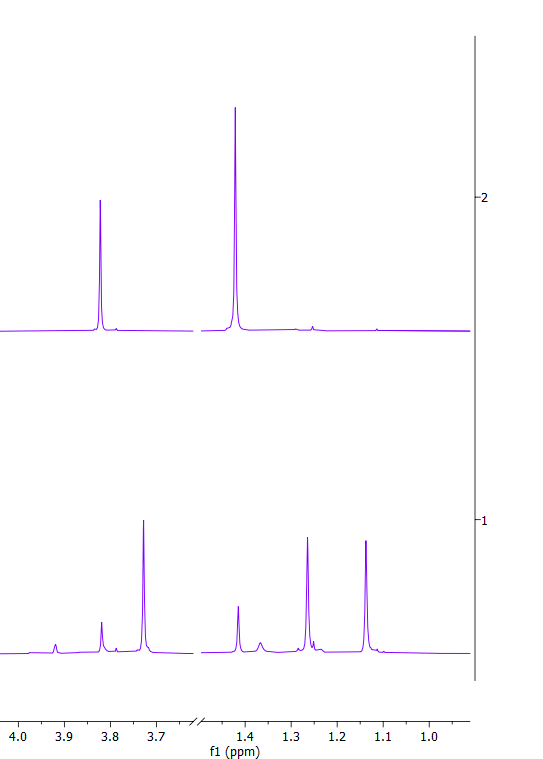

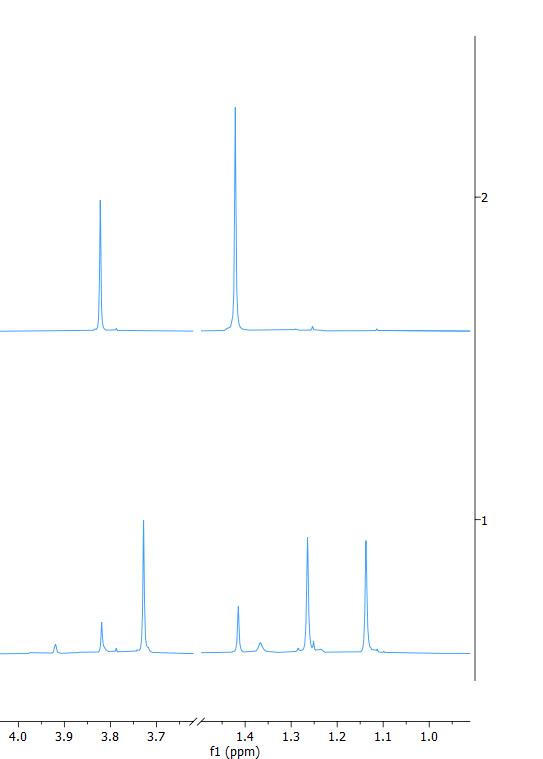


Figure S14: Aliphatic part of the ^1^H-NMR spectra of 1b in CD_3_CN in the dark (purple) and at the PSS (cyan)

Figure S15: ^1^H-NMR spectra of 1c in CD_3_CN in the dark (purple) and at the PSS (cyan)


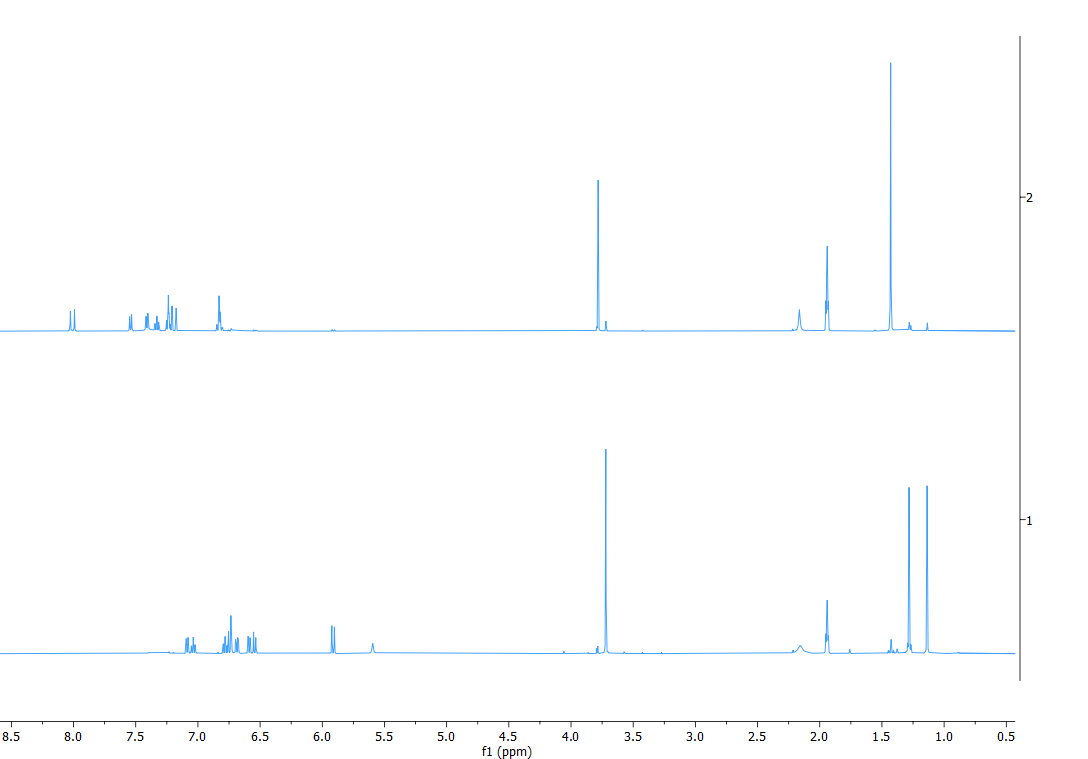

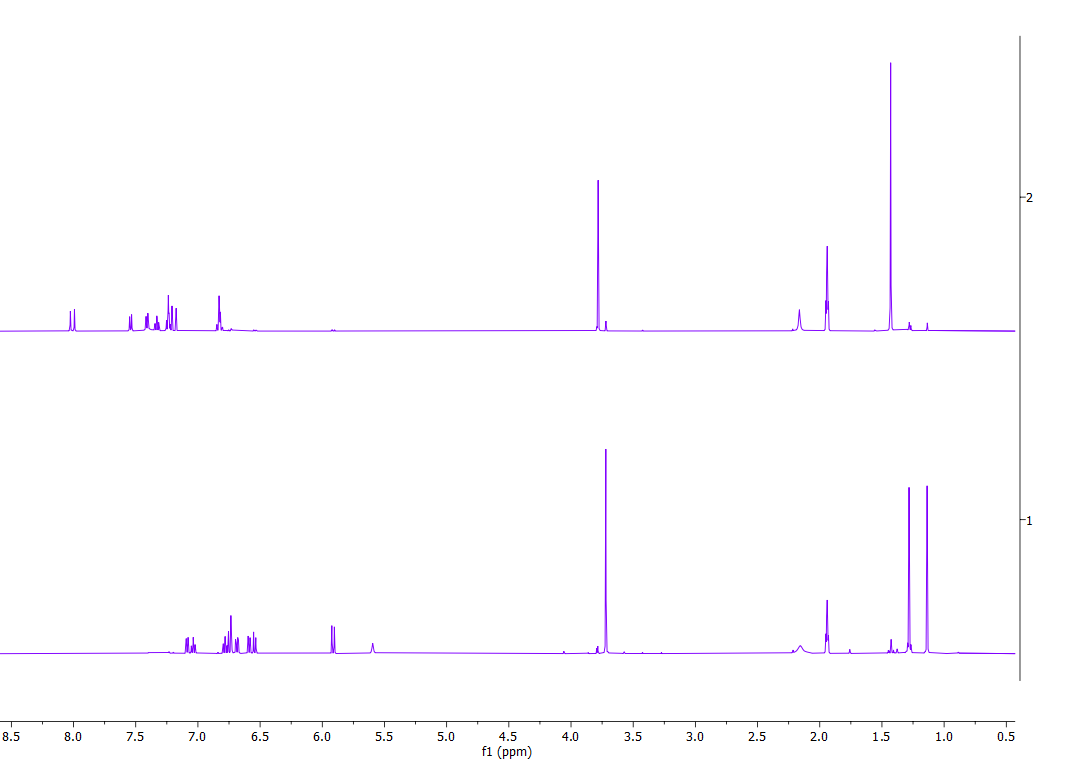

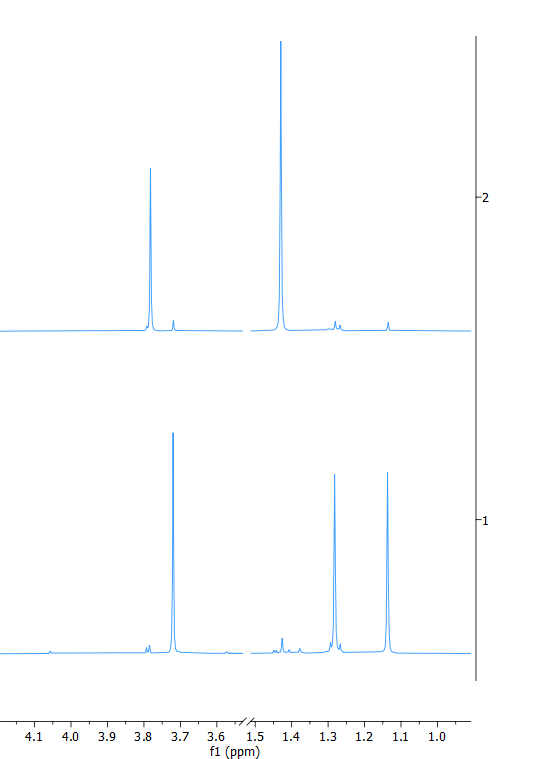

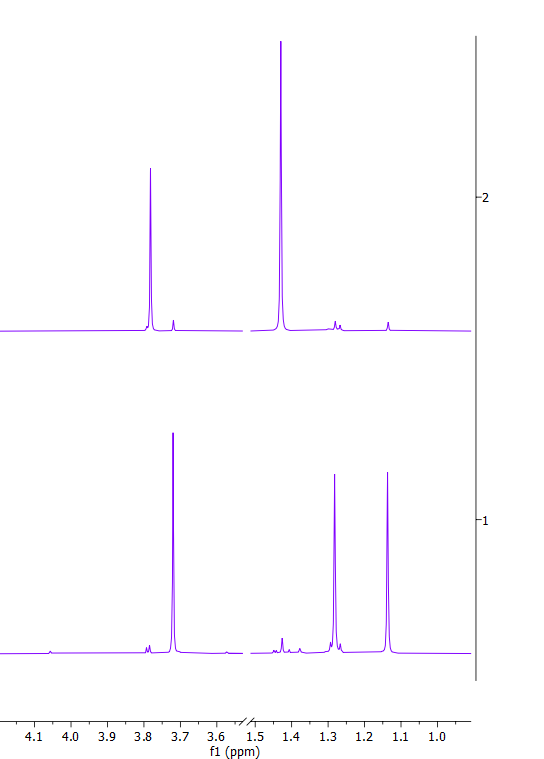


Figure S16: Aliphatic part of the ^1^H-NMR spectra of 1c in CD_3_CN in the dark (purple) and at the PSS (cyan)

Figure S17: ^1^H-NMR spectra of 1d in CD_3_CN in the dark (purple) and at the PSS (cyan)


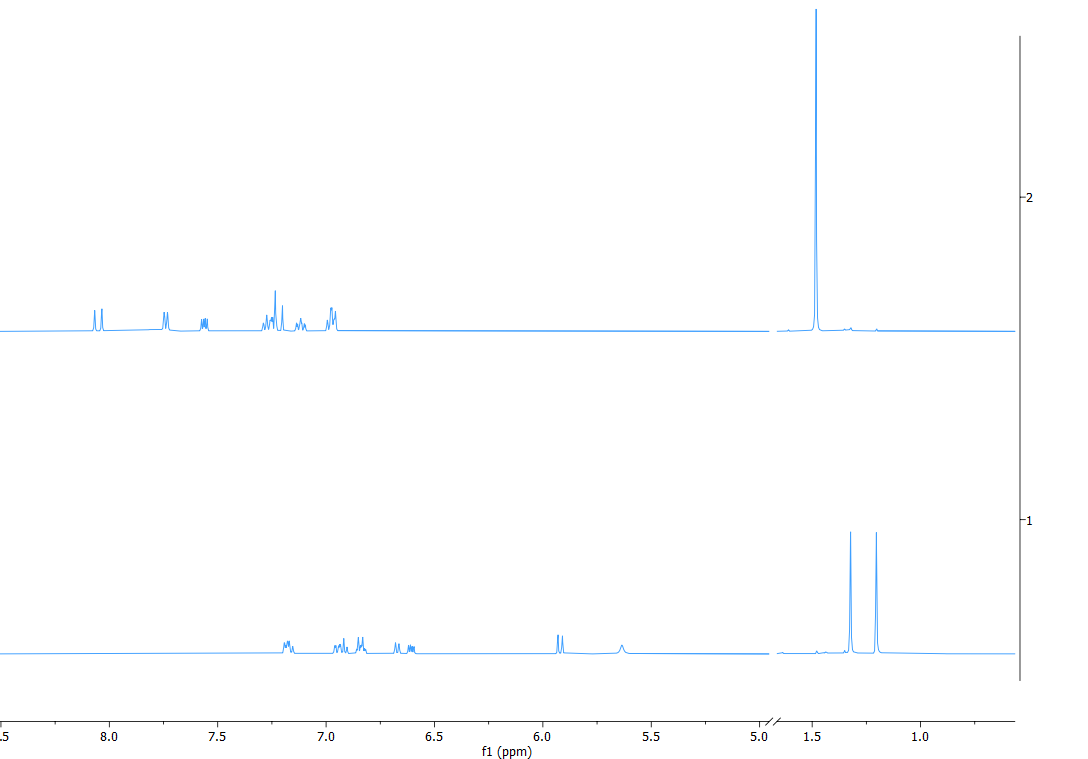

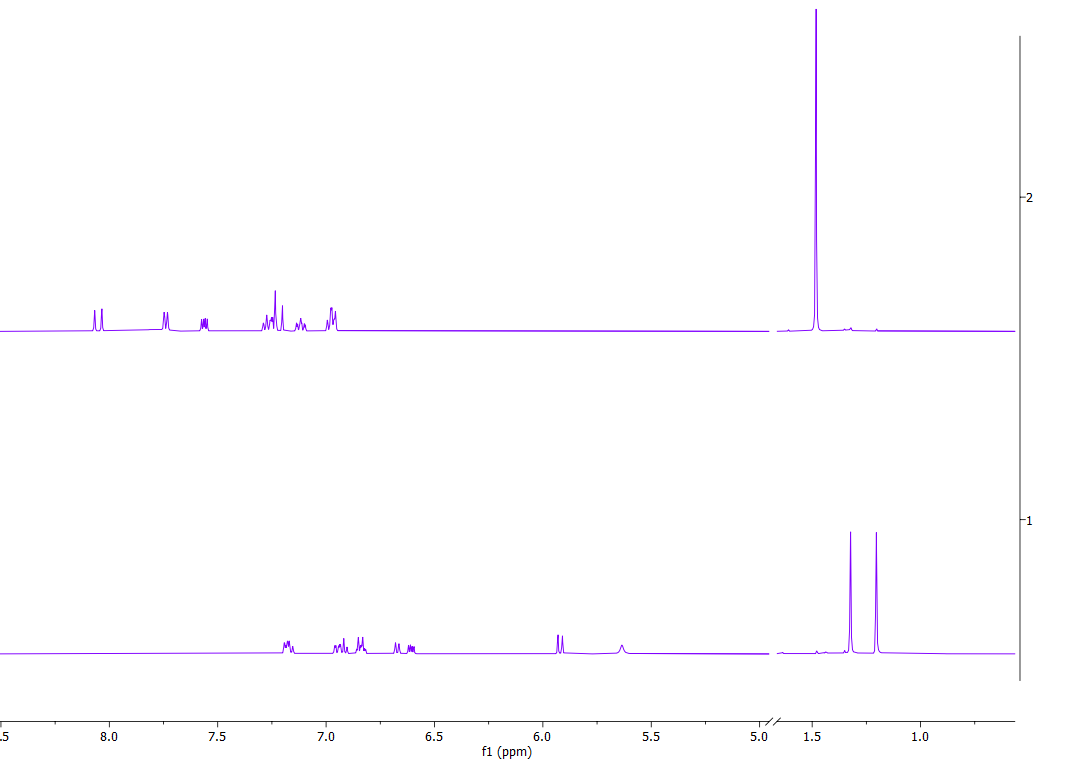

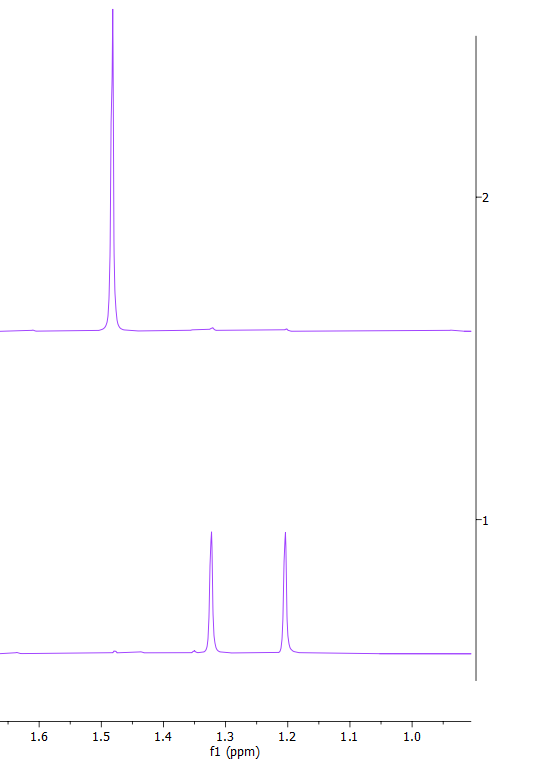

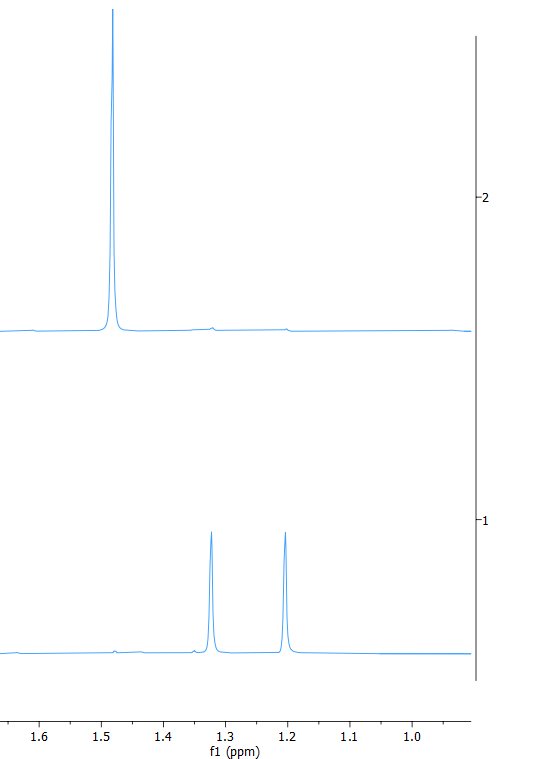


Figure S18: Aliphatic part of the ^1^H-NMR spectra of 1d in CD_3_CN in the dark (purple) and at the PSS (cyan)


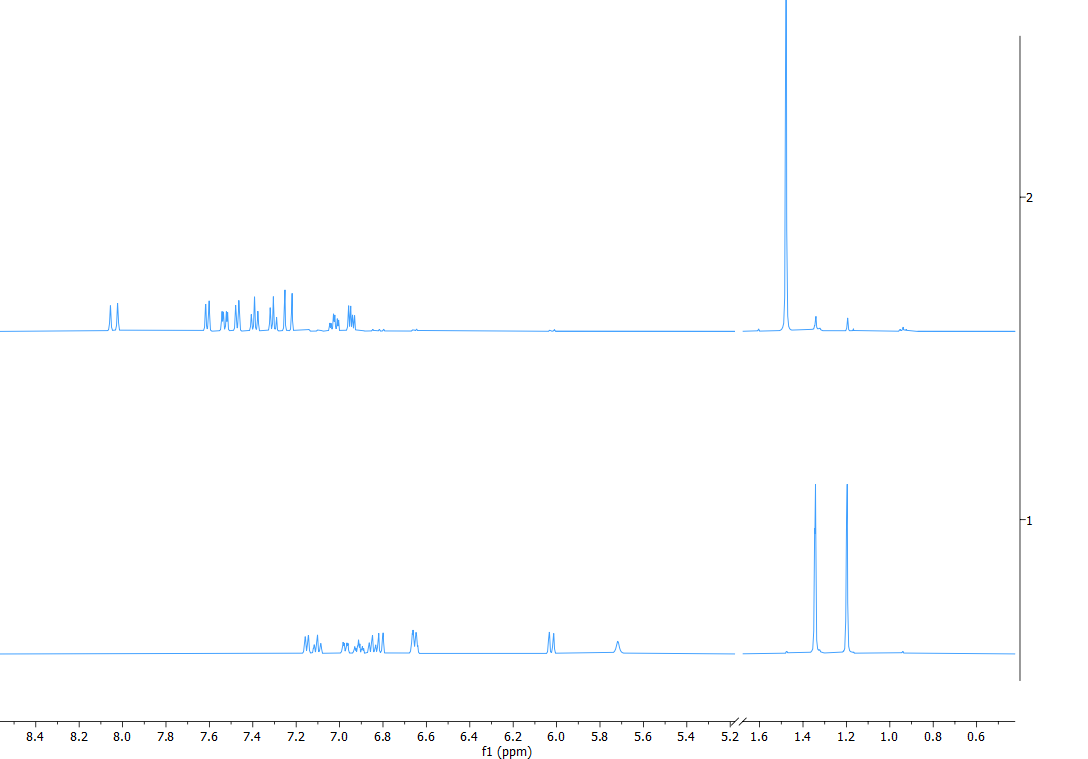

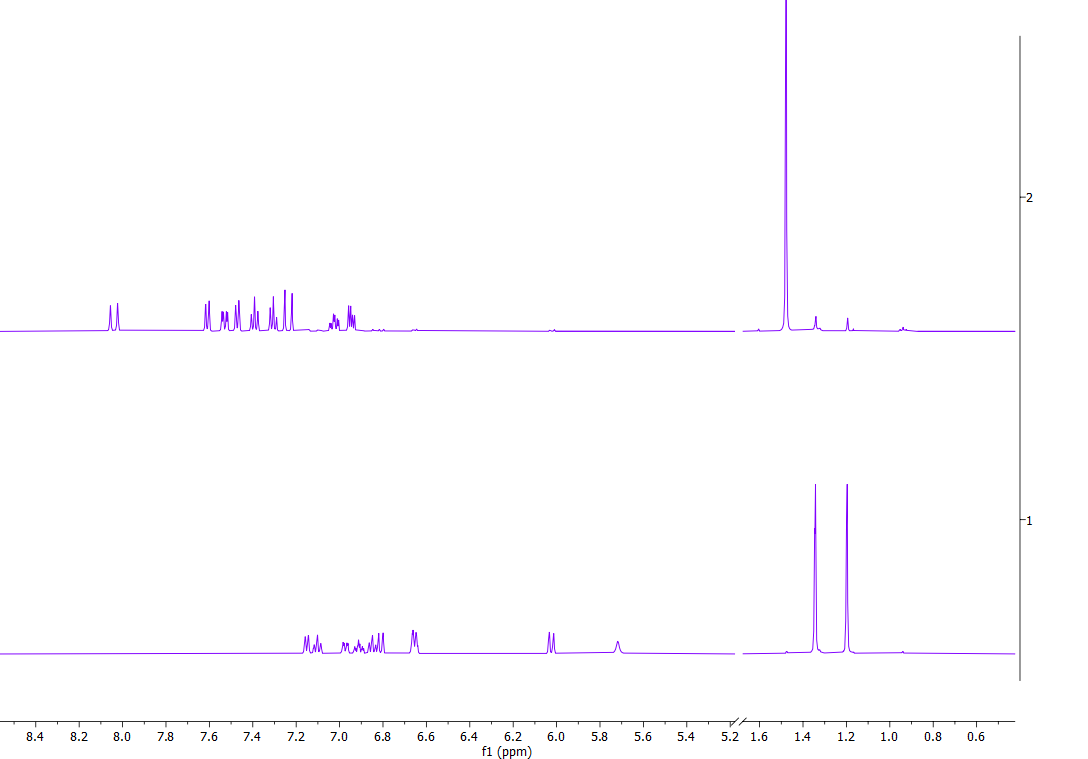


Figure S19: ^1^H-NMR spectra of 1e in CD_3_CN in the dark (purple) and at the PSS (cyan)


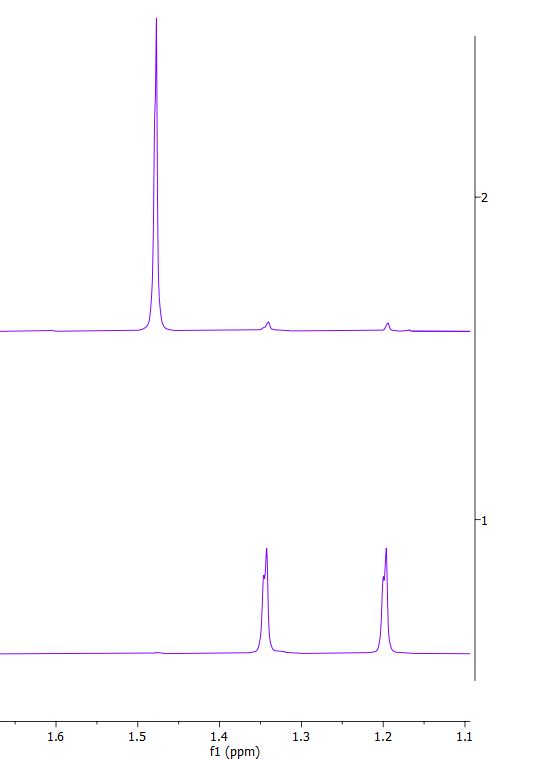

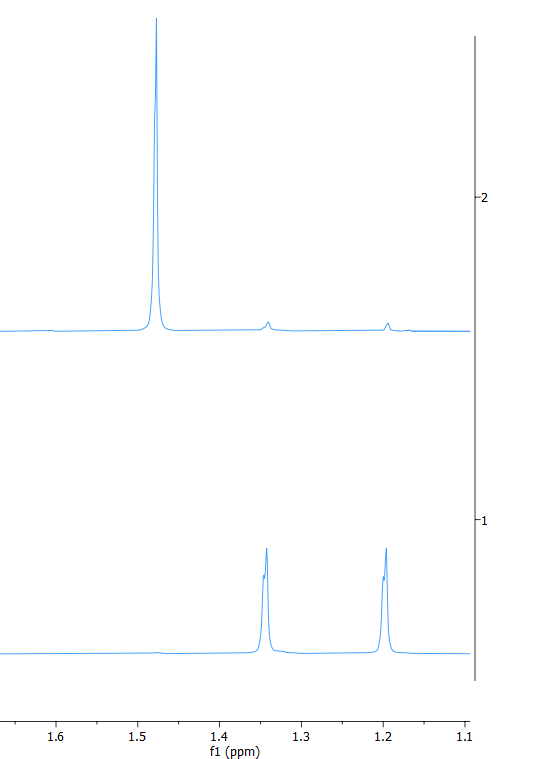


Figure S20: Aliphatic part of the ^1^H-NMR spectra of 1e in CD_3_CN in the dark (purple) and at the PSS (cyan)


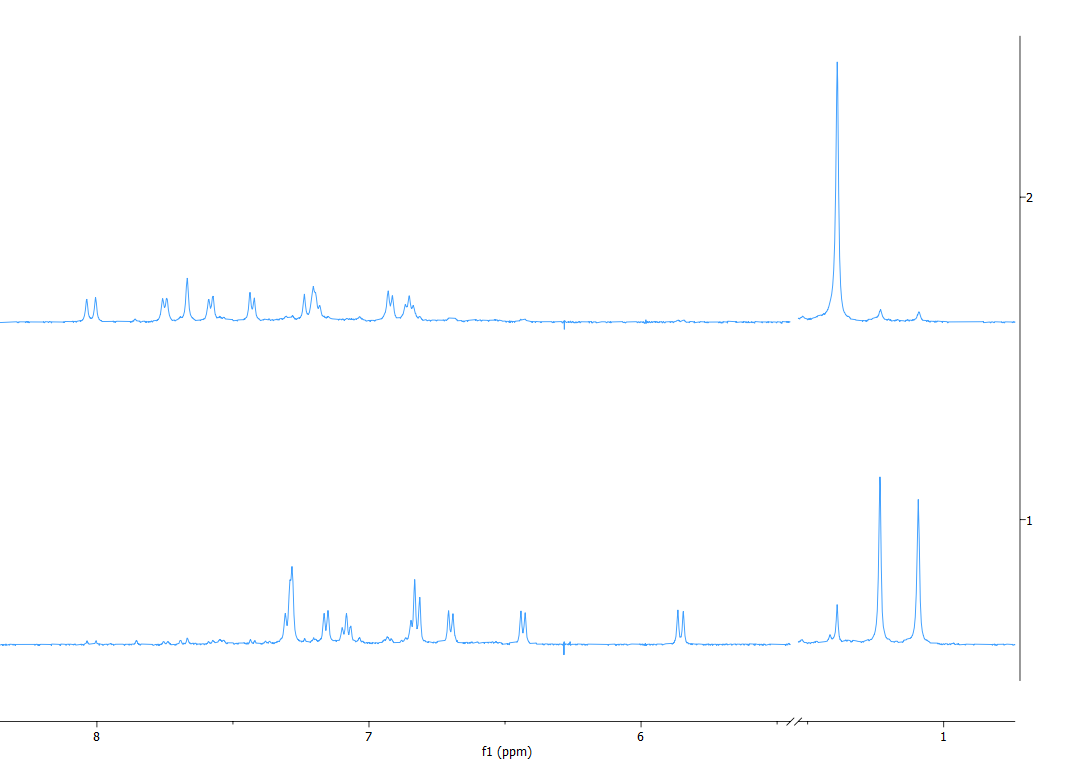

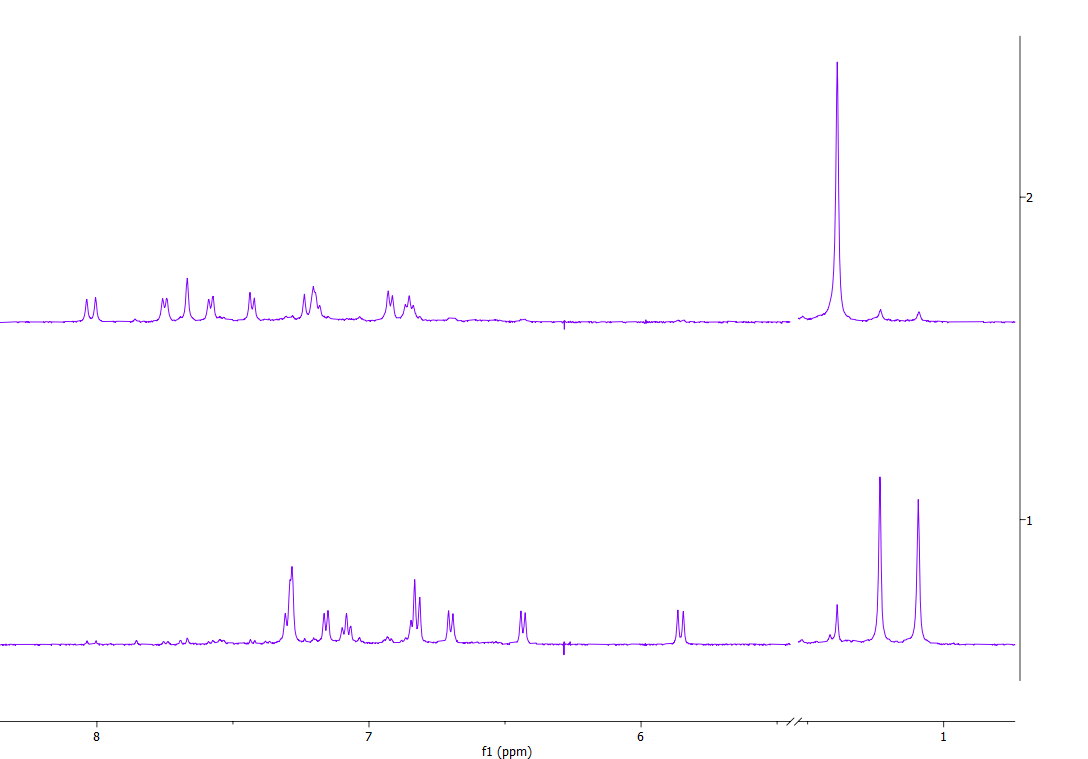


Figure S21: ^1^H-NMR spectra of 1f in DMSOd6 in the dark (purple) and at the PSS (cyan)


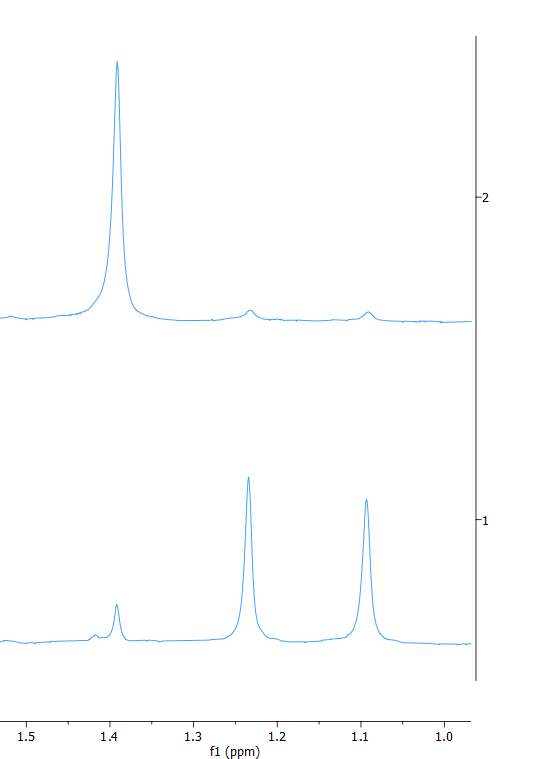

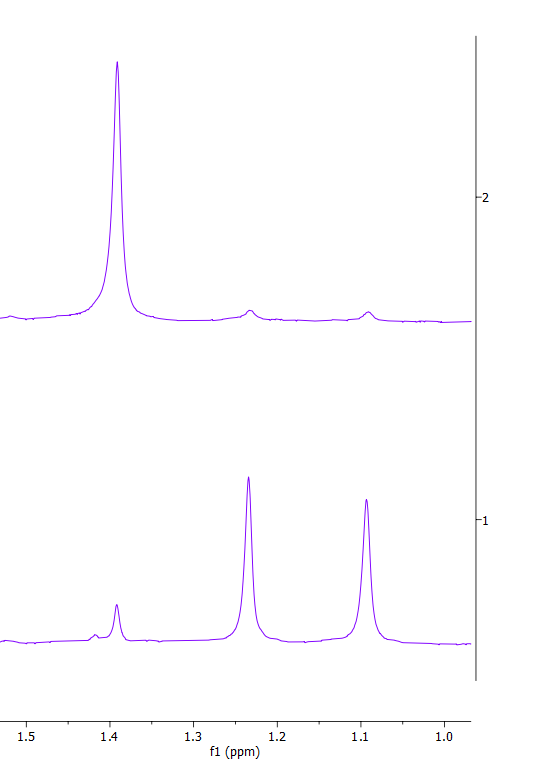


Figure S22: Aliphatic part of the ^1^H-NMR spectra of 1e in DMSOd6 in the dark (purple) and at the PSS (cyan)


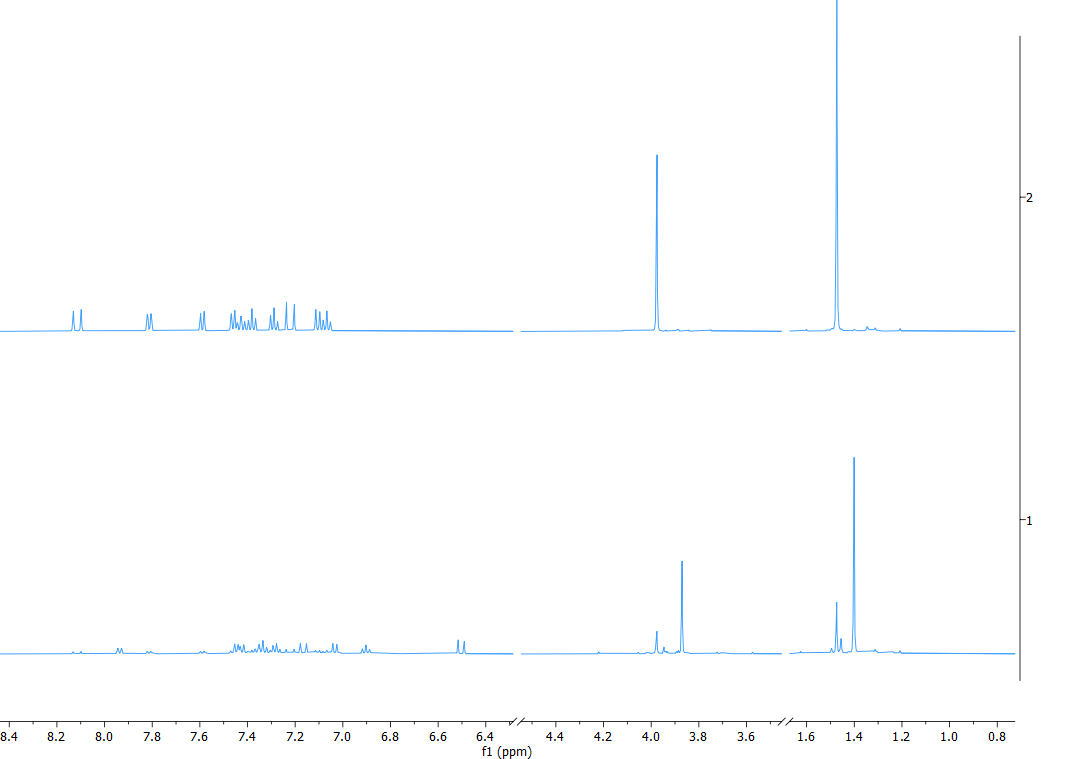

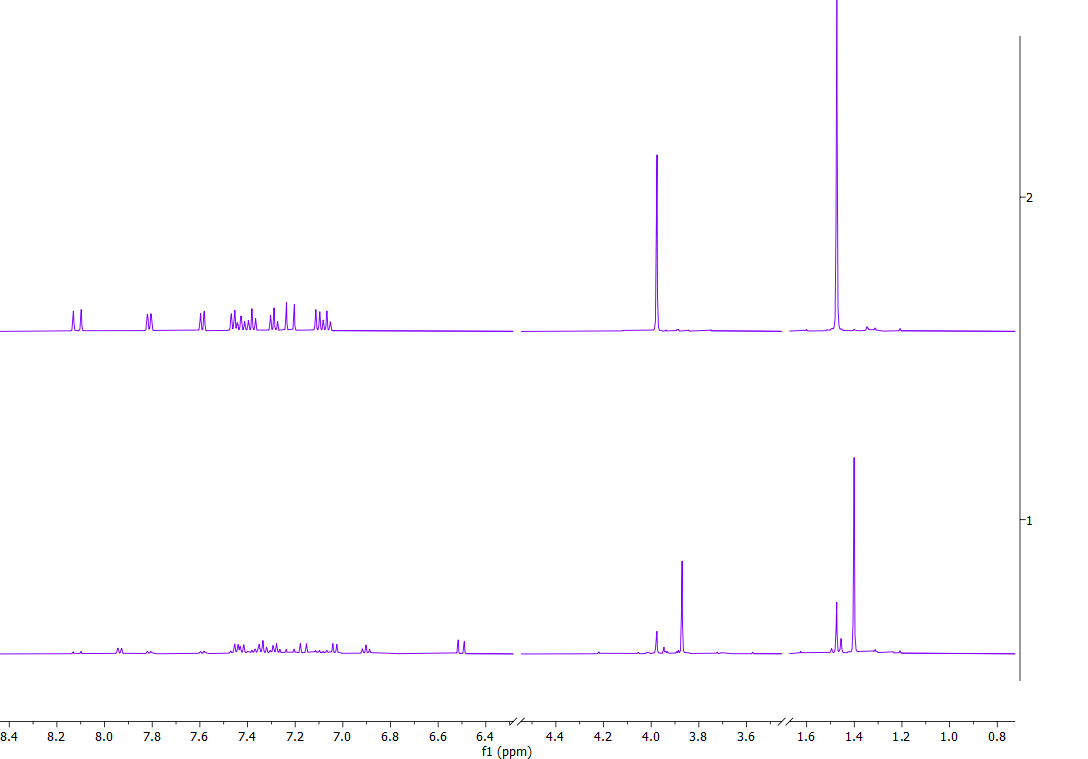


Figure S23: ^1^H-NMR spectra of 1g in CD_3_CN in the dark (purple) and at the PSS (cyan)


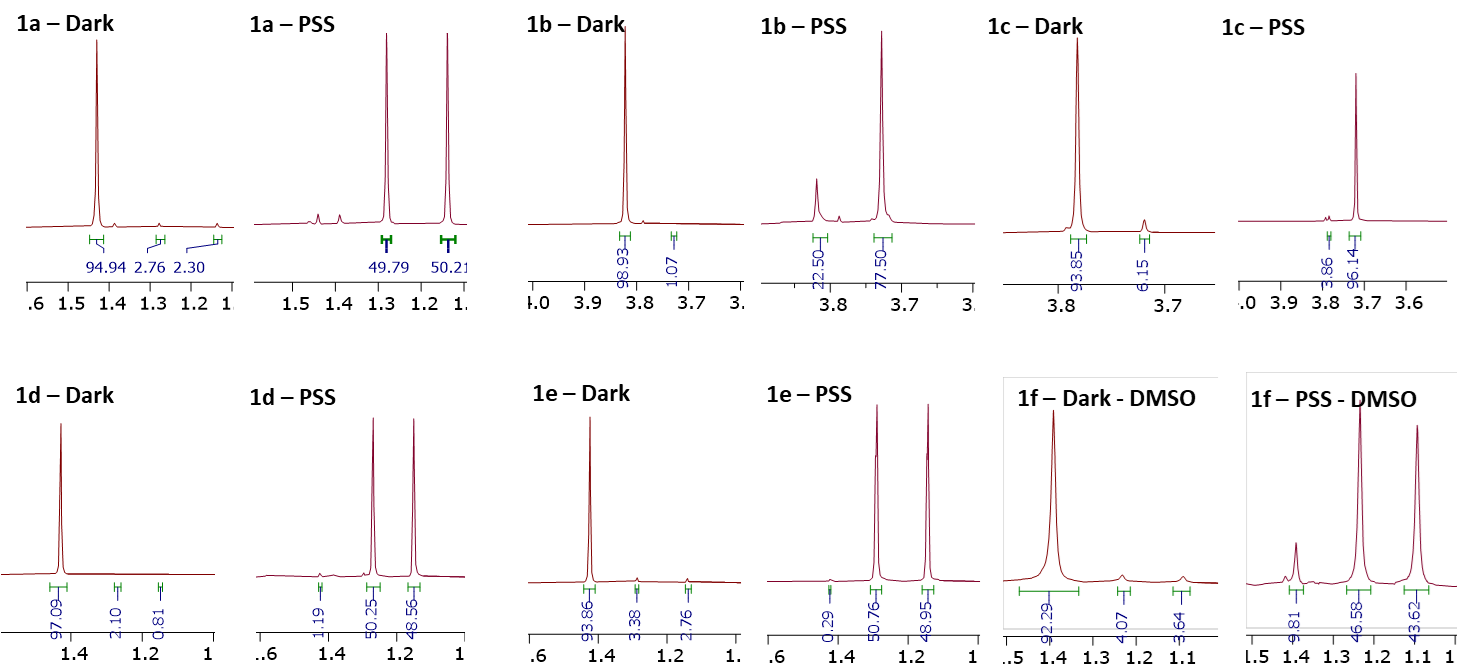


Figure S24: part of ^1^H-NMR spectra of 1a-f in CD_3_CN in the dark and at the PSS with integrals of significant peaks for dark and pss ratio determination for the open and closed forms. CD_3_CN is used as solvent for 1a-f and DMSO-d6 for 1f

**4.3. ^19^F-NMR with in situ irradiation**


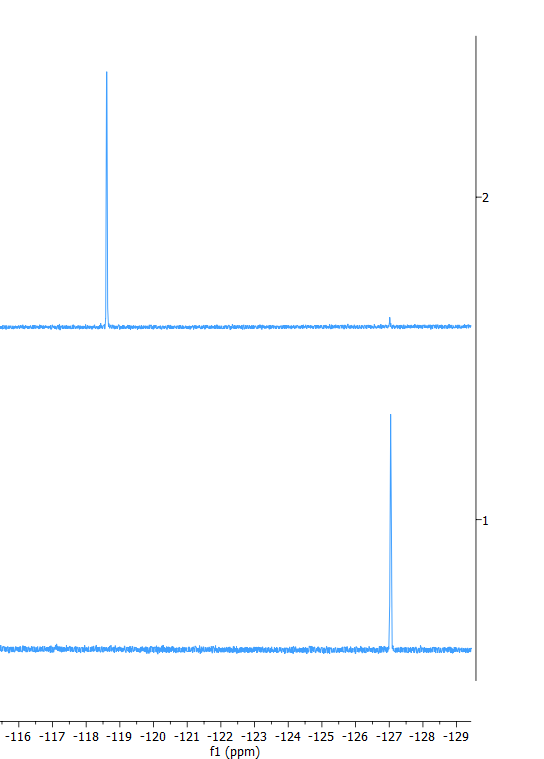

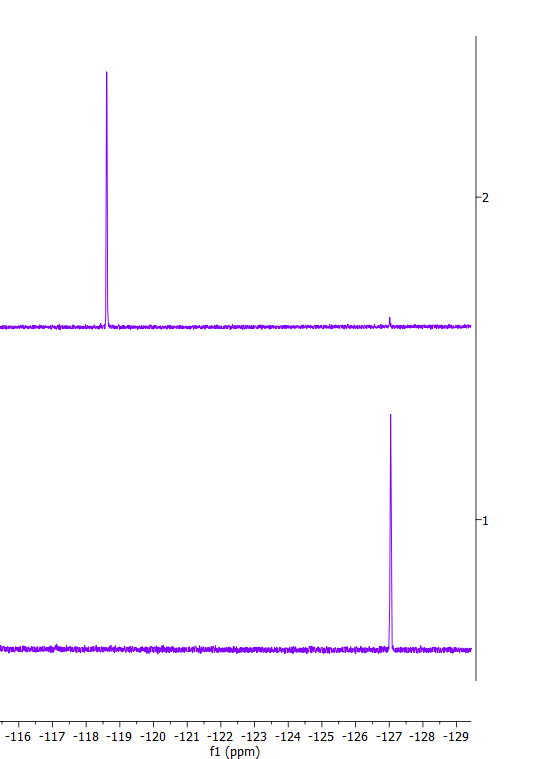


Figure S25: ^19^F-NMR spectra of 1d in CD_3_CN in the dark (purple) and at the PSS (cyan)


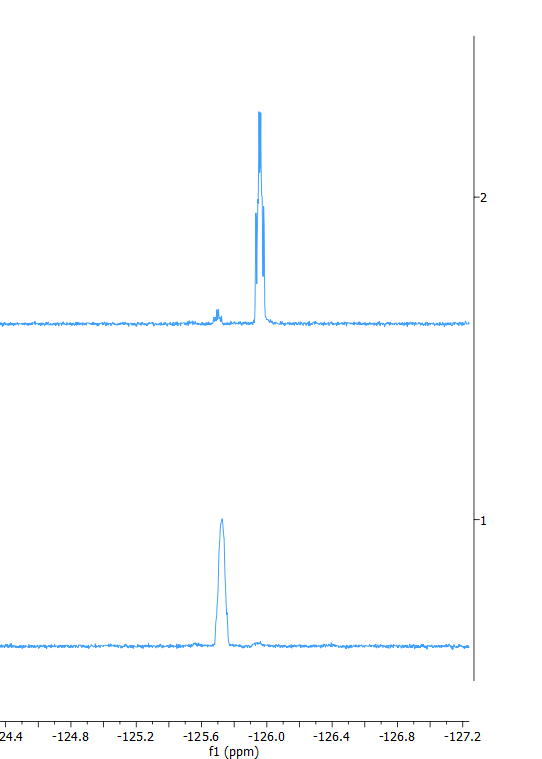

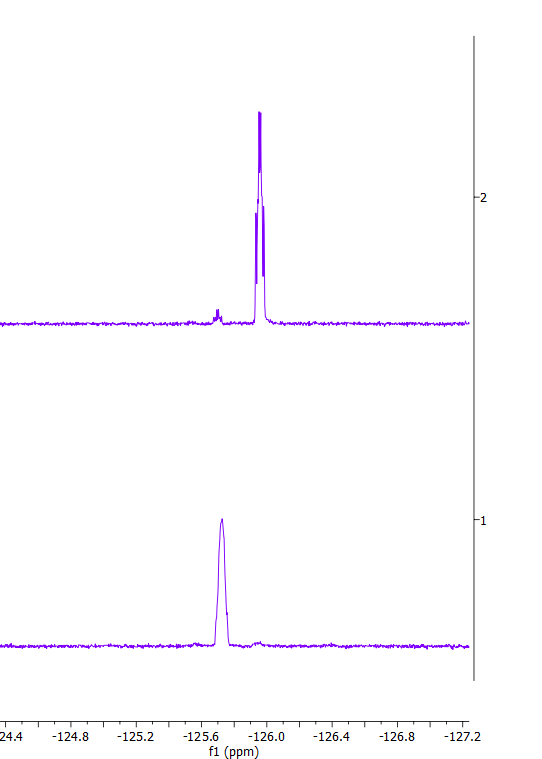


Figure S26: ^19^F-NMR spectra of 1e in CD_3_CN in the dark (purple) and at the PSS (cyan)

**5. UV-Vis studies**

**5.1. General procedure**

Samples were prepared from dilution of a stock solution in the same solvent unless specified differently (see section S5.3.), and measured within the next few hours. Measurements were performed at 293,15K unless differently specified. Solutions were made using spectroscopy or HPLC grade solvents.

Obtained spectra were analysed using the software Spectragryph, the baseline corrected for baseline drifting, and then plotted and analysed in Origin Software (from OriginLab).

An exponential equation was used of the form:

y = A1*exp(-x/t1) + y0

where A1 is a pre-exponential term, y0 is a constant and t1 is the time constant in s.

**5.2. Irradiation experiments**

**5.2.1. Photoisomerization kinetics**

**
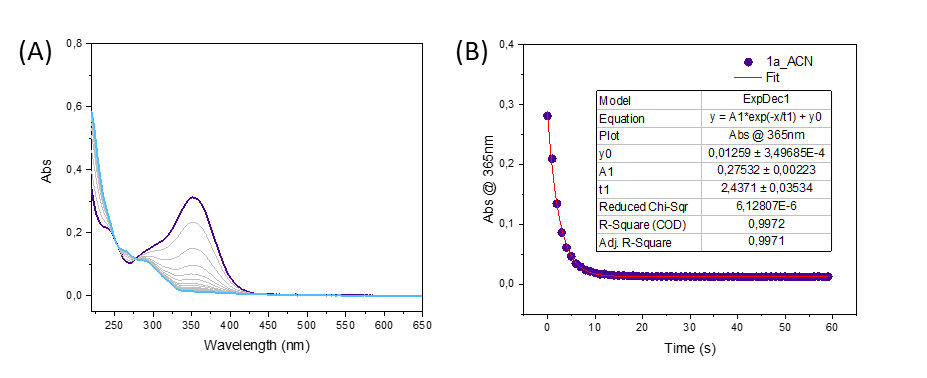
**

Figure S27: A) Change in the absorbance spectrum of 1a in CH_3_CN upon irradiation with 365 nm LED at 20°C. B) Change in absorbance at 365 nm during the irradiation of open 1a with a 365 nm LED at 20°C in CH_3_CN


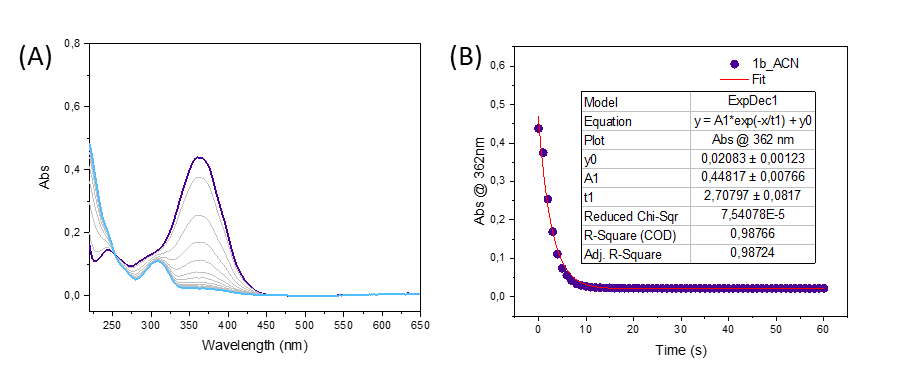


Figure S28: A) Change in the absorbance spectrum of 1b in CH_3_CN upon irradiation with 365 nm LED at 20°C. B) Change in absorbance at 365 nm during the irradiation of open 1b with a 365 nm LED at 20°C in CH_3_CN


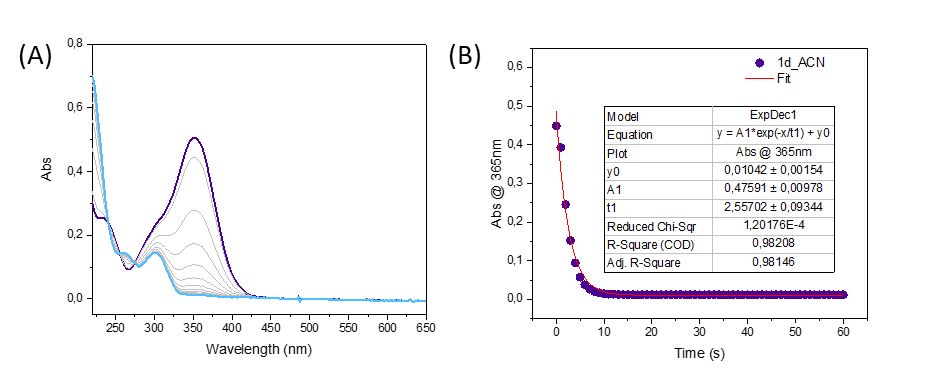

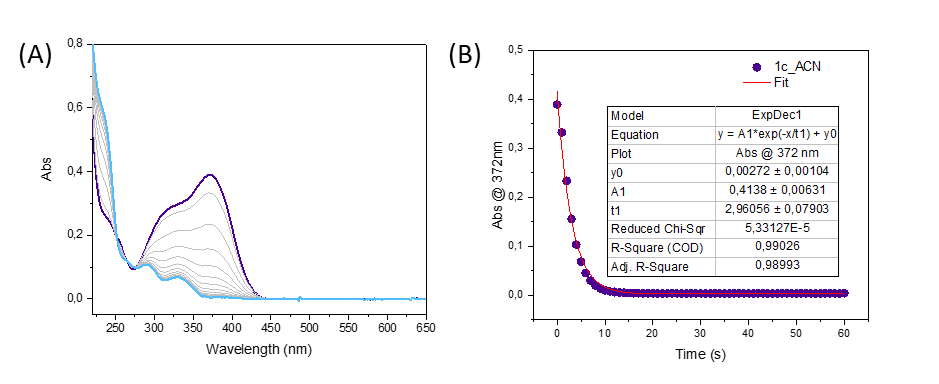
Figure S29: A) Change in the absorbance spectrum of 1c in CH_3_CN upon irradiation with 365 nm LED at 20°C. B) Change in absorbance at 365 nm during the irradiation of open 1c with a 365 nm LED at 20°C in CH_3_CN

Figure S30: A) Change in the absorbance spectrum of 1d in CH_3_CN upon irradiation with 365 nm LED at 20°C. B) Change in absorbance at 365 nm during the irradiation of open 1d with a 365 nm LED at 20°C in CH_3_CN

**
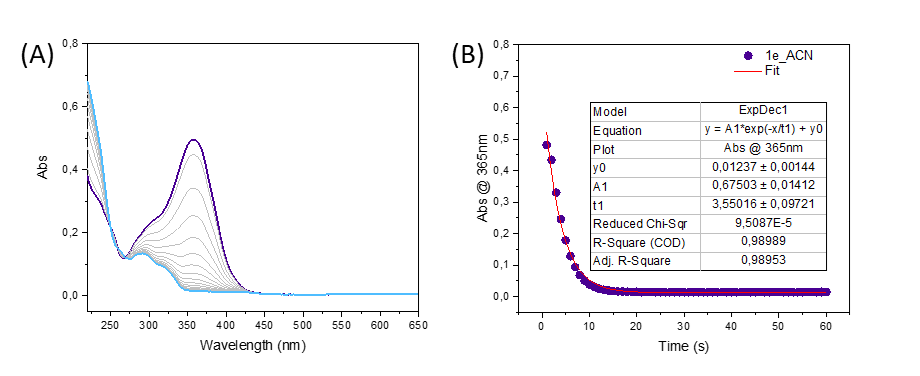
**

**
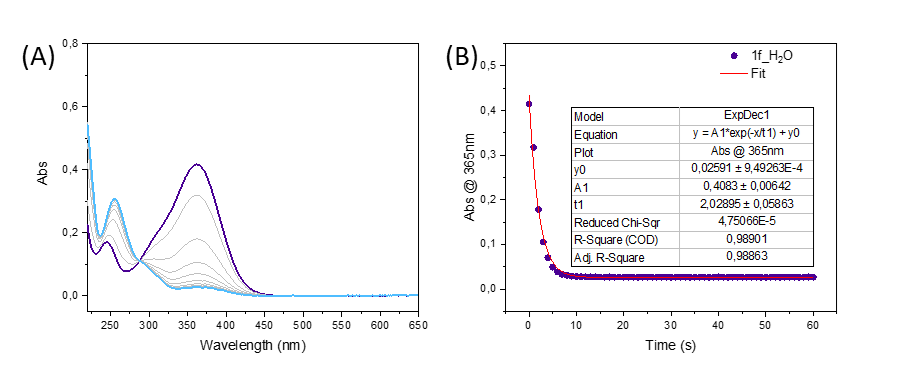
**Figure S31: A) Change in the absorbance spectrum of 1e in CH_3_CN upon irradiation with 365 nm LED at 20°C. B) Change in absorbance at 365 nm during the irradiation of open 1e with a 365 nm LED at 20°C in CH_3_CN

Figure S32: A) Change in the absorbance spectrum of 1f in Milliq Water upon irradiation with 365 nm LED at 20°C. B) Change in absorbance at 365 nm during the irradiation of open 1f with a 365 nm LED at 20°C in Milliq Water

**
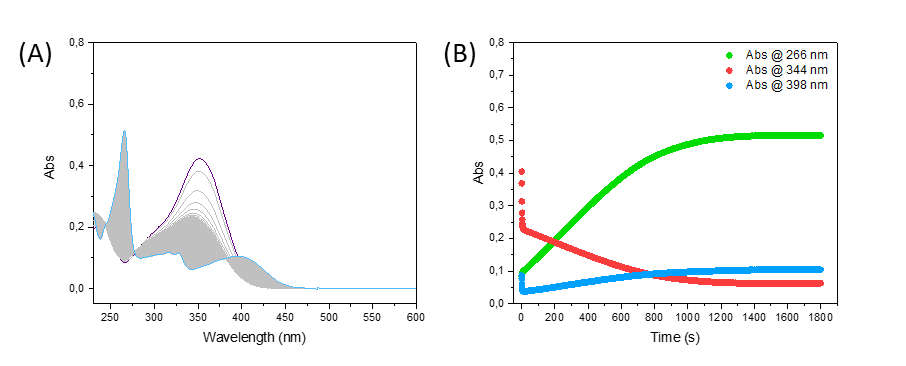
**

Figure S33: A) Change in the absorbance spectrum of 1g in CH_3_CN upon prolonged irradiation with 365 nm LED at 20°C in CH_3_CN. B) Change in absorbance at 266, 344, and 398 nm during the irradiation of open 1g with a 365 nm LED at 20°C in CH_3_CN

**5.2.2. Back relaxation kinetics**


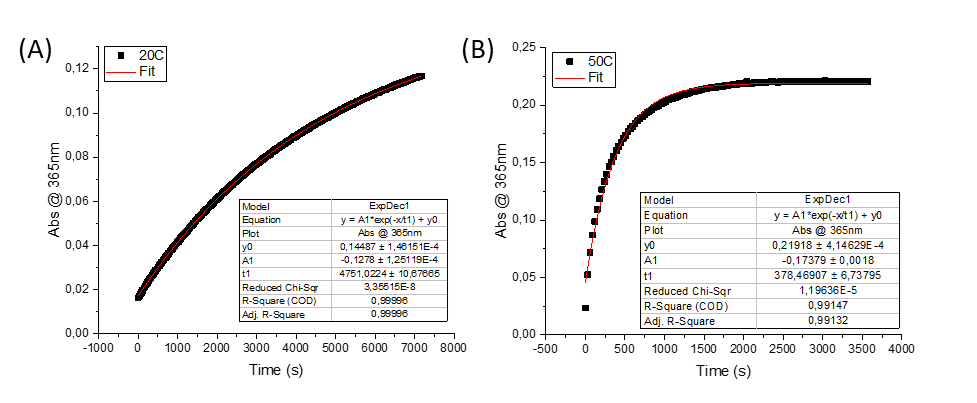


Figure S34: Change in absorbance of closed 1a at 365 nm over time at 20 °C (A) and 50 °C (B). The increase in absorbance is related to the thermal ring-opening process. Sample was irradiated using a 365 nm LED for 100 seconds in CH_3_CN before the measurement. Curves were fitted to determine the rate constants and the thermal half-life of the closed form.

Figure S35: Change in absorbance of closed 1b at the 362 nm over time at 20 °C (A) and 50 °C (B). The increase in absorbance is related to the thermal ring-opening process. Sample was irradiated using a 365 nm LED for 100 seconds
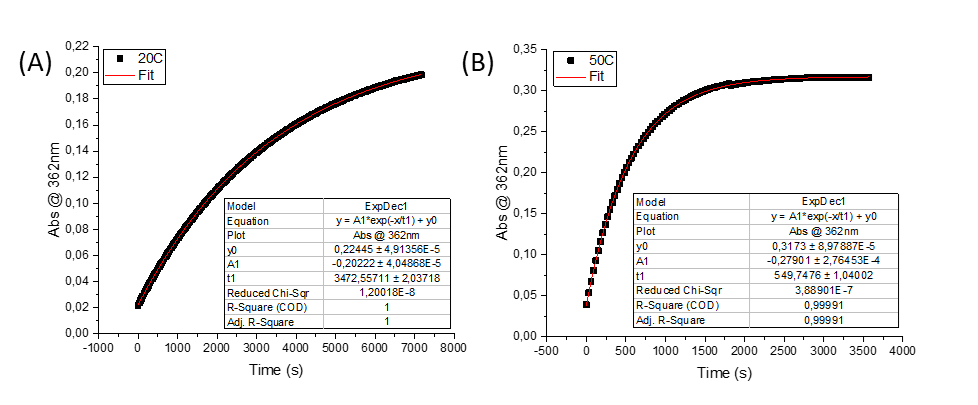
in CH_3_CN before the measurement. Curves were fitted to determine the rate constants and the thermal half-life of the closed form.


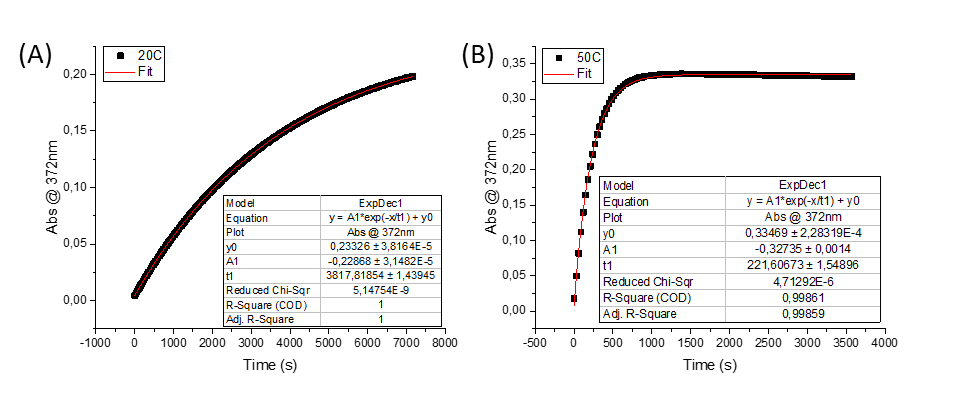


Figure S36: Change in absorbance of closed 1c at the 372 nm over time at 20 °C (A) and 50 °C (B). The increase in absorbance is related to the thermal ring-opening process. Sample was irradiated using a 365 nm LED for 100 seconds in CH_3_CN before the measurement. Curves were fitted to determine the rate constants and the thermal half-life of the closed form.


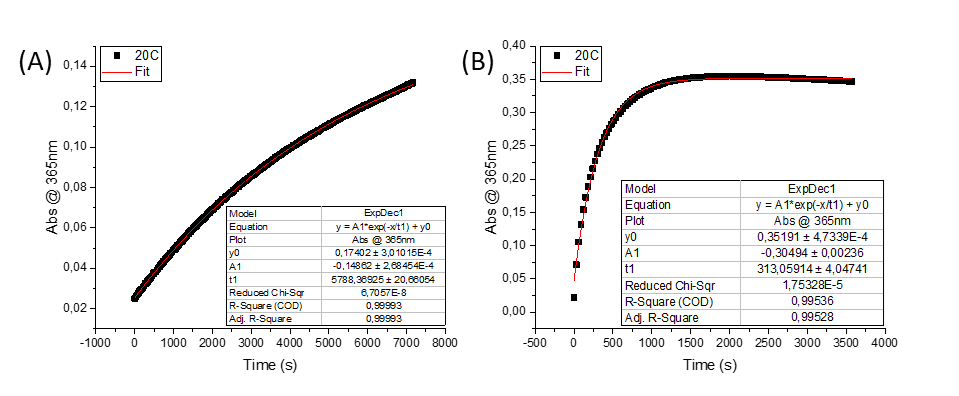


Figure S37: Change in absorbance of closed 1d at the 365 nm over time at 20 °C (A) and 50 °C (B). The increase in absorbance is related to the thermal ring-opening process. Sample was irradiated using a 365 nm LED for 100 seconds in CH_3_CN before the measurement. Curves were fitted to determine the rate constants and the thermal half-life of the closed form.


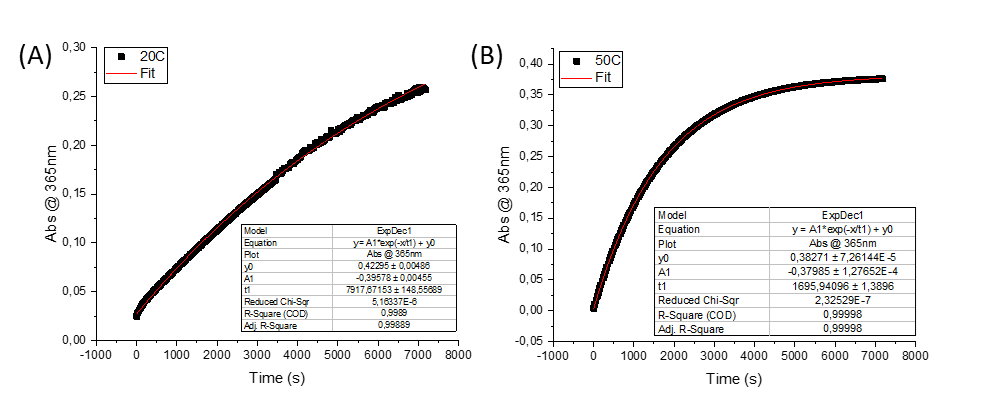


Figure S38: Change in absorbance of closed 1e at the 365 nm over time at 20 °C (A) and 50 °C (B). The increase in absorbance is related to the thermal ring-opening process. Sample was irradiated using a 365 nm LED for 100 seconds in CH_3_CN before the measurement. Curves were fitted to determine the rate constants and the thermal half-life of the closed form.


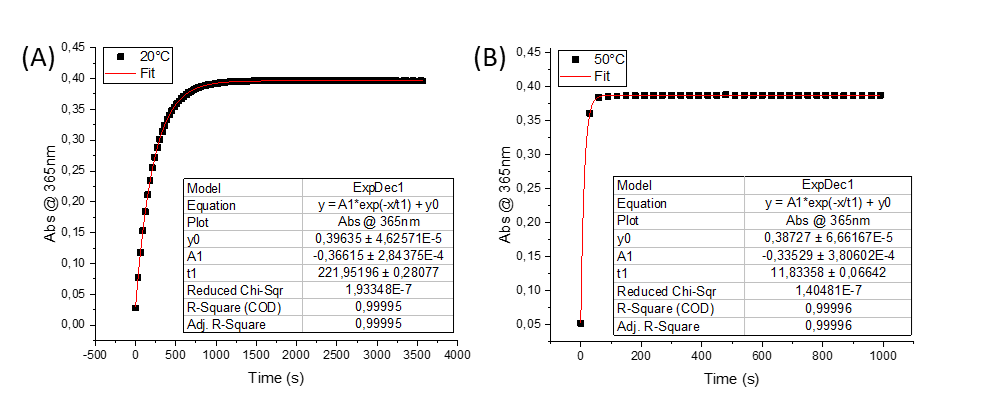


Figure S39: Change in absorbance of closed 1f at the 365 nm over time at 20 °C (A) and 50 °C (B). The increase in absorbance is related to the thermal ring-opening process. Sample was irradiated using a 365 nm LED for 100 seconds in Milliq Water before the measurement. Curves were fitted to determine the rate constants and the thermal half-life of the closed form.

(A)

(B)

Figure S40: Recorded absorbance of closed 1g in CH_3_CN at the 266 and 398 nm over time at 20 °C (A) and absorbance spectra over time (B). Sample was irradiated using a 365 nm LED for 1800 seconds in CH_3_CN before the measurement. No changes were detected.

**5.2.3. Photoisomerization kinetics in Methanol**


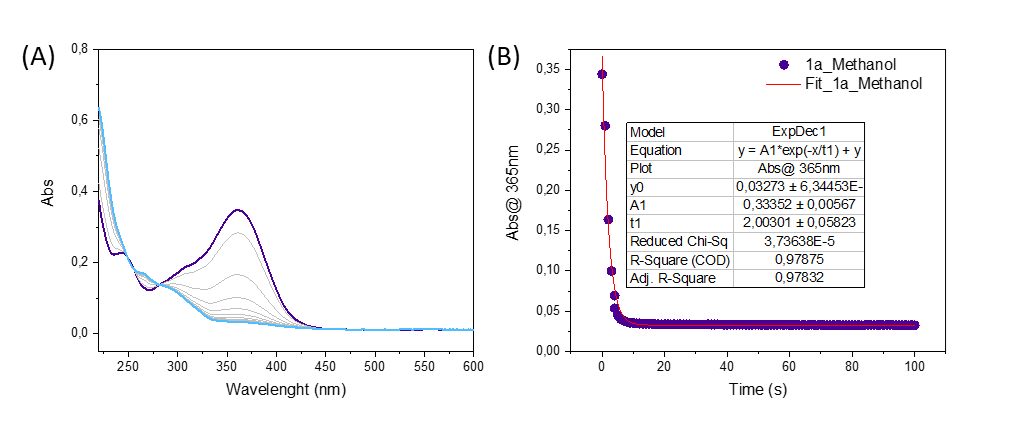


Figure S41: A) Change in the absorbance spectrum of 1a in MeOH upon irradiation with 365 nm LED at 20°C. B) Change in absorbance at 365 nm during the irradiation of open 1a with a 365 nm LED at 20°C in MeOH


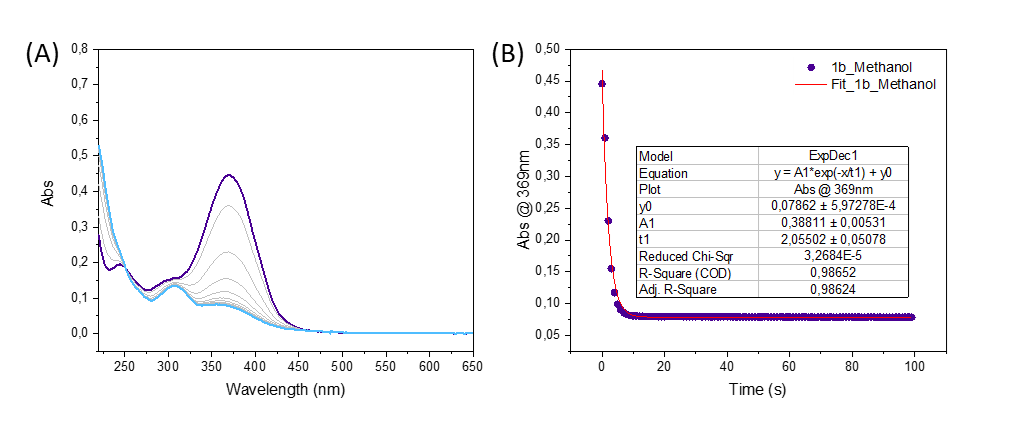


Figure S42: A) Change in the absorbance spectrum of 1b in MeOH upon irradiation with 365 nm LED at 20°C. B) Change in absorbance at 369 nm during the irradiation of open 1b with a 365 nm LED at 20°C in MeOH


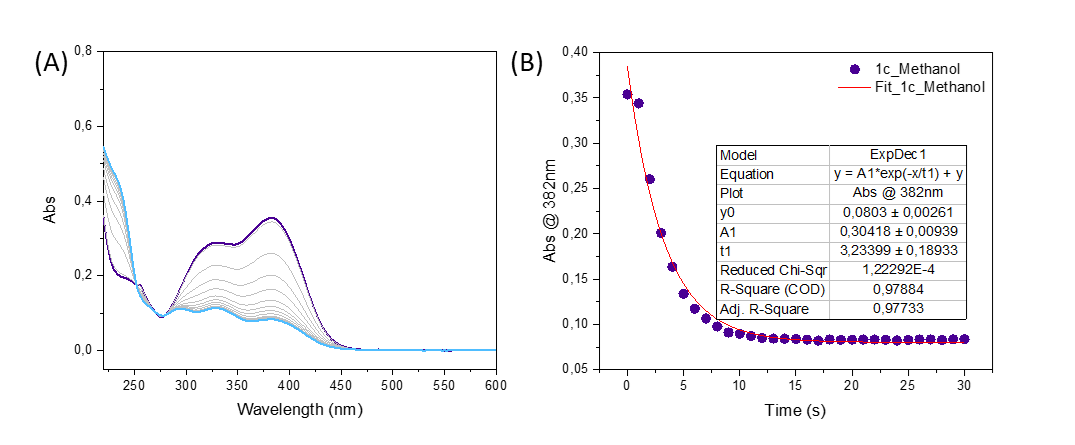


Figure S43: A) Change in the absorbance spectrum of 1c in MeOH upon irradiation with 365 nm LED at 20°C. B) Change in absorbance at 382 nm during the irradiation of open 1c with a 365 nm LED at 20°C in MeOH


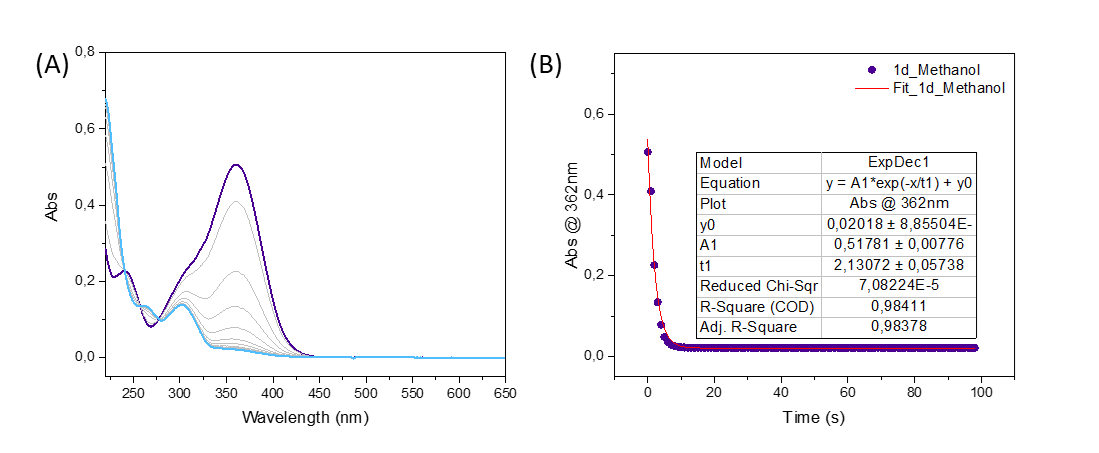


Figure S44: A) Change in the absorbance spectrum of 1d in MeOH upon irradiation with 365 nm LED at 20°C. B) Change in absorbance at 362 nm during the irradiation of open 1d with a 365 nm LED at 20°C in MeOH


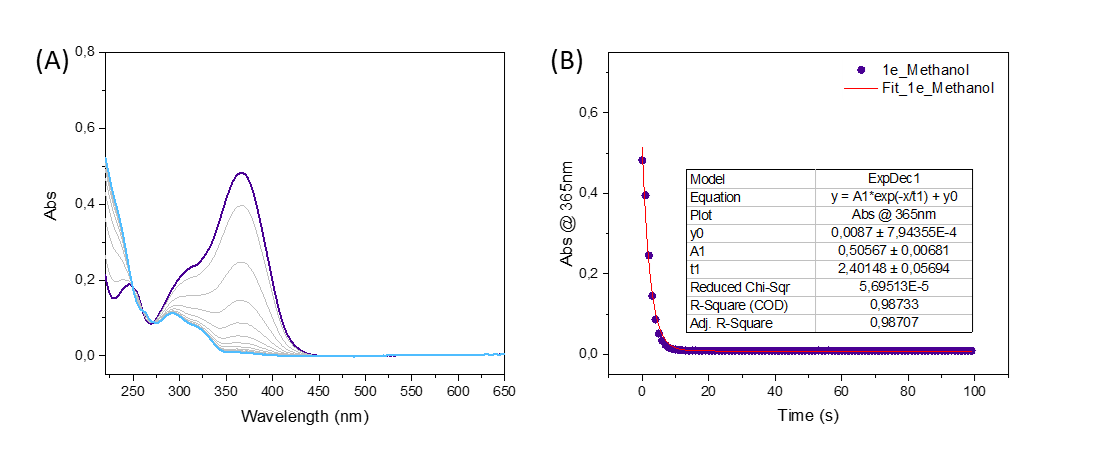
Figure S45: A) Change in the absorbance spectrum of 1e in MeOH upon irradiation with 365 nm LED at 20°C. B) Change in absorbance at 365 nm during the irradiation of open 1e with a 365 nm LED at 20°C in MeOH

**5.2.4. Back relaxation kinetics in Methanol**


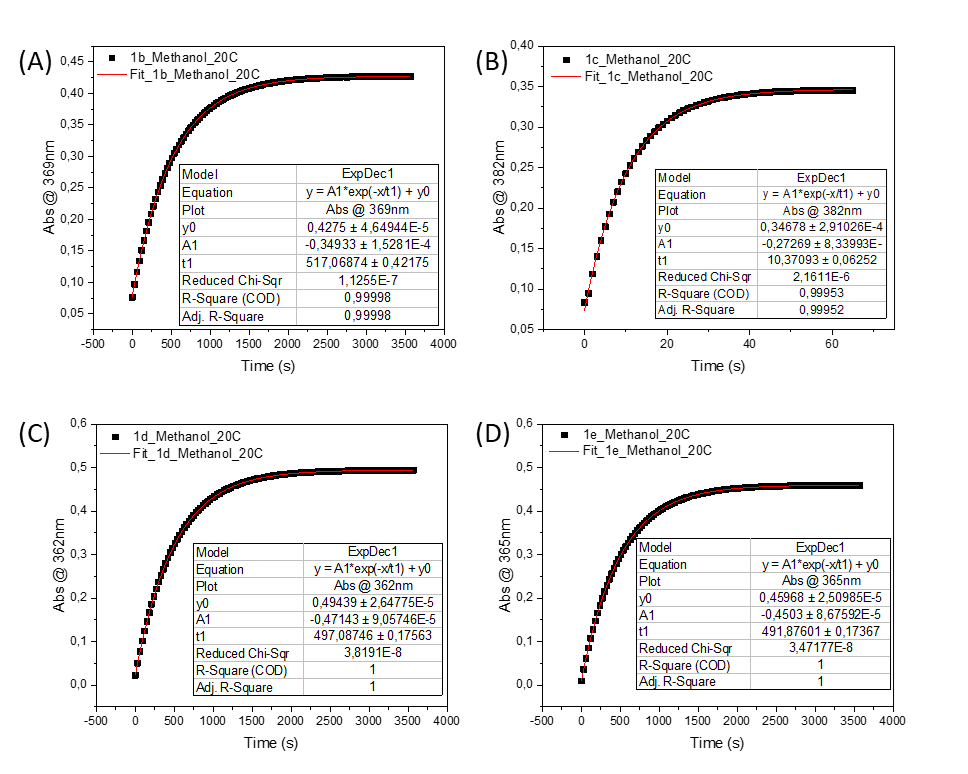


Figure S46: (A-D) Change in absorbance of closed 1b-e at the λmax over time at 20 °C in MeOH. The increase in absorbance is related to the thermal ring-opening process. Sample was irradiated using a 365 nm LED for 100 seconds in MeOH before the measurement. Curves were fitted to determine the rate constants and the thermal half-life of the closed form

**5.2.5. Comparison of dark spectra in acetonitrile and methanol**


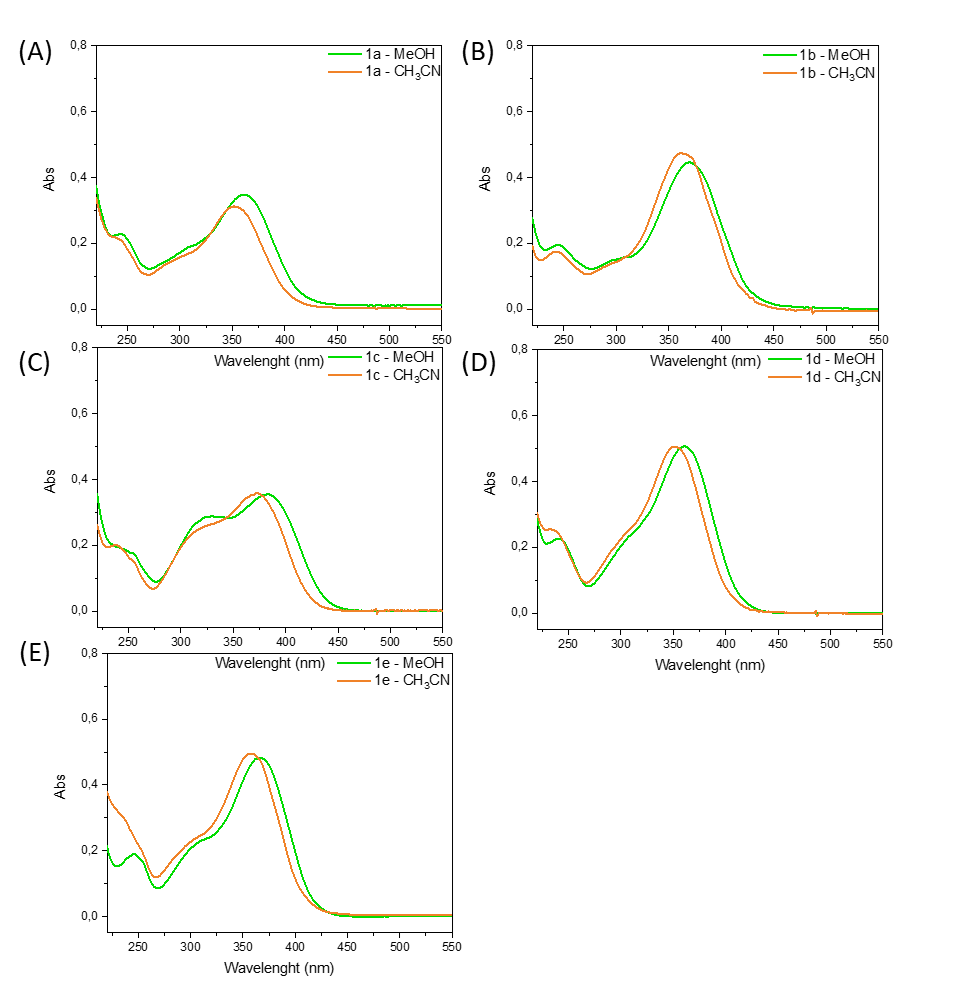


Figure S47: Overlap of the dark absorbance spectra of 1a-e in MeOH and CH_3_CN.

**5.2.6. Photoisomerization kinetics and back relaxation kinetics in solvents different from acetonitrile**


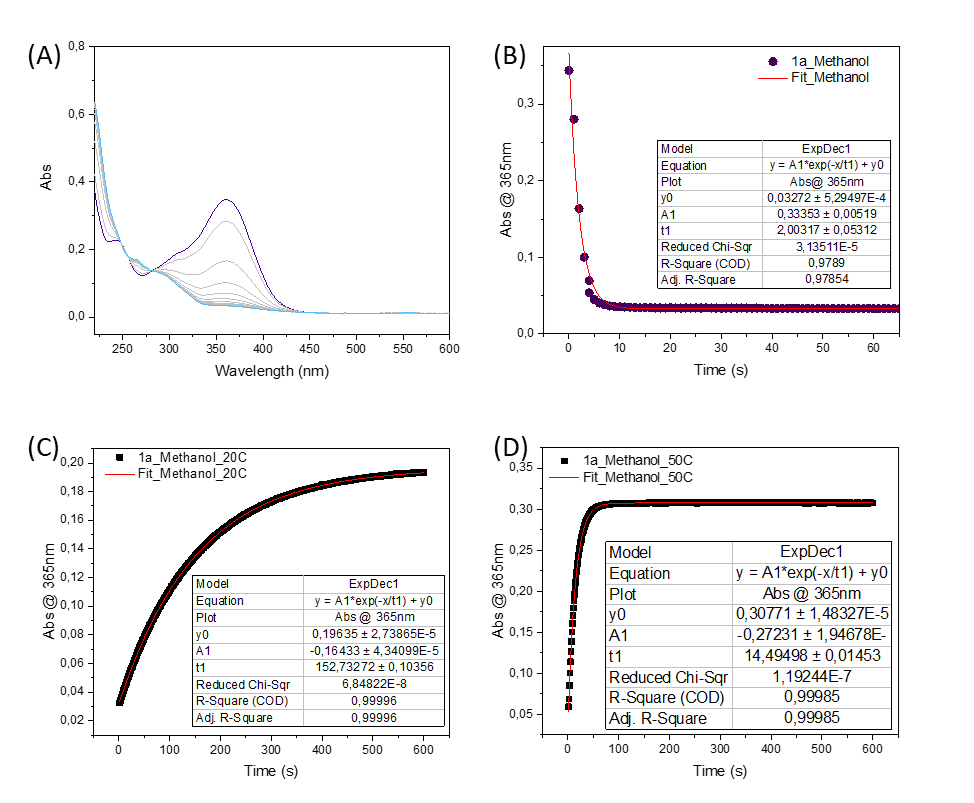


Figure S48: A) Change in the absorbance spectrum of 1a in MeOH upon irradiation with 365 nm LED at 20°C. B) Change in absorbance at 365 nm during the irradiation of open 1a with a 365 nm LED at 20°C in MeOH. C-D) Change in absorbance of closed 1a at 365 nm over time at 20 °C and 50 °C in MeOH. The increase in absorbance is related to the thermal ring-opening process. Sample was irradiated using a 365 nm LED for 100 seconds in MeOH before the measurement. Curves were fitted to determine the rate constants and the thermal half-life of the closed form.


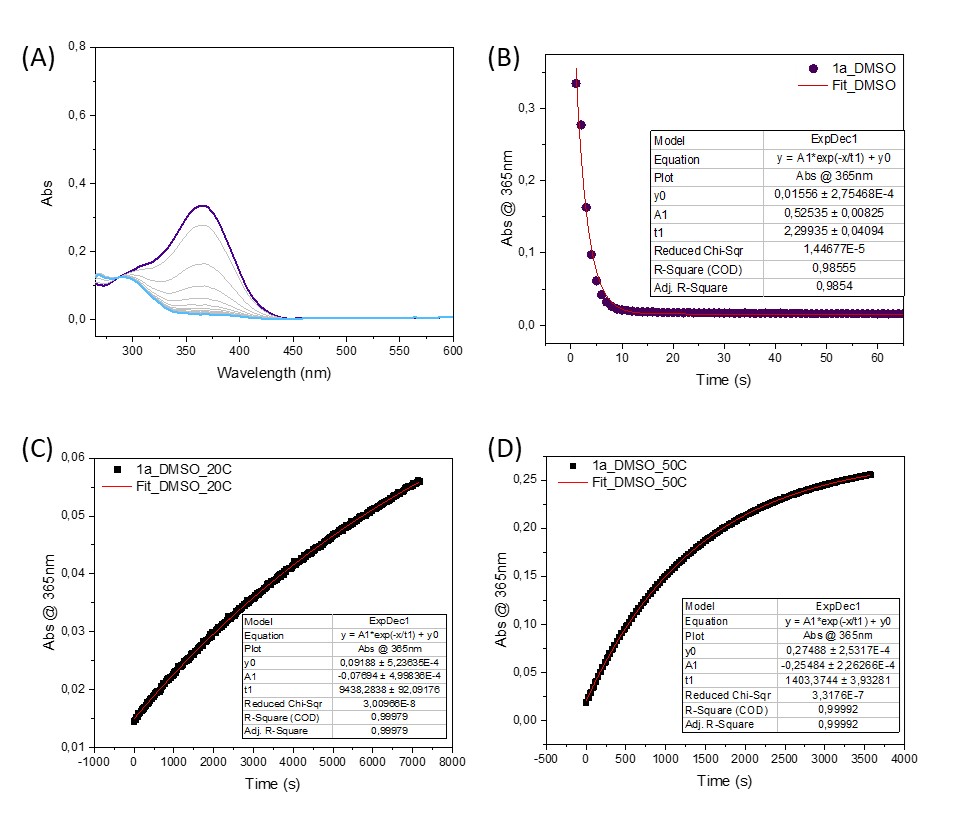


Figure S49: A) Change in the absorbance spectrum of 1a in DMSO upon irradiation with 365 nm LED at 20°C. B) Change in absorbance at 365 nm during the irradiation of open 1a with a 365 nm LED at 20°C in DMSO. C-D) Change in absorbance of closed 1a at 365 nm over time at 20 °C and 50 °C in DMSO. The increase in absorbance is related to the thermal ring-opening process. Sample was irradiated using a 365 nm LED for 100 seconds in DMSO before the measurement. Curves were fitted to determine the rate constants and the thermal half-life of the closed form.


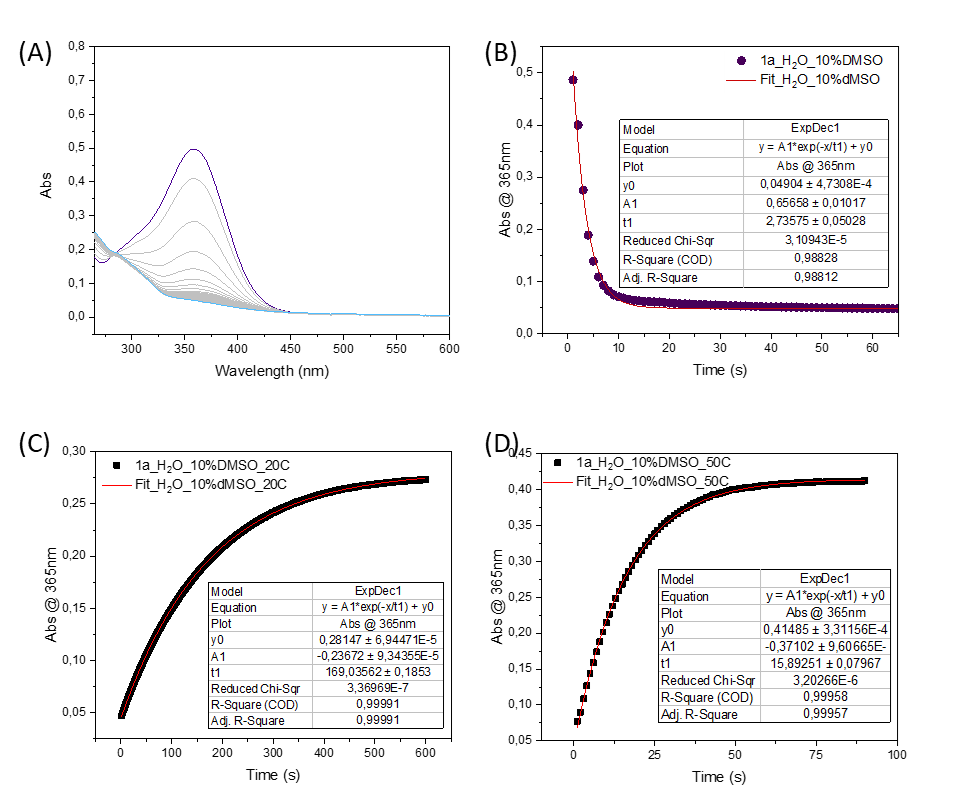


Figure S50: A) Change in the absorbance spectrum of 1a in Milliq Water:DMSO 9:1 mixture upon irradiation with 365 nm LED at 20°C. B) Change in absorbance at 365 nm during the irradiation of open 1a with a 365 nm LED at 20°C in Milliq Water:DMSO 9:1 mixture. C-D) Change in absorbance of closed 1a at 365 nm over time at 20 °C and 50 °C in Milliq Water:DMSO 9:1 mixture. The increase in absorbance is related to the thermal ring-opening process. Sample was irradiated using a 365 nm LED for 100 seconds in Milliq Water:DMSO 9:1 mixture before the measurement. Curves were fitted to determine the rate constants and the thermal half-life of the closed form.


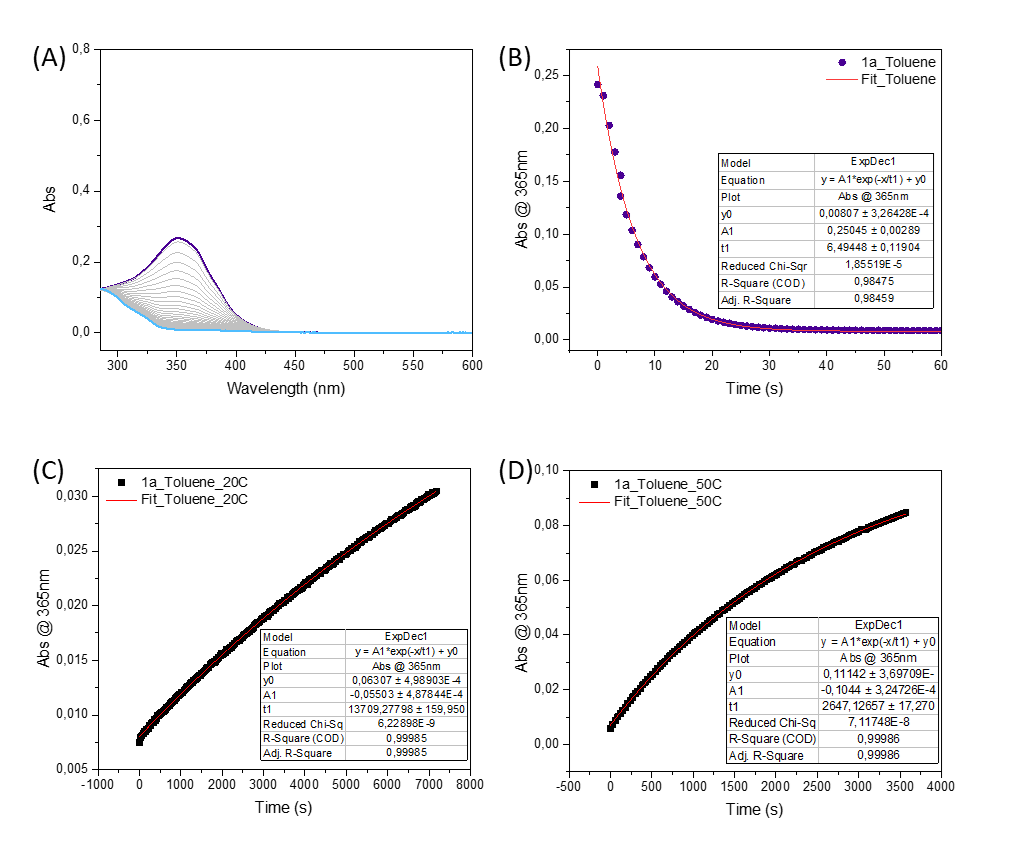


Figure S51: A) Change in the absorbance spectrum of 1a in Toluene upon irradiation with 365 nm LED at 20°C. B) Change in absorbance at 365 nm during the irradiation of open 1a with a 365 nm LED at 20°C in Toluene. C-D) Change in absorbance of closed 1a at 365 nm over time at 20 °C and 50 °C in Toluene. The increase in absorbance is related to the thermal ring-opening process. Sample was irradiated using a 365 nm LED for 100 seconds in Toluene before the measurement. Curves were fitted to determine the rate constants and the thermal half-life of the closed form.

**5.2.7. Fatigue resistance experiments**

Fatigue resistance experiments were conducted as follows: samples were irradiated for 60 seconds with a 365nm LED (1A) and subsequently kept in the dark for one hour. The cycles were repeated as many times as necessary.


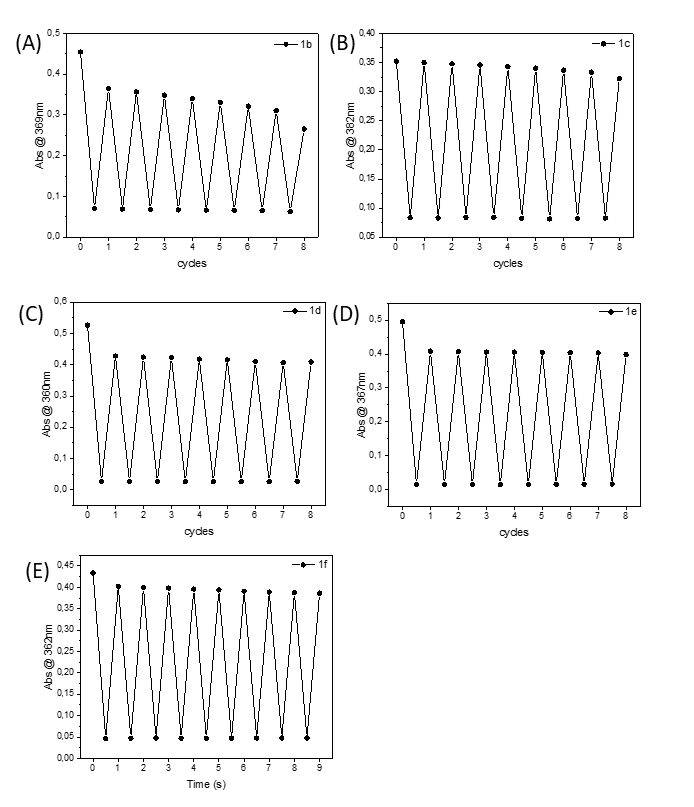


Figure S52: Fatigue resistance cycles of styrylcyanines. A) 1b in methanol 2x10^-5^ M B) 1c in methanol 2x10^-5^ M C) 1d in methanol 2x10^-5^ M D) 1e in methanol 2x10^-5^ M E) 1f in milli q water 2x10^-5^ M

**5.3. pKa determination experiments**

In order to determine the pKa values of the studied compounds, the methodology developed by Berton *et al*.^[6]^ for protonated merocyanines was applied. Buffer solutions were prepared according to the procedure of Berton *et al*.^[6]^

Samples were prepared as follow: 20 microliters of a stock solution of photoswitches 1a-f in DMSO at the concentration of 2*10^-3^M are placed in 1980 microliters of previously prepared buffer at the desired pH, stirred for a few seconds and measured immediately on a Agilent 8453UV-Vis Diode Array System, equipped with a Quantum Northwest Peltier controller in 10 mm quartz cuvettes. To determine the pKa at the photo stationary state, the samples were irradiated with a 365 nm LED until no changes in the absorbance spectra were recorded, and that spectrum was taken to determine the pKa^hv^. To determine the pKa values in the dark and at the PSS, the absorbance values corresponding to the protonated photoswitches absorbance peak were plotted against the pH. For pKa of the dark state, wavelength characteristic for protonated styryl cyanines were chosen, and the obtained sigmoidal curves were fitted using a Boltzmann-like equation on Origin software.

For further details on the used equations and parameters, we refer to previous works from Berton *et al.^[6]^* and Wimberger *et al.*^[7]^

**5.3.1. pKa titration of the dark state**


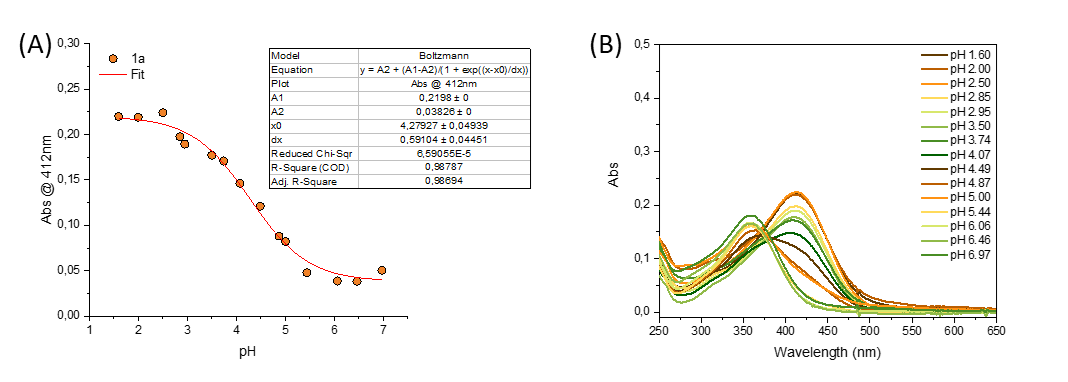
Figure S53: A) Change in the absorbance of 1a upon changing of pH of the solution (1% DMSO in water). Curves were fitted to determine the pKa in the dark state. B) Change in the absorbance spectra of 1a upon changing the pH of the solution (1% DMSO in water)


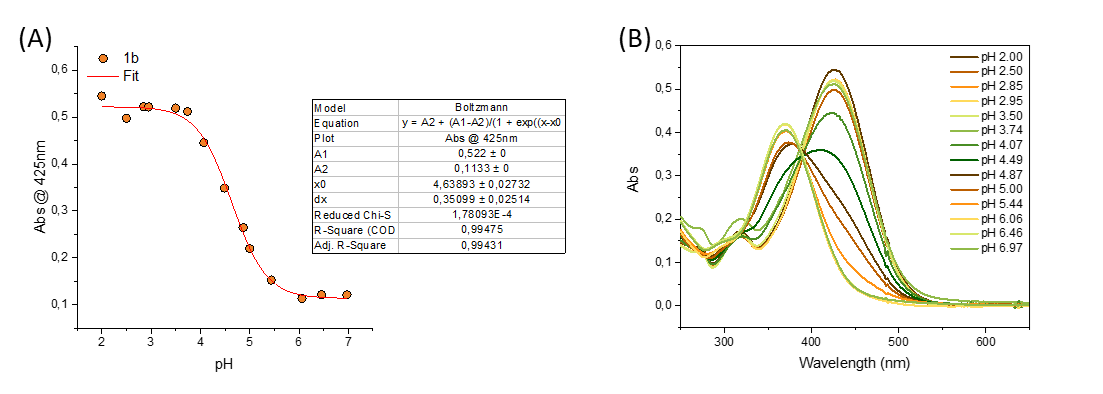
Figure S54: A) Change in the absorbance of 1b upon changing of pH of the solution (1% DMSO in water). Curves were fitted to determine the pKa in the dark state. B) Change in the absorbance spectra of 1b upon changing the pH of the solution (1% DMSO in water)


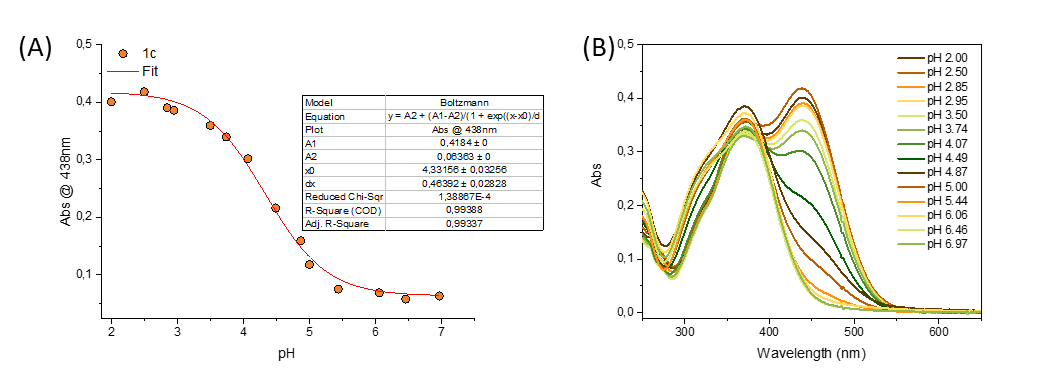


Figure S55: A) Change in the absorbance of 1c upon changing of pH of the solution (1% DMSO in water). Curves were fitted to determine the pKa in the dark state. B) Change in the absorbance spectra of 1c upon changing the pH of the solution (1% DMSO in water)


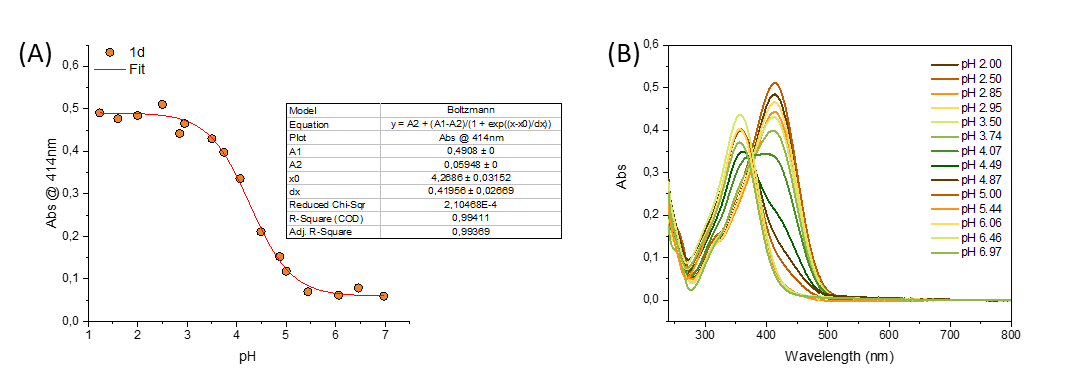


Figure S56: A) Change in the absorbance of 1d upon changing of pH of the solution (1% DMSO in water). Curves were fitted to determine the pKa in the dark state. B) Change in the absorbance spectra of 1d upon changing the pH of the solution (1% DMSO in water)


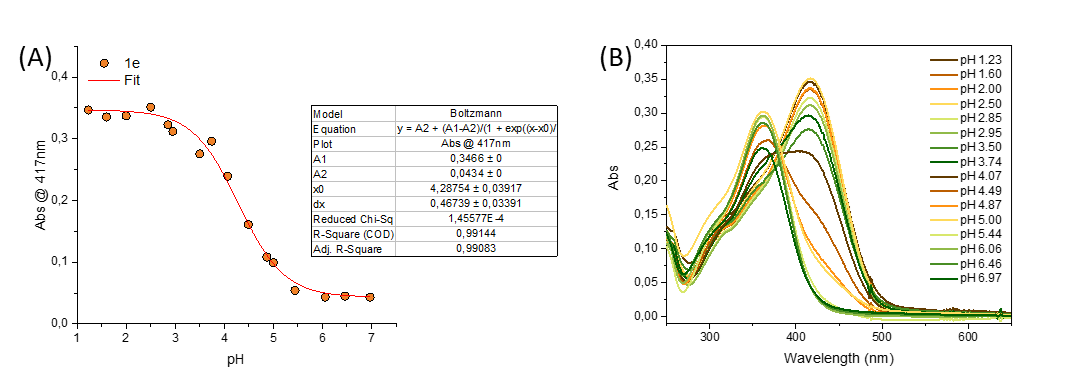


Figure S57: A) Change in the absorbance of 1e upon changing of pH of the solution (1% DMSO in water). Curves were fitted to determine the pKa in the dark state. B) Change in the absorbance spectra of 1e upon changing the pH of the solution (1% DMSO in water)


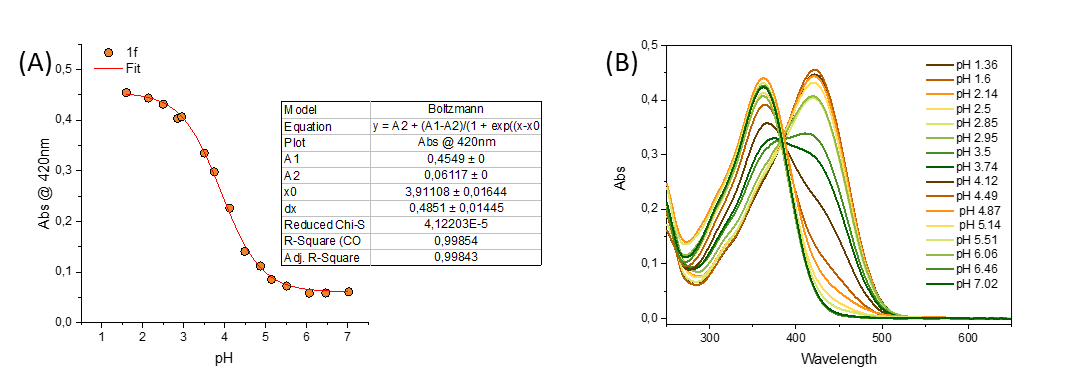


Figure S58: A) Change in the absorbance of 1f upon changing of pH of the solution (1% DMSO in water). Curves were fitted to determine the pKa in the dark state. B) Change in the absorbance spectra of 1f upon changing the pH of the solution (1% DMSO in water)

**5.3.2. pKa titration of the photostationary state**


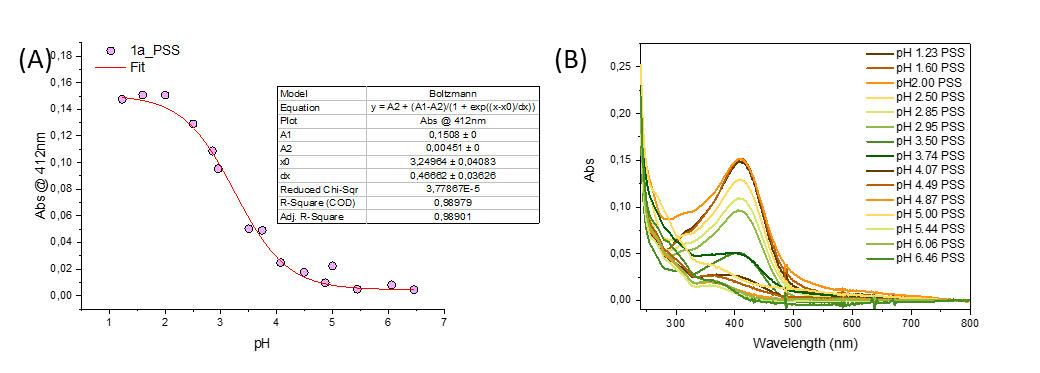


Figure S59: A) Change in the absorbance of closed 1a (PSS generated by irradiation with 365nm lamp) upon changing of pH of the solution (1% DMSO in water). Curves were fitted to determine the pKa in the PSS. B) Change in the absorbance spectra of closed 1a (PSS generated by irradiation with 365nm LED) upon changing the pH of the solution (1% DMSO in water)


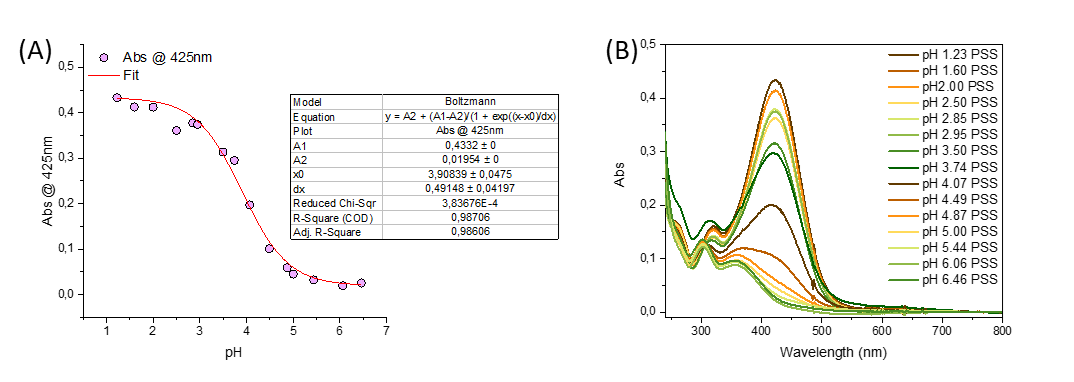


Figure S60: A) Change in the absorbance of closed 1b (PSS generated by irradiation with 365nm lamp) upon changing of pH of the solution (1% DMSO in water). Curves were fitted to determine the pKa in the PSS. B) Change in the absorbance spectra of closed 1b (PSS generated by irradiation with 365nm LED) upon changing the pH of the solution (1% DMSO in water)


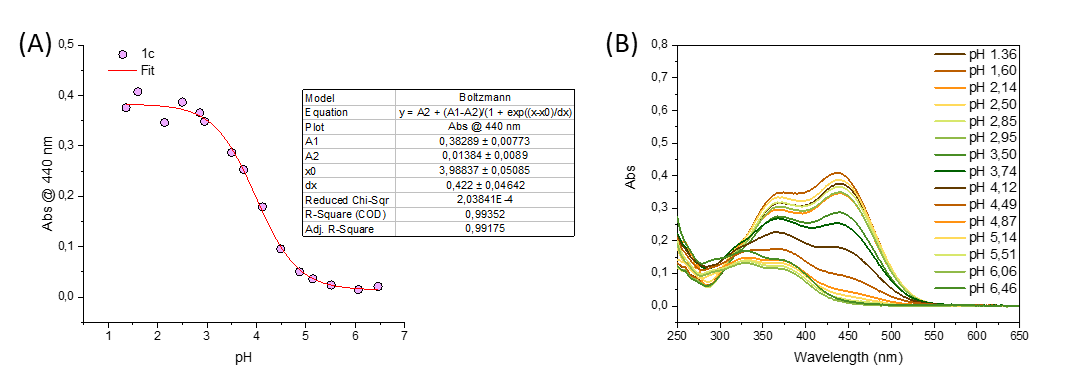


Figure S61: A) Change in the absorbance of closed 1c (PSS generated by irradiation with 365nm lamp) upon changing of pH of the solution (1% DMSO in water). Curves were fitted to determine the pKa in the PSS. B) Change in the absorbance spectra of closed 1c (PSS generated by irradiation with 365nm LED) upon changing the pH of the solution (1% DMSO in water)


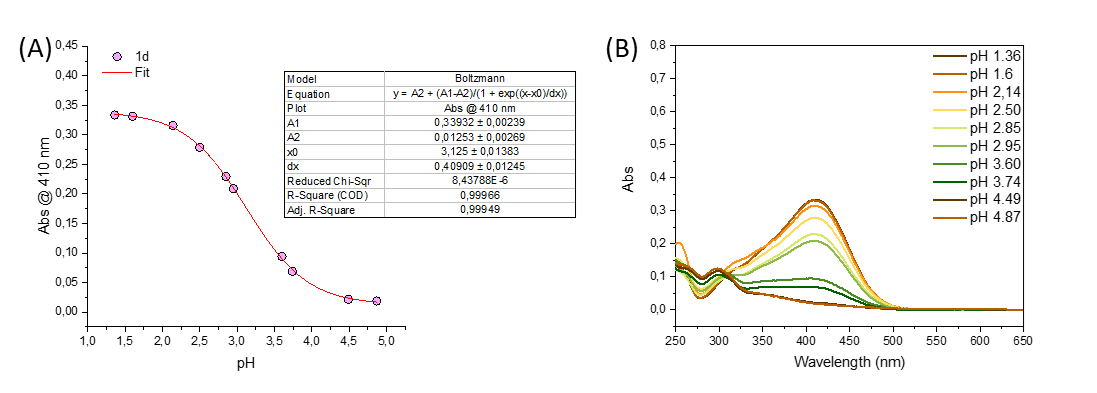


Figure S62: A) Change in the absorbance of closed 1d (PSS generated by irradiation with 365nm lamp) upon changing of pH of the solution (1% DMSO in water). Curves were fitted to determine the pKa in the PSS. B) Change in the absorbance spectra of closed 1d (PSS generated by irradiation with 365nm LED) upon changing the pH of the solution (1% DMSO in water)


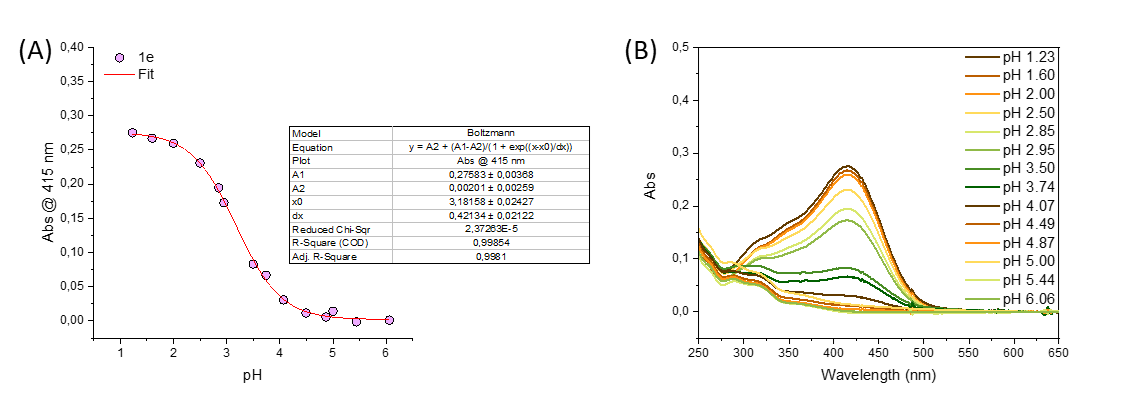


Figure S63: A) Change in the absorbance of closed 1e (PSS generated by irradiation with 365nm lamp) upon changing of pH of the solution (1% DMSO in water). Curves were fitted to determine the pKa in the PSS. B) Change in the absorbance spectra of closed 1e (PSS generated by irradiation with 365nm LED) upon changing the pH of the solution (1% DMSO in water)


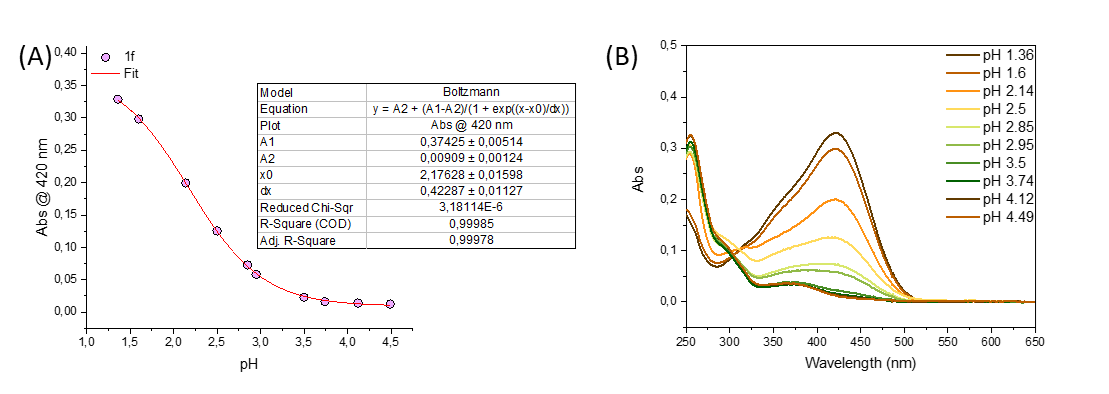


Figure S64: A) Change in the absorbance of closed 1f (PSS generated by irradiation with 365nm lamp) upon changing of pH of the solution (1% DMSO in water). Curves were fitted to determine the pKa in the PSS. B) Change in the absorbance spectra of closed 1f (PSS generated by irradiation with 365nm LED) upon changing the pH of the solution (1% DMSO in water)

**5.4.2.** **Photoisomerization Quantum Yield of 1a-f**

All the switches were irradiated using a 365 nm LED from Thorlabs (M365FP1). The photon flux of the LED was determined following the procedure from literature using potassium ferrioxalate and was 2,93E-05 mE/s.^[8]^

Figure S65: Determination of the photon flux of 365 nm LED
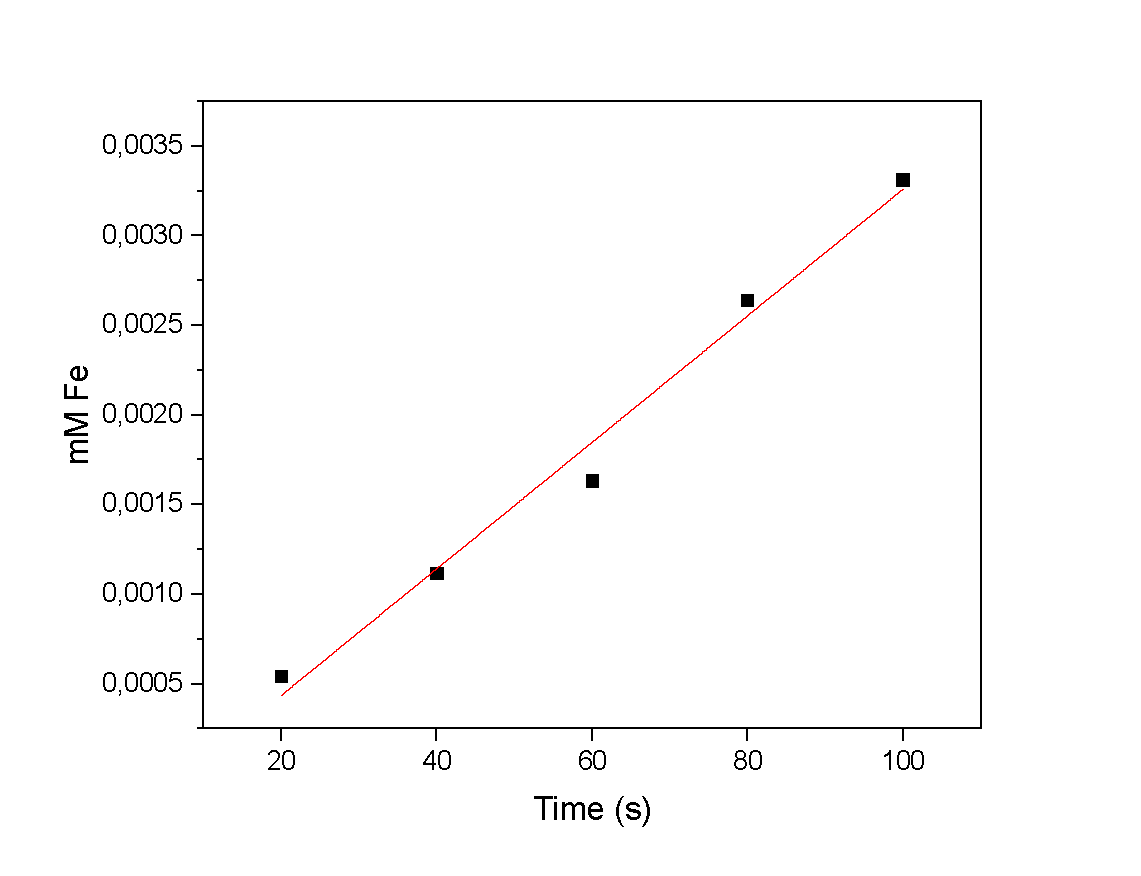


The concentration of 1a-f was determined using the Beer-Lambert law. To do so, absorbance values at 400 nm (for 1a), 405 nm (for 1d and 1e), and 420 nm (for 1b,c,f) and the extinction coefficient at the same wavelengths were used. The experiments were carried out in HPLC grade acetonitrile for 1a-e and milliq water for 1f. The change of the concentration of 1a-f during irradiation at 365 nm was linear and from the slope of the linear fit the quantum yield has been calculated.^[9]^

The photoisomerization quantum yield was determined using the found photon flux following the (Regime 1: total absorption) procedure from literature, keeping the absorbance at the irradiation wavelength above 2 over the course of the measurement.^[8]^

To determine the photoisomerization QY, the following equation has been used:^[9]^

$$\Phi=\frac{\Delta C}{\Delta t} *\frac{V}{I} = \mathrm{slope} *\frac{V}{I}$$

Where V= volume (L) and I= photon flux (mol/s).

(A)

(C)

(B)

Figure S66: (A-B) concentration of 1a over time during irradiation at 365nm (0.3A) in sample 1 and sample 2. Curves were fitted to determine the photoisomerization quantum yield. (C) Concentration of 1a vs Absorbance at 400 nm curve to determine molar extinction coefficient at 400 nm. Curve was fitted to determine the slope.

(A)

(C)

(B)

Figure S67: (A-B) concentration of 1b over time during irradiation at 365nm (0.3A) in sample 1 and sample 2. Curves were fitted to determine the photoisomerization quantum yield. (C) Concentration of 1b vs Absorbance at 420 nm curve to determine molar extinction coefficient at 420 nm. Curve was fitted to determine the slope.

Figure S68: (A-B) concentration of 1c over time during irradiation at 365nm (0.3A) in sample 1 and sample 2. Curves were fitted to determine the photoisomerization quantum yield. (C) Concentration of 1c vs Absorbance at 420 nm curve to determine molar extinction coefficient at 420 nm. Curve was fitted to determine the slope.

(A)

(C)

(B)

(A)

(C)

(B)

Figure S69: (A-B) concentration of 1d over time during irradiation at 365nm (0.3A) in sample 1 and sample 2. Curves were fitted to determine the photoisomerization quantum yield. (C) Concentration of 1`d vs Absorbance at 405 nm curve to determine molar extinction coefficient at 405 nm. Curve was fitted to determine the slope.

(A)

(C)

(B)

Figure S70: (A-B) concentration of 1e over time during irradiation at 365nm (0.3A) in sample 1 and sample 2. Curves were fitted to determine the photoisomerization quantum yield. (C) Concentration of 1e vs Absorbance at 405 nm curve to determine molar extinction coefficient at 405 nm. Curve was fitted to determine the slope.

(A)

(C)

(B)

Figure S71: Figure S70: (A-B) concentration of 1f over time during irradiation at 365nm (0.3A) in sample 1 and sample 2. Curves were fitted to determine the photoisomerization quantum yield. (C) Concentration of 1fvs Absorbance at 420 nm curve to determine molar extinction coefficient at 420 nm. Curve was fitted to determine the slope.

**5.5. Molar extinction coefficients determination**


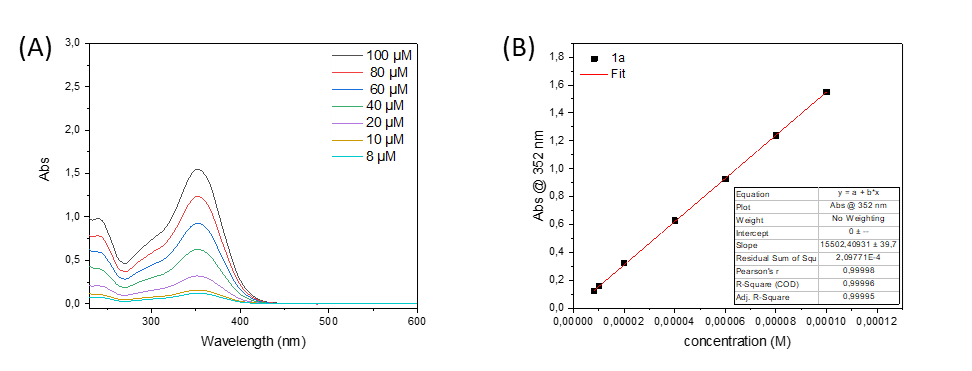


Figure S72: A) UV-Vis absorption spectra of 1a at different concentration in CH_3_CN at 20°C. B) Plot of the different absorption at the λ_max_ and fitting to determine the molar extinction coefficient


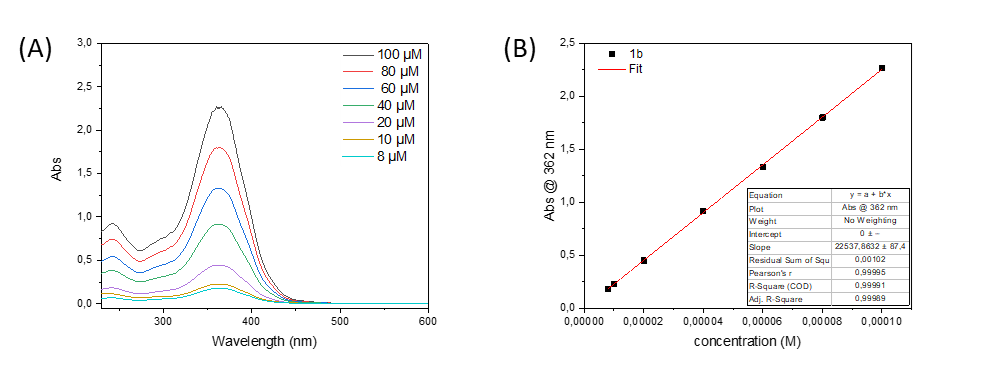


Figure S73: A) UV-Vis absorption spectra of 1b at different concentration in CH_3_CN at 20°C. B) Plot of the different absorption at the λ_max_ and fitting to determine the molar extinction coefficient


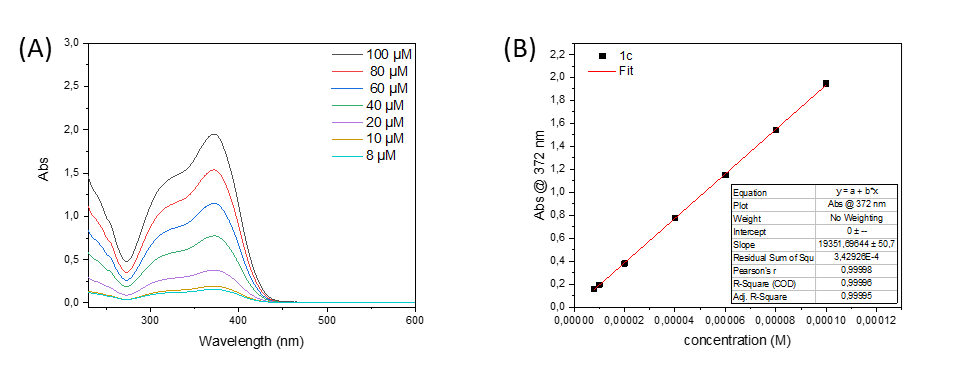


Figure S74: A) UV-Vis absorption spectra of 1c at different concentration in CH_3_CN at 20°C. B) Plot of the different absorption at the λ_max_ and fitting to determine the molar extinction coefficient


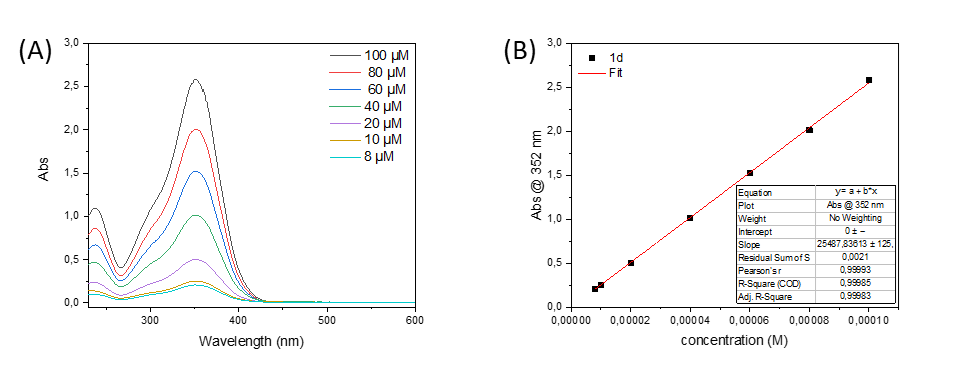


Figure S75: A) UV-Vis absorption spectra of 1d at different concentration in CH_3_CN at 20°C. B) Plot of the different absorption at the λ_max_ and fitting to determine the molar extinction coefficient


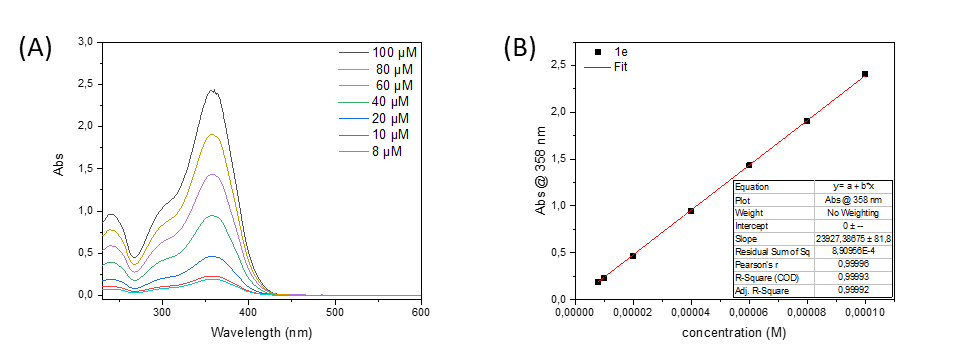


Figure S76: A) UV-Vis absorption spectra of 1e at different concentration in CH_3_CN at 20°C. B) Plot of the different absorption at the λ_max_ and fitting to determine the molar extinction coefficient


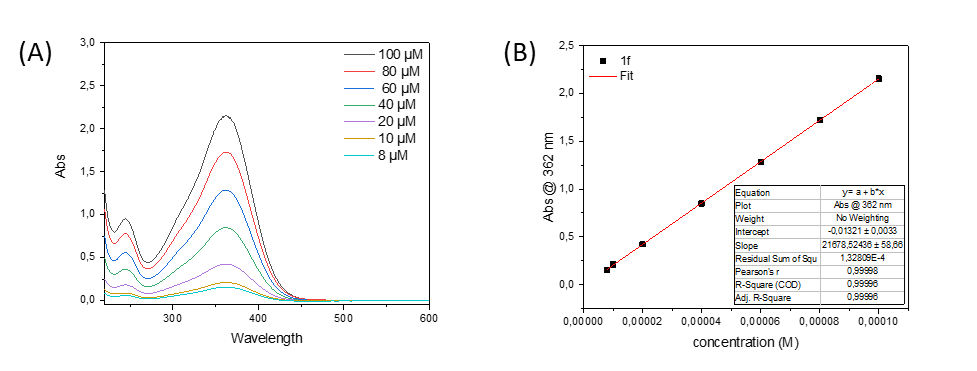


Figure S77: A) UV-Vis absorption spectra of 1f at different concentration in Milliq Water at 20°C. B) Plot of the different absorption at the λ_max_ and fitting to determine the molar extinction coefficient

**5.6. UV-Vis studies on polymeric systems**

**5.6.1. Solution UV-Vis absorbance spectroscopy**

Figure S78: UV-Vis absorbance spectrum of poly-4a in methanol (0.2 mg/ml)
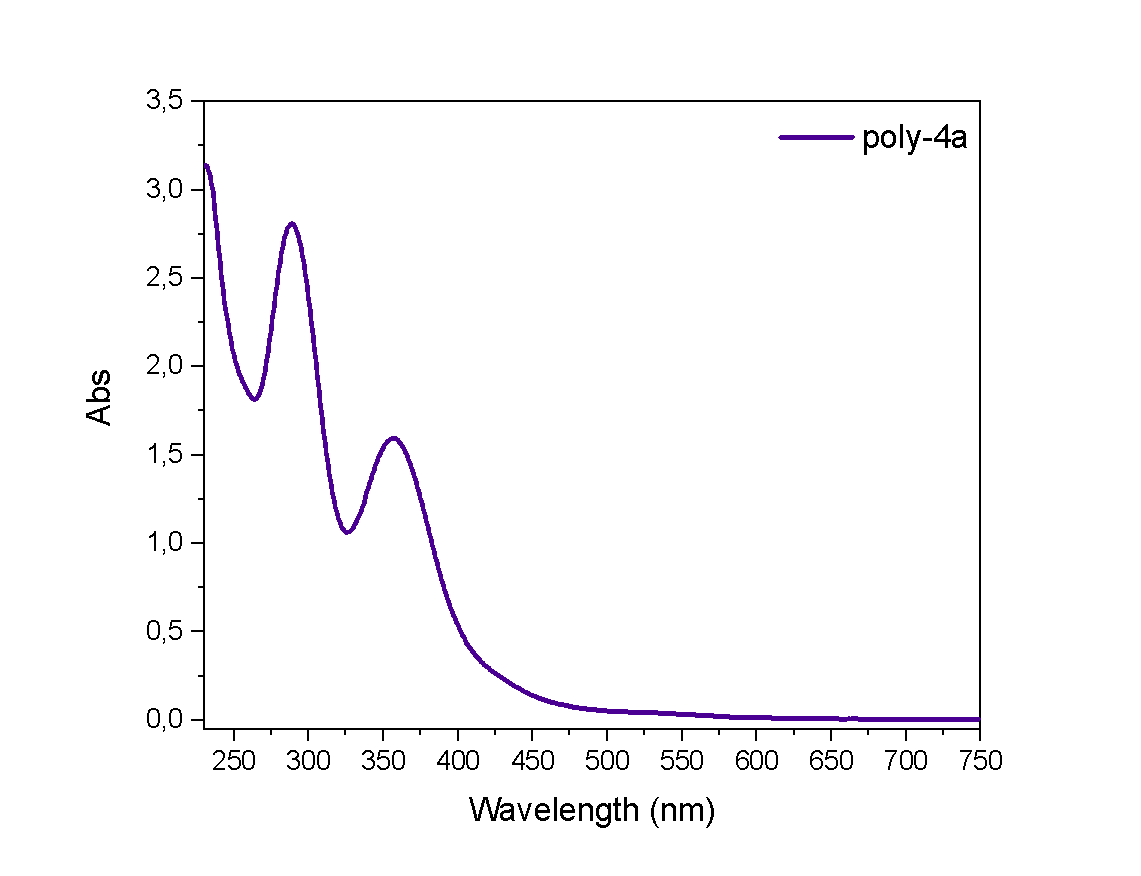


**5.6.2. Solid-state UV-Vis absorbance spectroscopy**

Samples were prepared as follows: 80 μl of a 8 mg/ml polymer solution containing from 2.5 up to 20 wt% of 1a were drop casted on glass slides at 50 °C. The samples were kept in the dark prior measurements. Irradiation was performed using a 455 nm LED (1A) from Thorlabs, directly placed on the sample tilted inside the spectrometer sample holder.


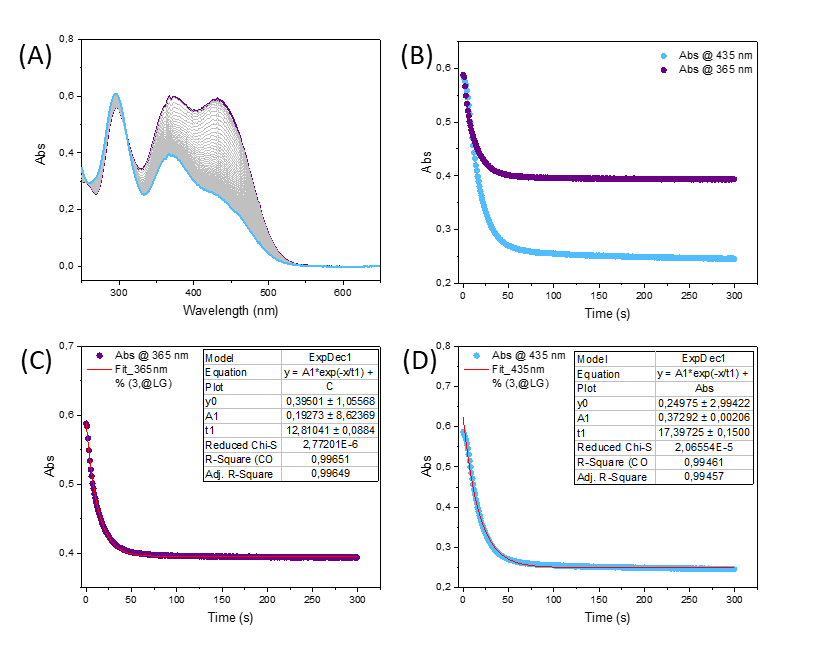


Figure S79: (A) UV-Vis absorbance kinetic spectra of poly4a + 5wt% of 1a (B-D) Photo isomerization kinetic curves of poly4a + 5wt% 1a, fitted.


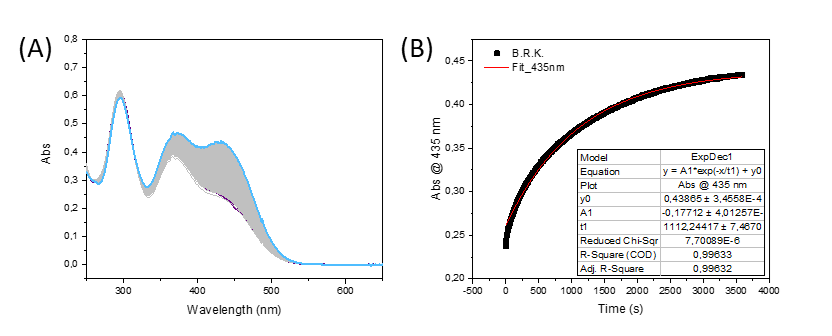


Figure S80: (A) Back relaxation kinetic of poly4a + 5wt% of 1a (B) Back relaxation kinetic curve of poly4a + 5wt% 1a, fitted.


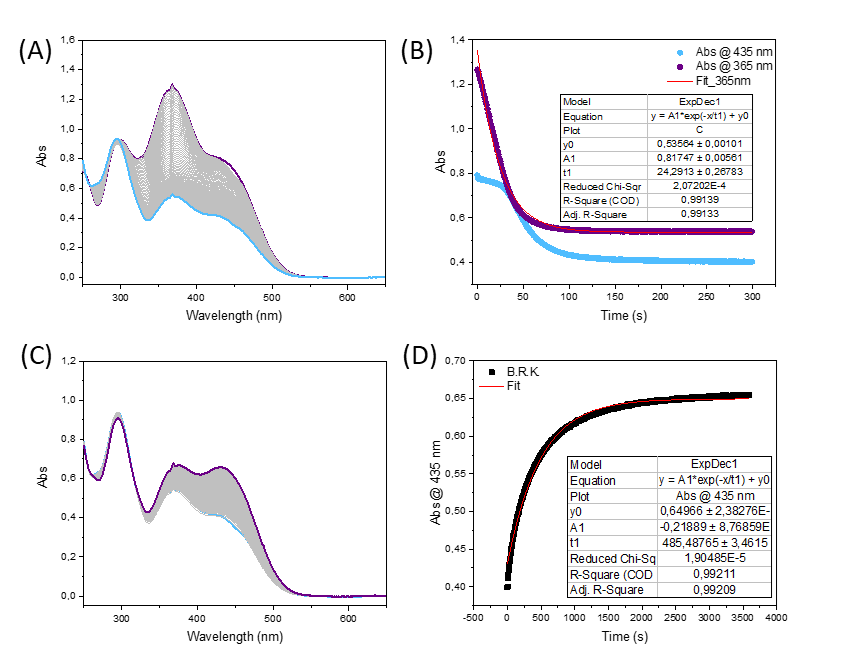


Figure S81: (A) UV-Vis absorbance kinetic spectra of poly4a + 10wt% of 1a (B) Photo isomerization kinetic curves of poly4a + 10wt% 1a (C) Back relaxation kinetic of poly4a + 10wt% of 1a (D) Back relaxation kinetic curve of poly4a + 10wt% 1a, fitted.


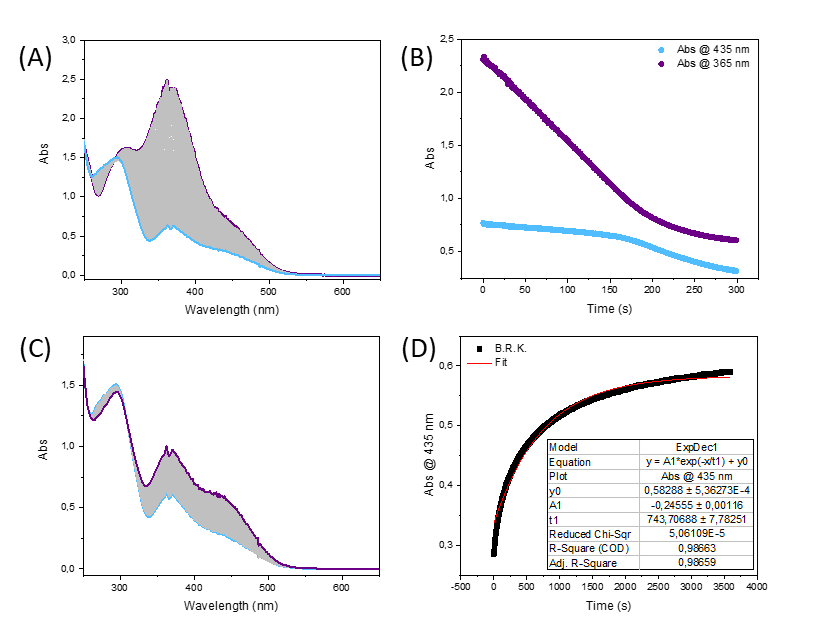


Figure S82: (A) UV-Vis absorbance kinetic spectra of poly4a + 20wt% of 1a (B) Photo isomerization kinetic curves of poly4a + 20wt% 1a (C) Back relaxation kinetic of poly4a + 20wt% of 1a (D) Back relaxation kinetic curve of poly4a + 20wt% 1a, fitted.

**6. Acidochromism**

**6.1. Acidochromism in the presence of trifluoroacetic acid**


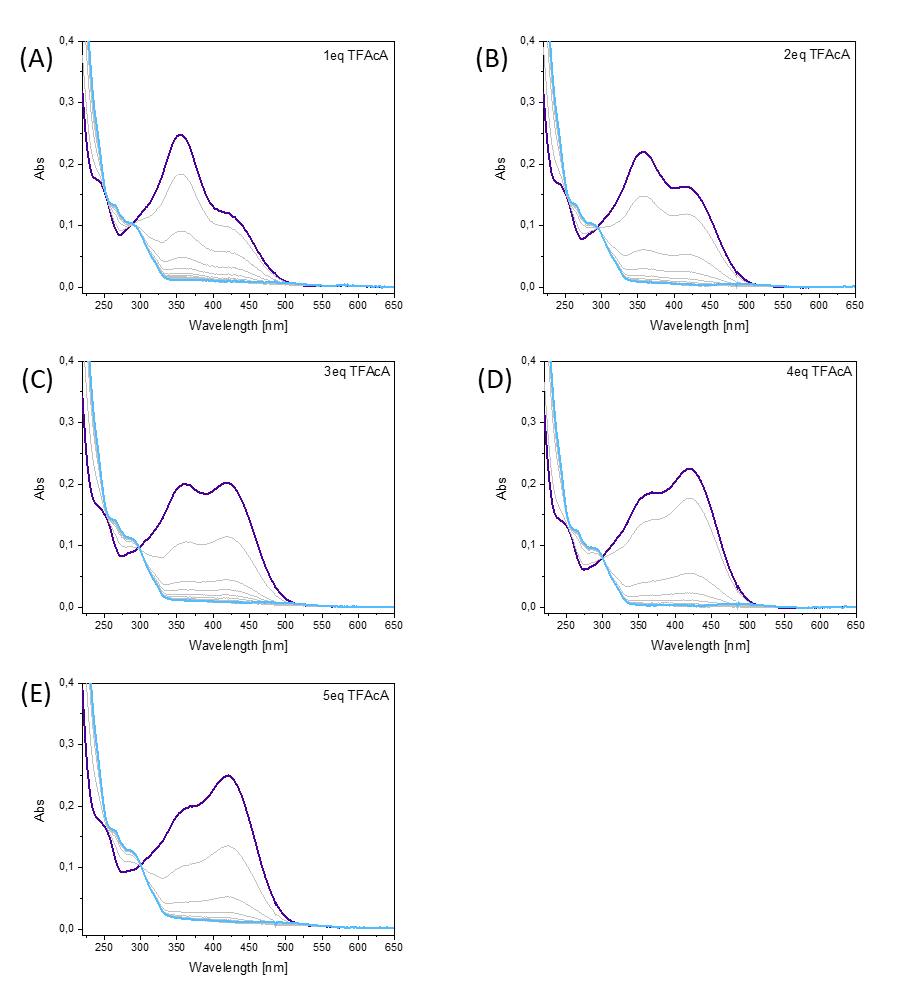


Figure S83: UV-Vis absorption kinetic spectra of 1a in CH_3_CN with increasing amount of Trifluoroacetic acid (TFAcA) at 20°C. Irradiation has been performed at 455nm (T=0 purple, T=100s cyan).

A) 1eq Trifluoroacetic acid B) 2eq Trifluoroacetic acid C) 3eq Trifluoroacetic acid D) 4eq Trifluoroacetic acid E) 5eq Trifluoroacetic acid

**6.2. Acidochromism in the presence of trifluoromethanesulfonic acid**


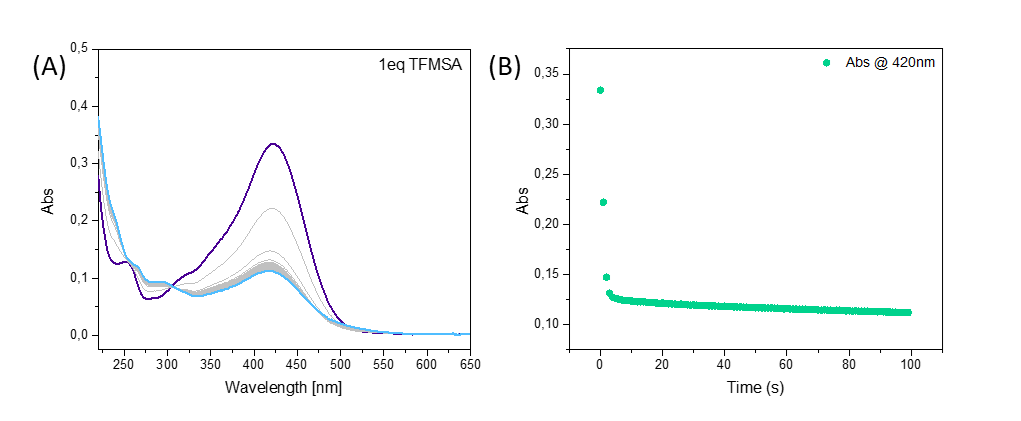


Figure S84: A) UV-Vis absorption kinetic spectra of 1a in CH_3_CN with increasing amount of Trifluoromethanesulfonic acid (TFMSA) at 20°C. Irradiation has been performed at 455nm (T=0 purple, T=100s cyan). B) Change in absorbance at 420 nm over time upon irradiation.

**7. Thermal analysis**

**7.1. Thermogravimetric analysis (TGA)**

Thermogravimetric analysis (TGA) was performed on a Perkin Elmer STA 6000 instrument under a continuous nitrogen flow (30 ml/min ). Samples have been heated from 30 to 700°C at a heating rate of 10°C/min.

Figure S85: TGA profile of poly-4a sodium salt
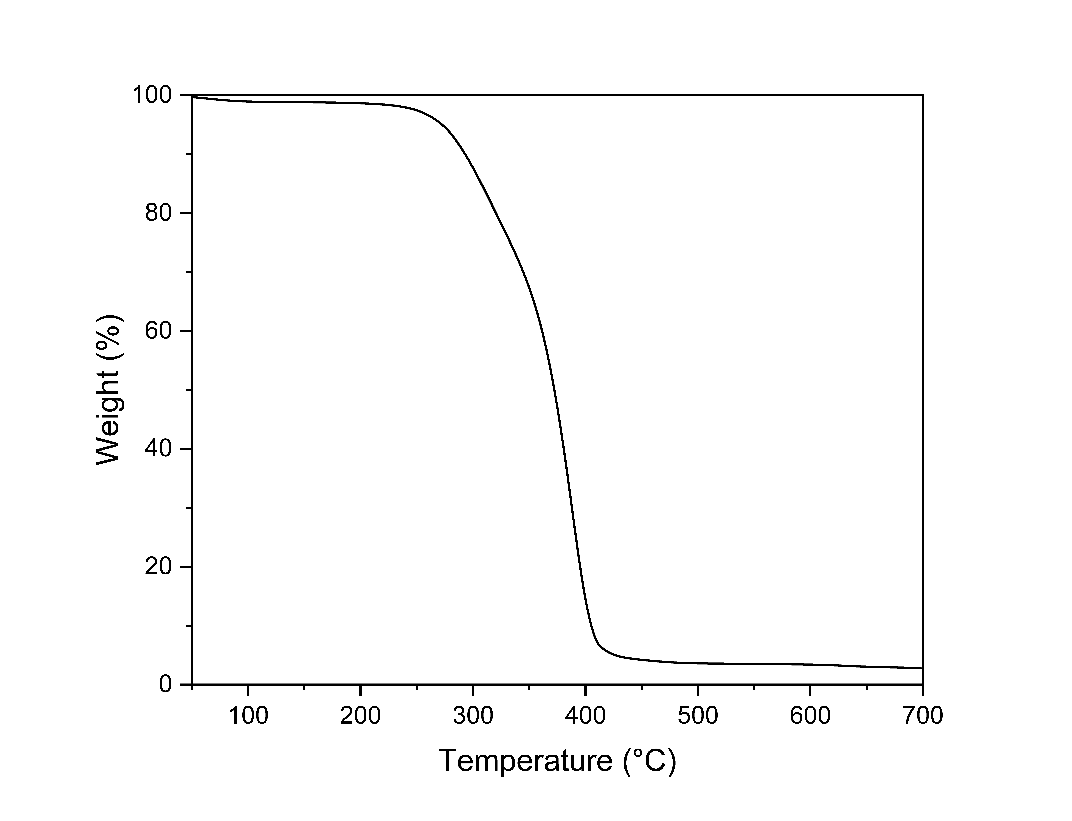


**7.2. Differential Scanning Calorimetry (DSC)**

Differential scanning calorimetry (DSC) measurements were conducted on a TA Instruments DSC Q1000 under nitrogen atmosphere. The samples were heated to 100°C and cooled back to −90°C at a rate of 10°C/min. The second heating cycle was then used for analysis.

Figure S86: DSC profile of poly-4a sodium salt
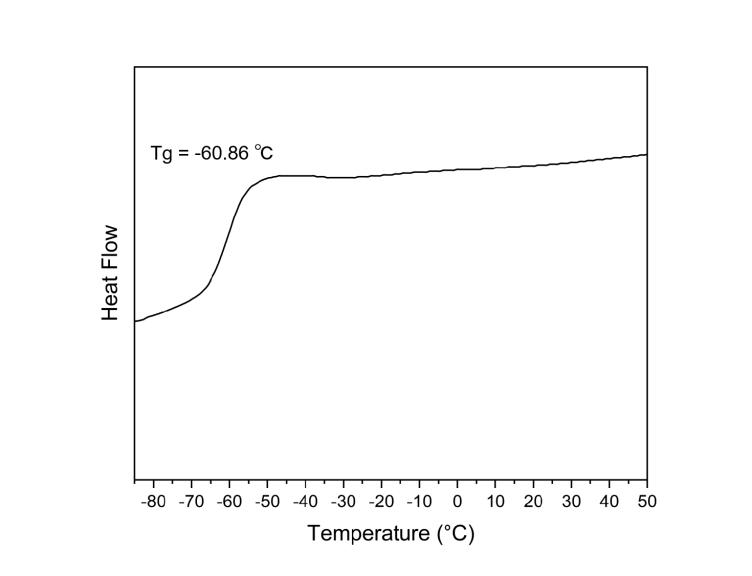
 (exo down)

**8. Gel Permeation Chromatography**

Gel Permeation Chromatography (GPC) was performed in DMF containing 0.01 M LiBr on a Viscotek GPCmax equipped with model 302 TDA detectors and two columns (Agilent Technologies-PolarGel-L and M, 8 µm 30 cm) at a flow rate of 1.0 ml/min and 50 °C. PMMA with narrow polydispersity (Polymer Laboratories) were used as standard to construct a universal calibration curve.

Poly-4a sodium salt was found to have two populations of Mn, results are summarized in the following Table S1, and the curve is shown in Figure S8.1.

Table S1: Mn, Mw, and PDI for poly-4a sodium salt measured by GPC

| Retention Volume | Mn | Mw | PDI |
| --- | --- | --- | --- |
| 12.72 | 30543 | 38632 | 1.26 |
| 15.24 | 2756 | 3036 | 1.1 |


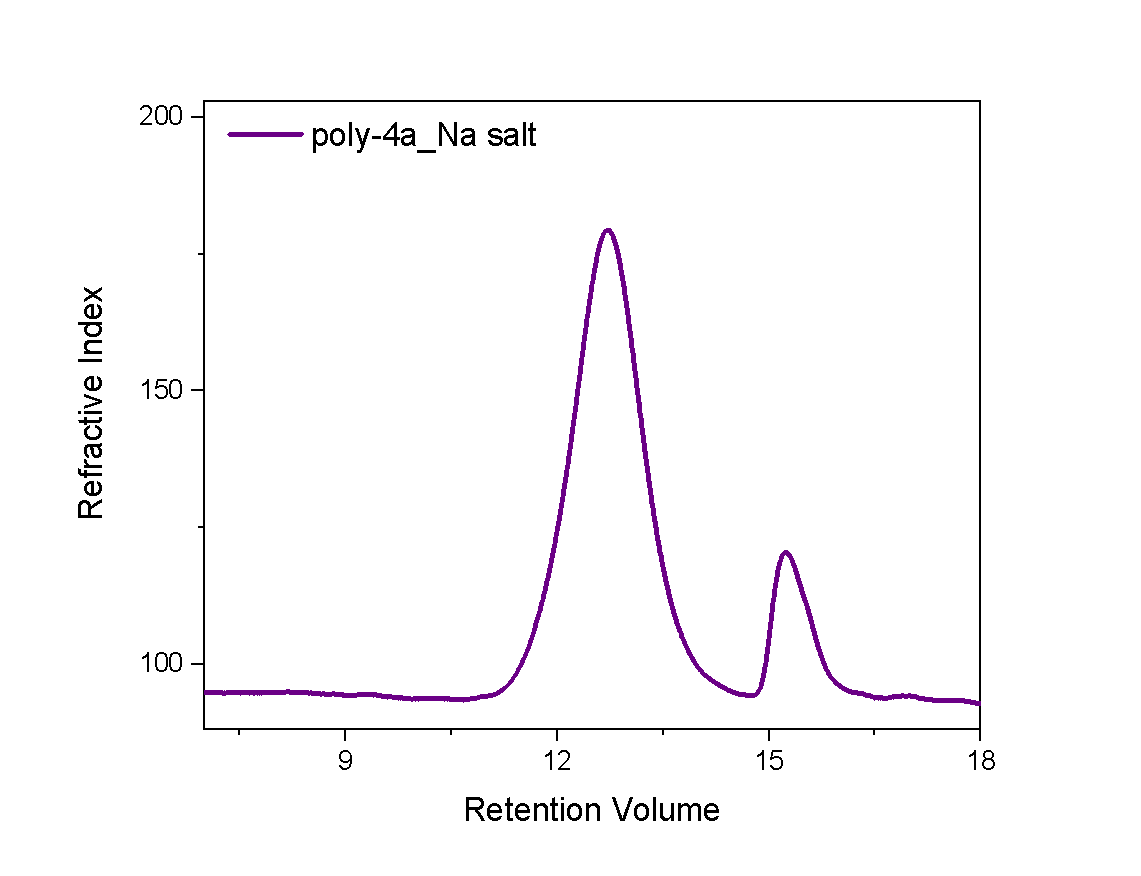


Figure S87: SEC in DMF + LiBr for poly-4a sodium salt

**9. Electrochemical Impedance Spectroscopy**

**9.1. General procedure**

Solutions preparation: to a 10 mg/ml solution of poly-4a sodium salt DOWEX, proton exchange resin, is added. The sample is vigorously shacked using a vortex for one minute, after which the solution is filtered through a 200 μm syringe filter. Then, to 80 μl of the obtained solution of poly-4a, 20 μl of solutions of 1a at different concentrations are added. Quantities are presented in the following Table S2.

Table S2: Conditions for the preparation of the solutions for poly-4a + 1a

| Final wt% of 1a compared to poly-4a | μl of poly-4a solution (10 mg/ml) | Concentration of the solution of 1a (mg/ml) | μl of 1a solution | Final concentration poly-4a (mg/ml) | Final concentration 1a (mg/ml) |
| --- | --- | --- | --- | --- | --- |
| 2.5 | 80 | 1 | 20 | 8 | 0.2 |
| 5 | 80 | 2 | 20 | 8 | 0.4 |
| 10 | 80 | 4 | 20 | 8 | 0.8 |
| 20 | 80 | 8 | 20 | 8 | 1.6 |
| 50 | 80 | 20 | 20 | 8 | 4 |

Film preparation: films of poly-45 + 1a were prepared by casting 80 μl of solution on IDE electrodes at 55 °C. The films have been left drying on the hot plate for one or two minutes, until the methanol has completely evaporated, then placed in a controlled humidity chamber in the dark at RH 100% and 20 °C to equilibrate overnight before measurements.

The irradiation experiment has been performed as follows:

The first EIS scan was performed in the dark, then the LED and air flow were turned on and kept on for all the duration of the experiment. After, the LED was turned off and the window on the cell covered again to allow the sample to relax in the dark, while additional EIS scans were performed, in order to follow the change in resistance during the back relaxation process. A schematic of the experiment setup is depicted in Figure S88.

Figure S88: Schematic of the experimental setups for the measurement of EIS on poly-4a + 1a samples series

**9.2. EIS of polymeric films with 1a**

Figure S89: EIS of poly-4a with increasing amount of 1a in the dark

Figure S90: Overlap of EIS curves for poly-4a with an increasing amount of 1a

(A)

(B)

Figure S91: A) Changes in the Nyquist plots of poly-4a + 5wt% upon cycling. B) Resistance change of poly-4a + 5wt% 1a upon irradiation and relaxation of the polymer film.

**10. DFT Calculations**

**Computational details.** The structures of different isomers of the compound **1a** were optimized in the ground electronic state using density functional theory with the PW6B95 functional and the cc-pVTZ basis set, as implemented in the quantum chemistry package Q-Chem version 5.2. D3 empirical dispersion corrections^[10]^ were included in the computations and the electrostatic solvent free energy was computed using the conductor-like polarizable continuum model^[11]^ considering both methanol and acetonitrile as solvents.

The geometry optimizations were carried out independently for each solvent. All the optimized structures were found to be stable minima via frequency calculations.

The chosen functionals was found to provide reliable predictions on the relative stability of various isomers of a merocyanine-spiropyran switch.^[12]^

**Optimized structures.** Sketches of the structures of the different isomers, optimized in acetonitrile, are shown in Fig. S92.

Fig S92: Optimized structures of the stable isomers of the compound 1a.

For each optimized structure, in acetonitrile or methanol, frequency calculations were carried out to compute Gibbs free energy in the harmonic/rigid rotor approximation for the vibrational/rotational entropies and enthalpies. The relative free energies *G_n_* for the different isomers are reported, for both solvent and acetonitrile, in Table 1, together with their Boltzmann populations

$$p_{n}=\frac{exp(-G_{n}/k_{B}T)}{Z}$$

where *Z* is the normalization constant (partition function) and the index *n* runs over the 10 different isomers of Fig. S92.

Given that the dielectric constants are similar for the two solvents (ε=32.613 and ε=35.688 for methanol and acetonitrile, respectively), nearly identical energies are obtained in the two cases. The energies of the four trans isomers lie within 0.84 kcal/mol, and all of them have a substantial Boltzmann population. In contrast, all cis isomers are found to have a relatively higher energy. The most stable cis species (cis-5) is stabilized by an intramolecular hydrogen bond, clearly visible in Fig. S92.

A fraction of molecules in the closed conformation is also predicted for both solvents, in agreement with the experimental observation (see Table 1 of the main paper). Nevertheless, the computations predict that the majority of molecules (91%) adopt a trans.

Table S3. Gibbs free energy and Boltzmann populations for the different isomers of **1a** in acetonitrile and methanol, calculated at the PW6B95/D3/cc-pVTZ level.

| **Acetonitrile** | | |  | **Methanol** | | |
| --- | --- | --- | --- | --- | --- | --- |
| **Structure** | **Gibbs free energy [kcal/mol]** | ***p_n_*** |  | **Structure** | **Gibbs free energy [kcal/mol]** | ***p_n_*** |
| trans-1 | 0.49 | 0.15 |  | trans-1 | 0.49 | 0.15 |
| trans-2 | 0.00 | 0.34 |  | trans-2 | 0.00 | 0.34 |
| trans-3 | 0.00 | 0.34 |  | trans-3 | 0.01 | 0.34 |
| trans-4 | 0.83 | 0.08 |  | trans-4 | 0.84 | 0.08 |
| *total trans population* | | 0.91 |  | *total trans population* | | 0.91 |
| cis-1 | 5.98 | 0.00 |  | cis-1 | 5.99 | 0.00 |
| cis-2 | 6.24 | 0.00 |  | cis-2 | 6.25 | 0.00 |
| cis-3 | 10.08 | 0.00 |  | cis-3 | 10.08 | 0.00 |
| cis-4 | 10.24 | 0.00 |  | cis-4 | 10.24 | 0.00 |
| cis-5 | 2.82 | 0.01 |  | cis-5 | 2.82 | 0.01 |
| *total cis population* | | 0.01 |  | *total cis population* | | 0.01 |
| closed-1 | 1.34 | 0.04 |  | closed-1 | 1.34 | 0.04 |
| closed-2 | 3.31 | 0.00 |  | closed-2 | 3.29 | 0.00 |
| closed-3 | 1.23 | 0.04 |  | closed-3 | 1.22 | 0.04 |
| *total closed population* | | 0.08 |  | *total closed population* | | 0.08 |

To investigate to which extent the present results are sensitive to the electronic structure method, we also tested the B3LYP functional with the D3(BJ) dispersion correction and the same basis set (cc-pVTZ). The molecular structures were reoptimized, to compute the Gibbs free energy and the Boltzmann pop1ulation. The results are shown in Table 2. In this case, the ratio between open and closed forms is 99:1, in contrast to 92:8 predicted by PW6B95. In particular, the energies of the trans isomer are predicted to be more close each other, and the cis-5 isomer is found to be significantly more stable, probably due to a higher hydrogen bond strength given by the D3(BJ) correction.

Table S4. Gibbs free energy and Boltzmann populations for the different isomers of **1a** in acetonitrile and methanol, calculated at the B3LYP/D3(BJ)/cc-pVTZ level.

| **Acetonitrile** | | |  | **Methanol** | | |
| --- | --- | --- | --- | --- | --- | --- |
| **Structure** | **Gibbs free energy [kcal/mol]** | ***p_n_*** |  | **Structure** | **Gibbs free energy [kcal/mol]** | ***p_n_*** |
| trans-1 | 0.41 | 0.16 |  | trans-1 | 0.41 | 0.16 |
| trans-2 | 0.00 | 0.32 |  | trans-2 | 0.00 | 0.32 |
| trans-3 | 0.01 | 0.32 |  | trans-3 | 0.01 | 0.32 |
| trans-4 | 0.58 | 0.12 |  | trans-4 | 0.58 | 0.12 |
| *total trans population* | | 0.92 |  | *total trans population* | | 0.92 |
| cis-1 | 5.84 | 0.00 |  | cis-1 | 5.84 | 0.00 |
| cis-2 | 6.30 | 0.00 |  | cis-2 | 6.30 | 0.00 |
| cis-3 | 9.63 | 0.00 |  | cis-3 | 9.63 | 0.00 |
| cis-4 | 9.46 | 0.00 |  | cis-4 | 9.46 | 0.00 |
| cis-5 | 0.89 | 0.07 |  | cis-5 | 0.88 | 0.07 |
| *total cis population* | | 0.07 |  | *total cis population* | | 0.07 |
| closed-1 | 2.91 | 0.00 |  | closed-1 | 2.90 | 0.00 |
| closed-2 | 5.07 | 0.00 |  | closed-2 | 5.06 | 0.00 |
| closed-3 | 2.72 | 0.01 |  | closed-3 | 2.70 | 0.01 |
| *total closed population* | | 0.01 |  | *total closed population* | | 0.01 |

**Structure of the various isomers of 1a, optimized at the PW6B95-D3/cc-pVTZ level.**

*Trans-1*

N 1.08706 -0.07682 -1.37135

C 0.88868 -0.01850 -2.75707

C -0.45676 0.14725 -3.08201

C -1.22148 0.20750 -1.79497

C -0.85696 0.22503 -4.39588

H -1.89674 0.35277 -4.65606

C 0.11234 0.13551 -5.39032

H -0.17842 0.19349 -6.42682

C 1.45129 -0.02800 -5.06251

H 2.18534 -0.09454 -5.84956

C 1.85911 -0.10736 -3.73850

H 2.89760 -0.23492 -3.47874

C -0.07320 0.04486 -0.81340

C -2.22894 -0.93583 -1.65978

C -1.92362 1.55299 -1.59827

C -0.30431 0.03307 0.61200

C 0.68755 -0.13474 1.50129

C 0.64809 -0.17966 2.94556

C -0.50161 -0.01686 3.73583

C -0.42061 -0.08193 5.11819

C 0.79039 -0.30811 5.74232

C 1.94156 -0.47129 4.98732

C 1.85696 -0.40426 3.61408

O -1.68400 0.20460 3.12150

H -2.37624 0.30601 3.78050

H -1.74612 -1.90064 -1.78216

H -2.99846 -0.83531 -2.42010

H -2.70788 -0.90738 -0.68483

H -1.22098 2.37706 -1.67692

H -2.40045 1.59379 -0.62270

H -2.69019 1.67823 -2.35784

H -1.32204 0.16119 0.93134

H 1.68014 -0.26309 1.09035

H -1.32138 0.04801 5.69901

H 0.83165 -0.35567 6.81839

H 2.89040 -0.64718 5.46582

H 2.74542 -0.52894 3.01448

*Trans-2*

N 1.94015 -0.42130 -1.23722

C 1.78460 -0.35393 -2.62549

C 0.47794 -0.03337 -2.98004

C -0.31325 0.12781 -1.71418

C 0.11733 0.08242 -4.30285

H -0.89405 0.33079 -4.58679

C 1.09052 -0.12907 -5.27386

H 0.83176 -0.04397 -6.31691

C 2.39369 -0.44913 -4.91589

H 3.13073 -0.60882 -5.68644

C 2.76008 -0.56641 -3.58346

H 3.76974 -0.81450 -3.29865

C 0.78923 -0.15888 -0.69866

C -1.44944 -0.89747 -1.65586

C -0.85642 1.55502 -1.59633

H -1.06146 -1.90998 -1.71247

H -2.11585 -0.73832 -2.49907

H -2.02940 -0.80054 -0.74457

H -0.04892 2.28049 -1.62224

H -1.41506 1.69323 -0.67693

H -1.52261 1.75364 -2.43129

C 0.67258 -0.16153 0.73389

C -0.44545 0.08157 1.43383

C -0.56805 0.08214 2.87856

C -1.83441 0.26197 3.45366

C -1.99421 0.26836 4.83132

C 0.51730 -0.08423 3.74115

C 0.36364 -0.07759 5.10994

C -0.90038 0.09911 5.65562

O -2.88885 0.42402 2.62042

H -3.69363 0.53724 3.13212

H 1.59808 -0.38934 1.24147

H -1.35732 0.29517 0.90558

H -2.98106 0.40564 5.24661

H 1.50178 -0.21499 3.32298

H 1.22002 -0.20616 5.75064

H -1.03495 0.10671 6.72507

*Trans-3*

N 0.84777 -0.02273 -1.39797

C 0.60495 0.04007 -2.77627

C -0.75203 0.18990 -3.05751

C -1.47708 0.23161 -1.74681

C -1.19455 0.27106 -4.35742

H -2.24354 0.38697 -4.58388

C -0.25602 0.20038 -5.38243

H -0.58005 0.26125 -6.40884

C 1.09455 0.05168 -5.09818

H 1.80413 -0.00070 -5.90840

C 1.54504 -0.03049 -3.78830

H 2.59272 -0.14613 -3.56205

C -0.29612 0.08016 -0.80314

C -2.46258 -0.92792 -1.58857

C -2.19293 1.56527 -1.52142

C -0.47364 0.05678 0.62843

C 0.53974 -0.10157 1.49159

C 0.45058 -0.13227 2.93612

C -0.73919 0.09296 3.63296

C -0.79243 0.05834 5.00845

C 0.36350 -0.20560 5.73096

C 1.55592 -0.43075 5.07430

C 1.60504 -0.39485 3.68838

H -1.96876 -1.88445 -1.73029

H -3.25500 -0.83522 -2.32597

H -2.91419 -0.91216 -0.60038

H -1.50512 2.40018 -1.61503

H -2.64301 1.59374 -0.53282

H -2.98243 1.68323 -2.25830

H -1.48756 0.16935 0.98034

H 1.52764 -0.22372 1.07633

H -1.72409 0.23766 5.51863

H 0.33663 -0.23491 6.80816

H 2.45957 -0.63706 5.62744

O 2.75993 -0.61425 3.01913

H -1.63771 0.30489 3.07669

H 3.47040 -0.77832 3.64429

*Trans-4*

N 1.71505 -0.33670 -1.33113

C 1.51557 -0.26697 -2.71358

C 0.19070 0.01905 -3.02647

C -0.56494 0.15510 -1.73595

C -0.21344 0.12937 -4.33712

H -1.23948 0.35084 -4.58901

C 0.73483 -0.05198 -5.33855

H 0.44163 0.02918 -6.37278

C 2.05670 -0.33675 -5.02209

H 2.77396 -0.47305 -5.81549

C 2.46676 -0.44856 -3.70185

H 3.49115 -0.66958 -3.44907

C 0.57515 -0.10747 -0.75485

C -1.67211 -0.89944 -1.64859

C -1.14009 1.56840 -1.59991

H -1.25966 -1.90123 -1.72110

H -2.36722 -0.75498 -2.47103

H -2.22723 -0.82146 -0.72022

H -0.35143 2.31339 -1.64457

H -1.67937 1.69277 -0.66721

H -1.83145 1.75213 -2.41768

C 0.51175 -0.12396 0.68302

C -0.59379 0.09790 1.41366

C -0.76752 0.10642 2.85166

C -2.05267 0.37697 3.33521

C -2.34644 0.41401 4.68051

C 0.23743 -0.13194 3.80331

C -0.05428 -0.09626 5.15827

C -1.33512 0.17434 5.59751

H 1.45196 -0.33473 1.15914

H -1.51285 0.30548 0.88938

H -3.34893 0.62622 5.01230

O 1.49090 -0.39591 3.37448

H 0.73923 -0.28417 5.86597

H -1.53938 0.19728 6.65561

H 2.06748 -0.53549 4.13060

H -2.83186 0.56216 2.61196

*Cis-1*

C -4.68316 1.50852 -0.62074

C -3.55583 2.31196 -0.48458

C -2.34674 1.70555 -0.23169

C -4.59032 0.12810 -0.50237

C -3.37340 -0.48667 -0.24698

C -2.25847 0.32136 -0.11524

C -0.97122 2.26023 -0.03045

C -0.20018 0.95961 0.18628

N -0.94605 -0.09656 0.14096

C -0.46302 3.00964 -1.26324

C -0.89859 3.16419 1.20251

C 1.22205 1.03016 0.41122

C 2.23173 0.13380 0.44200

C 2.33539 -1.30366 0.29832

C 1.26716 -2.20312 0.35209

C 1.45721 -3.56479 0.23803

C 2.73605 -4.07006 0.06643

C 3.81657 -3.21156 0.01232

C 3.62557 -1.84456 0.12883

H -5.64097 1.96158 -0.81959

H -3.63448 3.38455 -0.57589

H -5.47802 -0.47407 -0.61134

H -3.29484 -1.55788 -0.15518

H -0.49842 2.37980 -2.14698

H 0.56069 3.34101 -1.11306

H -1.08224 3.88515 -1.43779

H -1.23340 2.64125 2.09320

H -1.53522 4.03152 1.05166

H 0.11690 3.51430 1.36422

H 1.55502 2.05007 0.53798

H 3.19578 0.59628 0.58556

H 0.27880 -1.80360 0.48379

H 0.61170 -4.23107 0.28668

H 2.89679 -5.13231 -0.02384

H 4.81754 -3.59310 -0.12127

O 4.67383 -0.99025 0.07121

H 5.49122 -1.48766 -0.01098

*Cis-2*

C -4.25191 1.03965 -0.67196

C -3.26453 1.90556 -0.21256

C -1.94994 1.51215 -0.31447

C -3.91822 -0.18978 -1.22358

C -2.59333 -0.58822 -1.33165

C -1.62025 0.27760 -0.86897

C -0.66831 2.18472 0.07313

C 0.31362 1.10136 -0.34174

N -0.23246 0.06448 -0.87728

C -0.44012 3.47634 -0.71609

C -0.57509 2.46040 1.57448

C 1.74157 1.26647 -0.16364

C 2.65573 0.31146 0.03244

C 2.46521 -1.12642 0.22481

C 1.52445 -1.67101 1.10555

C 1.41493 -3.04582 1.24984

C 2.24203 -3.89531 0.54004

C 3.20349 -3.37573 -0.31038

C 3.31321 -2.00549 -0.44482

H -5.28885 1.32562 -0.59947

H -3.53068 2.86052 0.21433

H -4.70080 -0.84439 -1.57234

H -2.32687 -1.54163 -1.75849

H -0.47516 3.29453 -1.78601

H 0.52297 3.91283 -0.46686

H -1.21342 4.19601 -0.46260

H -0.72400 1.55110 2.14771

H -1.33284 3.18392 1.86188

H 0.40093 2.86945 1.82173

H 2.09490 2.28670 -0.20756

H 3.68987 0.62886 0.04055

O 0.75386 -0.83009 1.83206

H 0.67881 -3.44153 1.93367

H 2.14075 -4.96138 0.66387

H 3.86329 -4.02897 -0.85672

H 4.06424 -1.58938 -1.09865

H 0.17810 -1.34038 2.40693

*Cis-3*

C 4.08903 1.44067 -1.81038

C 2.71236 1.52534 -1.63447

C 2.05830 0.45557 -1.06590

C 2.76361 -0.67423 -0.67256

C 4.13364 -0.76831 -0.83599

C 4.78923 0.30880 -1.41299

C 0.61141 0.24365 -0.72361

C 0.70796 -1.17222 -0.13726

N 1.90888 -1.65197 -0.15094

C -0.37358 -2.10776 0.15467

C -1.61433 -1.91651 0.60389

C -2.18563 -0.69809 1.17932

C -3.31139 -0.10588 0.60778

C -3.86275 1.03724 1.16225

C -3.31506 1.57840 2.31218

C -2.21783 0.98440 2.91215

C -1.66182 -0.14537 2.34104

O -3.80624 -0.68115 -0.51566

H -4.57611 -0.19340 -0.81959

C -0.28041 0.21510 -1.96891

C 0.15008 1.37147 0.20523

H 4.62081 2.26353 -2.26016

H 2.17604 2.41060 -1.94076

H 4.66821 -1.65171 -0.52641

H 5.85694 0.26792 -1.55671

H -0.09742 -3.12406 -0.08749

H -2.26835 -2.77875 0.59585

H -4.71893 1.49506 0.69057

H -3.75369 2.46571 2.73920

H -1.79602 1.40001 3.81203

H -0.80150 -0.61499 2.79056

H 0.05647 -0.53316 -2.68011

H -0.25835 1.18776 -2.45227

H -1.30585 -0.00811 -1.68691

H 0.61846 1.30508 1.18163

H -0.92449 1.37061 0.33141

H 0.43447 2.31727 -0.24754

*Cis-4*

C 3.69565 1.50750 -2.47575

C 2.67819 1.68303 -1.54446

C 2.04135 0.56591 -1.05261

C 2.40676 -0.70212 -1.48553

C 3.41285 -0.88985 -2.41557

C 4.05512 0.23686 -2.90643

C 0.90540 0.41678 -0.08157

C 0.79537 -1.11513 -0.07437

N 1.65889 -1.69519 -0.84205

C 0.06749 -1.96237 0.86610

C -1.10924 -1.80131 1.47185

C -2.14544 -0.81393 1.16942

C -2.73633 -0.76274 -0.08666

C -3.74060 0.14391 -0.37068

C -4.16204 1.02060 0.61437

C -3.59767 0.97928 1.87740

C -2.60524 0.05515 2.15885

C -0.29539 1.21528 -0.59958

C 1.26984 0.90444 1.32371

H 4.21225 2.36773 -2.86988

H 2.40075 2.67417 -1.21910

H 3.68577 -1.88008 -2.74268

H 4.84512 0.12639 -3.63177

H 0.62888 -2.84918 1.12363

H -1.35886 -2.51841 2.24293

H -4.18576 0.16972 -1.35132

H -4.93712 1.74019 0.40568

H -3.92266 1.66014 2.64940

O -2.01484 -0.03150 3.37607

H -0.70132 0.78355 -1.50837

H 0.04248 2.22406 -0.82075

H -1.08253 1.28037 0.14017

H 2.15377 0.39965 1.70192

H 0.44429 0.72023 2.00622

H 1.46485 1.97282 1.29741

H -2.40967 0.60625 3.97619

H -2.39025 -1.44701 -0.84491

*Cis-5*

C -4.21855 1.30246 1.47189

C -3.16716 2.17778 1.22202

C -1.99482 1.66423 0.71631

C -4.08994 -0.05657 1.21802

C -2.91049 -0.57986 0.70911

C -1.87429 0.30093 0.46700

C -0.69961 2.31522 0.33288

C 0.07407 1.09170 -0.12708

N -0.60404 -0.00623 -0.04092

C -0.87556 3.31264 -0.81517

C -0.01255 2.99143 1.52100

C 1.43652 1.19630 -0.56924

C 2.39111 0.26026 -0.73597

C 2.44883 -1.19265 -0.64864

C 1.49722 -2.10682 -1.13511

C 1.76035 -3.47296 -1.07240

C 2.93437 -3.95230 -0.53628

C 3.88893 -3.06498 -0.05535

C 3.64733 -1.71431 -0.13760

H -5.14641 1.68288 1.86729

H -3.27487 3.23316 1.41949

H -4.91983 -0.71451 1.41922

H -2.80241 -1.63332 0.50889

H -1.34462 2.84333 -1.67438

H 0.08509 3.71871 -1.11868

H -1.50337 4.13459 -0.48365

H 0.13668 2.29173 2.33748

H -0.62934 3.81200 1.87583

H 0.95224 3.39255 1.22397

H 1.77576 2.21545 -0.67881

H 3.37143 0.68120 -0.91633

O 0.35921 -1.72283 -1.73837

H 1.01312 -4.14446 -1.46311

H 3.11094 -5.01530 -0.49869

H 4.81561 -3.42649 0.35836

H 4.39738 -1.01634 0.20159

H -0.09738 -1.06399 -1.13751

*Closed-1*

C 4.04583 1.67515 -0.09146

C 4.39928 0.52640 -0.77952

C 2.70327 2.01565 0.05482

C 1.74316 1.19436 -0.48715

C 2.10938 0.03949 -1.17136

C 3.43594 -0.31033 -1.33156

C 0.24429 1.33106 -0.54828

C -0.15578 -0.14560 -0.88318

N 0.97156 -0.60534 -1.64817

C -1.44636 -0.29457 -1.61149

C -2.55803 -0.69072 -1.00225

O -0.15227 -0.91958 0.34376

C -1.29924 -1.17072 1.01573

C -2.54220 -1.06658 0.39370

C -1.20016 -1.58972 2.33044

C -2.35117 -1.88409 3.04068

C -3.68469 -1.37022 1.12342

C -3.59759 -1.77070 2.44350

H 1.01728 -1.59781 -1.80625

C -0.12183 2.24817 -1.71798

C -0.40531 1.84110 0.72381

H 4.81066 2.30473 0.33299

H 5.44113 0.26843 -0.88527

H 2.42243 2.91041 0.58927

H 3.71689 -1.20757 -1.85924

H -1.43603 -0.02451 -2.65363

H -3.49037 -0.75522 -1.54162

H -0.22573 -1.67263 2.78344

H -2.27140 -2.19953 4.06850

H -4.64545 -1.29199 0.63799

H -4.49058 -1.99852 3.00150

H 0.28140 1.87182 -2.65390

H 0.28910 3.23696 -1.53856

H -1.20064 2.33733 -1.81526

H -0.09969 1.26625 1.59072

H -1.48925 1.79868 0.64020

H -0.12521 2.87848 0.88487

*Closed-2*

C -2.12357 -1.04519 0.01232

C -1.48116 -1.71830 -1.02302

C -2.01557 -2.87068 -1.56854

C -3.21982 -3.33643 -1.05572

C -3.86861 -2.67251 -0.02733

C -3.31783 -1.51251 0.51019

C 0.06074 -0.19768 -0.25957

H -1.51129 -3.39558 -2.36391

H -3.65091 -4.23692 -1.46364

H -4.79778 -3.05749 0.35979

H -3.81775 -0.99248 1.31315

H 0.43521 -1.62478 -1.74157

N -0.31283 -1.05409 -1.38156

C 0.91798 -0.95221 0.70339

C 2.07268 -0.48694 1.16227

H 0.52801 -1.90991 1.01029

H 2.67101 -1.06164 1.85231

C 2.55084 0.81037 0.74437

C 1.82467 1.47796 -0.23929

C 3.69707 1.40913 1.25247

C 2.24110 2.71003 -0.71164

C 4.11565 2.64454 0.79587

H 4.25850 0.88758 2.01242

C 3.38432 3.28977 -0.18985

H 1.66544 3.19981 -1.47985

H 5.00454 3.09993 1.19976

H 3.70182 4.25280 -0.55579

O 0.71657 0.94059 -0.80352

C -1.33286 0.19164 0.34819

C -1.91848 1.37783 -0.42008

H -1.35393 2.28356 -0.22188

H -1.90898 1.19412 -1.49019

H -2.94585 1.53144 -0.10299

C -1.26435 0.52143 1.82775

H -0.94141 -0.32998 2.41766

H -0.57193 1.34237 2.00084

H -2.24421 0.82970 2.18206

*Closed-3*

-2.24451 0.09027 -0.25316

C -1.79387 -0.63870 0.84334

C -2.48574 -1.74068 1.30703

C -3.65088 -2.10338 0.64052

C -4.10687 -1.38655 -0.45310

C -3.39881 -0.27484 -0.90386

C -0.05751 0.76332 0.30228

H -2.13141 -2.30652 2.15360

H -4.20363 -2.96456 0.98119

H -5.00966 -1.69075 -0.95706

H -3.75136 0.28781 -1.75489

H -0.00099 -0.60820 1.89199

N -0.63510 -0.05507 1.34131

C 0.84295 1.80196 0.87780

C 2.15932 1.62147 0.92695

H 0.37922 2.66482 1.32236

H 2.80014 2.35007 1.39924

C 2.76218 0.43731 0.36068

C 1.94695 -0.39208 -0.40857

C 4.10120 0.09828 0.52012

C 2.45734 -1.52982 -1.00885

C 4.61822 -1.03895 -0.06959

H 4.73249 0.74226 1.11303

C 3.79240 -1.84890 -0.83629

H 1.80498 -2.14843 -1.60330

H 5.65685 -1.29331 0.06239

H 4.18866 -2.73692 -1.30179

O 0.65528 -0.06847 -0.64326

C -1.32001 1.26354 -0.46745

C -1.92306 2.48474 0.23103

H -1.30165 3.36447 0.08724

H -2.04299 2.31117 1.29678

H -2.90031 2.68775 -0.19566

C -1.04418 1.59440 -1.92219

H -0.70589 0.72657 -2.47473

H -0.28781 2.37168 -1.99846

H -1.95394 1.96690 -2.38526

**11. Bibliography**

[1] C. Feldmeier, H. Bartling, E. Riedle, R. M. Gschwind, *Journal of Magnetic Resonance* **2013**, *232*, 39–44.

[2] C. Ma, J. Dong, M. Viviani, I. Tulini, N. Pontillo, S. Maity, Y. Zhou, W. H. Roos, K. Liu, A. Herrmann, G. Portale, *Sci. Adv* **2020**, *6*, 810–827.

[3] E. Deniz, S. Sortino, F. M. Raymo, *Journal of Physical Chemistry Letters* **2010**, *1*, 1690–1693.

[4] R. B. Mujumdar, L. A. Ernst, S. R. Mujumdar, C. J. Lewis, A. S. Waggoner, *Bioconjug Chem* **1993**, *4*.

[5] A. H. Hofman, R. Fokkink, M. Kamperman, *Polym Chem* **2019**, *10*, 6109–6115.

[6] C. Berton, D. M. Busiello, S. Zamuner, E. Solari, R. Scopelliti, F. Fadaei-Tirani, K. Severin, C. Pezzato, *Chem Sci* **2020**, *11*, 8457–8468.

[7] L. Wimberger, S. K. K. Prasad, M. D. Peeks, J. Andreásson, T. W. Schmidt, J. E. Beves, *J Am Chem Soc* **2021**, *143*, 20758–20768.

[8] K. Stranius, K. Börjesson, *Sci Rep* **2017**, *7*.

[9] M. Mansø, A. U. Petersen, Z. Wang, P. Erhart, M. B. Nielsen, K. Moth-Poulsen, *Nat Commun* **2018**, *9*.

[10] S. Grimme, S. Ehrlich, L. Goerigk, *J Comput Chem* **2011**, *32*, 1456–1465.

[11] M. Cossi, N. Rega, G. Scalmani, V. Barone, *J Comput Chem* **2002**, *24*, 669–681.

[12] P. B. Markworth, B. D. Adamson, N. J. A. Coughlan, L. Goerigk, E. J. Bieske, *Physical Chemistry Chemical Physics* **2015**, *17*, 25676–25688.
